# Salazinic Acid and Norlobaridone from the Lichen *Hypotrachyna cirrhata*: Antioxidant Activity, α-Glucosidase Inhibitory and Molecular Docking Studies

**DOI:** 10.3390/molecules28237840

**Published:** 2023-11-29

**Authors:** Tatapudi Kiran Kumar, Bandi Siva, Basani Kiranmai, Vidya Jyothi Alli, Surender Singh Jadav, Araveeti Madhusudana Reddy, Joël Boustie, Françoise Le Devehat, Ashok Kumar Tiwari, Katragadda Suresh Babu

**Affiliations:** 1 Department of Natural Products & Medicinal Chemistry, CSIR-Indian Institute of Chemical Technology, Uppal Road, Tarnaka, Hyderabad 500007, India; kirantatapudi09@gmail.com (T.K.K.); bandishiva2008@gmail.com (B.S.); basanikiranmai@gmail.com (B.K.); vidyaalli50@gmail.com (V.J.A.); surenderjs@iict.res.in (S.S.J.); tiwari@iict.res.in (A.K.T.); 2 Academy of Scientific and Innovative Research (AcSIR), Ghaziabad 201002, India; 3 Department of Botany, Yogi Vemana University, Vemanapuram, Kadapa 516003, India; grassced@yahoo.com; 4 CNRS (Centre National de la Recherché Scientifique), ISCR (Institut des Sciences Chimiques de Rennes)-UMR6226, University of Rennes, 35000 Rennes, France; francoise.le-devehat@univ-rennes1.fr

**Keywords:** *Everniatrum cirrhatum* (Fr.) Hale, secondary metabolites, antioxidant, intestinal α-glucosidase inhibition, UPLC-QToF-MS/MS

## Abstract

The present study was intended for the identification of secondary metabolites in acetone extract of the lichen *Hypotrachyna cirrhata* using UPLC-ESI-QToF-MS/MS and the detection of bioactive compounds. This study led to the identification of 22 metabolites based on their MS/MS spectra, accurate molecular masses, molecular formula from a comparison of the literature database (DNP), and fragmentation patterns. In addition, potent antioxidant and α-glucosidase inhibitory potentials of acetone extract of *H. cirrhata* motivated us to isolate 10 metabolites, which were characterized as salazinic acid (**11**), norlobaridone (**12**), atranorin (**13**), lecanoric acid (**14**), lichesterinic acid (**15**), protolichesterinic acid (**16**), methyl hematommate (**17**), iso-rhizonic acid (**18**), atranol (**19**), and methylatratate (**20)** based on their spectral data. All these isolates were assessed for their free radicals scavenging, radical-induced DNA damage, and intestinal α-glucosidase inhibitory activities. The results indicated that norlobaridone (**12**), lecanoric acid (**14**), methyl hematommate **(17**), and atranol (**19**) showed potent antioxidant activity, while depsidones (salazinic acid (**11**), norlobaridone (**12**)) and a monophenolic compound (iso-rhizonic acid, (**18**)) displayed significant intestinal α-glucosidase inhibitory activities (*p* < 0.001), which is comparable to standard acarbose. These results were further correlated with molecular docking studies, which indicated that the alkyl chain of norlobaridione (**12**) is hooked into the finger-like cavity of the allosteric pocket; moreover, it also established Van der Waals interactions with hydrophobic residues of the allosteric pocket. Thus, the potency of norlobaridone to inhibit α-glucosidase enzyme might be associated with its allosteric binding. Also, MM-GBSA (Molecular Mechanics-Generalized Born Surface Area) binding free energies of salazinic acid (**11**) and norlobaridone (**12**) were superior to acarbose and may have contributed to their high activity compared to acarbose.

## 1. Introduction

The unique symbiosis relationship in fungi forming a lichen life form [1] generates a number of novel/complex secondary metabolites which are responsible for a wide range of biological activities [2]. Lichens have been widely used in traditional medicines for centuries and have now attracted worldwide attention for exploration of their potential as medicines, food, cosmetic, and perfumery applications [3]. Several lichens are currently being used in traditional Indian and Chinese herbal medicinal preparations. Among them, the foliose lichen, *Hypotrachyna cirrhata* (Fr.) Divakar, A. Crespo, Sipman, Elix and Lumbsch (=*Everniatrum cirrhatum* (Fr.) Hale) (Family: Parmeliaceae) growing in the tropical Himalayas and central India finds extensive applications in traditional Indian systems of medicine [4]. In Ayurveda, it is used due to its laxative, astringent, aphrodisiac, and carminative properties. In folklore medicine, particularly in Madhya Pradesh and Sikkim states of India, the whole boiled lichen of *H. cirrhata* is used as a spice, vegetable, and antiseptic agent to heal wounds [5]. The antioxidant and cytotoxic activities of the crude extracts [6] and anti-obesity activity, in terms of pancreatic lipase inhibitory activity of H. cirrhata, have been reported as preliminary results [7]. Sepulveda et al. recently studied the green ultrasonic extraction methods using ethyl lactate as an extracting solvent instead of methanol and assessed in vitro biological activities [8]. Phenolic-type compounds, such as depsides and depsidones, constitute the main secondary metabolites of the species, which are also useful as chemotaxonomic markers [9]. Atranorin, (+)-usnic acid, lecanoric acid, diffractaic acid, lobaric acid, stictic acid, and salazinic acid are mostly present in lichen species, and the biological potential of these compounds to counteract oxidative stress was reported [10,11,12,13,14].

**Figure 1:**
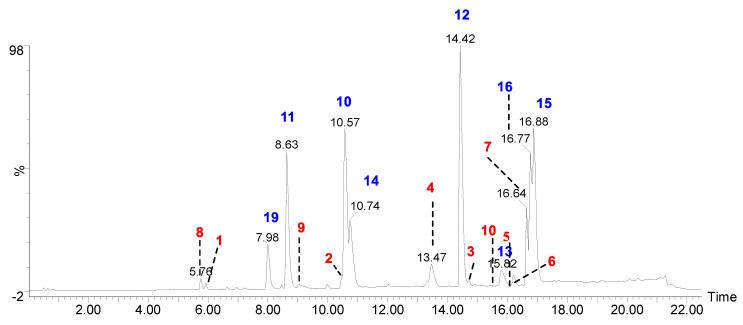
UPLC-Q-ToF-MS^e^ total ion chromatogram of acetone extract of *H. cirrhata* (blue color indicates the isolated compounds; red color indicates the MS/MS-based tentative assigned peaks (see the Supplementary Materials).

Although many contributions concerning various biological activities have been reported so far, the systematic investigations of lichen compounds for their bioactive potentials have not been thoroughly carried out. Therefore, in continuation of our efforts in the quest for the identification of active constituents from Indian lichens [15,16], we have developed the bioassays (antioxidant and antihyperglycemic) in parallel to the comprehensive analysis of metabolites from the acetone extract using an Ultra Performance Liquid Chromatography-Time of Flight Mass Spectrometric (UPLC-ESI-Q-Tof-MS/MS) method. The analysis of resultant total ion chromatogram (TIC)/full-scan MS and MS/MS spectral data allowed us to identify each metabolite through their accurate mass, including their molecular formula. In the preliminary screening, the acetone extract of *H. cirrhatum* displayed potent antioxidant and α-glucosidase inhibition activities. Subsequent activity-guided fractionation of acetone extract led to the isolation and identification of ten compounds (**11–20**) (Figure 1), which were appraised for antioxidant and antihyperglycemic activities. In order to better understand the mechanism of activity, molecular modeling studies were carried out for α-glucosidase inhibition with the most active compounds.

## 2. Results and Discussion

The species *H. cirrhata* was identified by morphological characteristics of the thallus and chemical spot tests. Aqueous potassium hydroxide (K), bleaching powder or an aqueous solution of calcium hypochlorite (C), and an aqueous solution of para-phenylenediamine (P) are the three chemical reagents used in the color spot test on the surface of the thallus (upper cortex) and medulla [17]. Details are given in the Supplementary Materials, and K+ yellow turning red suggests the presence of atranorin and chloroatranorin in the cortex, while P+ orange could be indicative of the presence of salazinic acid in the medulla.

These usual spot tests are based on the presence of lichen metabolites, but accurate analytical studies and isolation of compounds are necessary to ascertain their identification.

### Chemical Profiling, Isolation, and Structure Elucidation

Recent advances in hyphenated analytical techniques have remarkably widened their applications to the analysis of natural products and characterization of diagnostic fragments for each type of compound in the crude extracts [18,19]. Particularly, Liquid chromatography (LC) coupled to a mass spectrometer (quadrupole-time-of flight; Q-Tof) has proved its effectiveness in establishing various quality control parameters of herbal drugs, which includes species identification, authentication, and confirmation of the quality of the crude extracts in various finished products. Moreover, the advantage of its high-resolution mass accuracy facilitates analysis of known and unknown constituents by using the online and in-house database [20]. In this study, the UPLC-ESI-Q-Tof-MS/MS method was optimized to resolve metabolites in acetone extract of *H. cirrahtum* and investigated both in positive and negative ESI ionization modes to establish the total ion chromatogram (TIC). The peak assignment with proposed molecular formulas was established based on fragment ions, accurately calculated masses, and retention time. The comparison of the standards with TIC of the extracts and search in online databases (Reaxys, SciFinder, and DNP) led to the identification of 22 compounds from the acetone extract of *H. cirrhata* (Figure 1 and Table 1).

**Figure 2:**
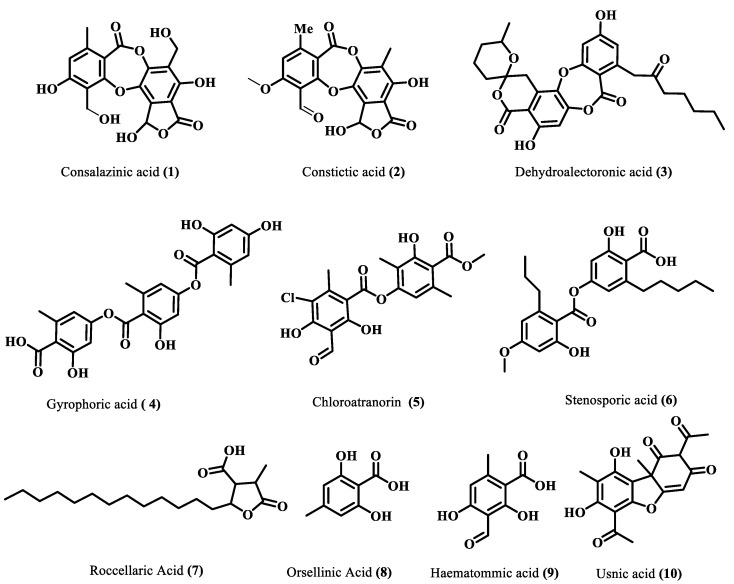
Identified compounds through LC-ESI (-)-MS/MS.

The majority of identified compounds belong to depsides, depsidones, paraconic acids, and mono phenolic compounds, concluded through mass fragmentation mechanism using CID-MS/MS and a comparison with the previous lichen metabolite mass fragmentation studies (Figure 2) [15]. The analysis of base peak ions in mass spectra analysis indicated the dominating peaks are salazinic acid (**11**), norlobaridone (**12**), atranorin (**13**), lecanoric acid (**14**), lichesterinic acid (**15**), protolichesterinic acid (**16**), which concluded with a comparison with the isolated standards and their mass fragmentation. These were taken as representative compounds for the MS/MS study to identify related compounds. In detail, peaks at (Rt) 5.92, 7.25, 8.64, 10.49, and 14.40 min were identified as depsidones, based on fragments obtained through the loss of CO_2_, CO, and CH_2_O, which were shown in Table 1 and Supplementary Materials. The peak at Rt 5.92 with a molecular 389.0509[M-H]- is 2Da more than salazinic acid and lacks one double bond equivalence (DBE). Further, fragments at *m*/*z* 371.0398, 345.0593, 327.0503, 309.0400, and 297.0398 corresponding to sequential loss of CO_2_, H_2_O, and CH_2_O, etc., indicated the presence of -CH_2_OH group instead of −CHO as in salazinic acid and were corresponding to consalazinic acid (**1**). Similarly, structures of remaining depsidones (**2** and **3**) were assigned based on their diagnostic fragmentation patterns, and detailed fragmentation studies are shown in the Supplementary Materials. Retention times at 6.63, 10.76, 13.45, 15.73, 15.80, and 16.21 were concluded as depsides based on their diagnostic peaks resulting from cleavage of the ester bond. Additionally, these data also coincide with those of their standards and the literature [15]. Fragments at *m*/*z* 195.0651 (C_10_H_11_O_4_) and 177.0182 (C_9_H_5_O_4_) from parent ion *m*/*z* 373.0923 indicate the cleavage of the ester C-O bond between two aromatic rings and were related to atranorin, which further matched with standard. Similarly, chloro-atranorin, thamnolic acid, gyrophoric acid, and stenosporic acid also showed the fragments of ester cleavage (see the Supplementary Materials). Similarly, peaks at R_t_ 16.65, 16.75, and 16.90 were assigned as the paraconic acid type compounds based on fragmentation patterns and comparison with standards and the literature (Supplementary Materials). Monophenolic compounds were identified through a comparison with our previous literature [15,16].

**Figure 3:**
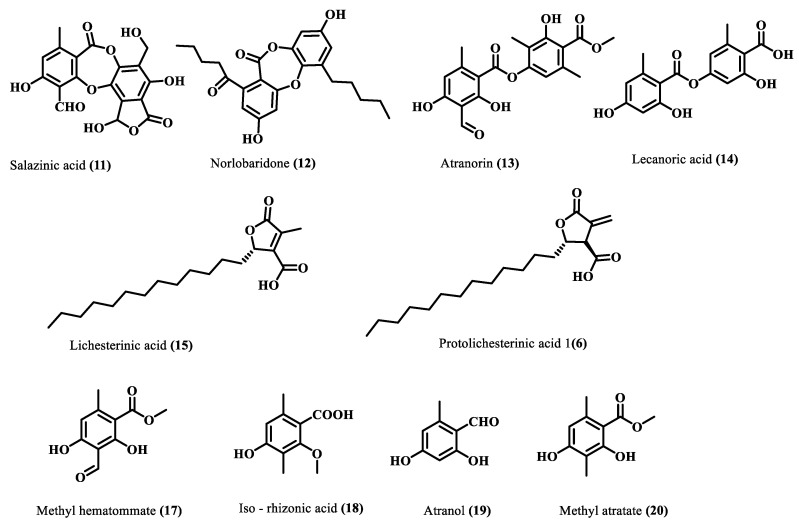
Isolated compounds from *H. cirrhata*.

Subsequently, the crude acetone extract (10 g) was chromatographed on silica gel (100–200 mesh), and the resultant fourteen fractions were subjected to a series of chromatographic separations, RP-HPLC, and preparative TLC to afford ten compounds (**11**–**20**). Comparing their physical and spectroscopic data with literature (Supplementary Materials), they were characterized as salazinic acid (**11**) [21], norlobaridone (**12**) [20], atranorin (**13**) [21], lecanoric acid (**14**) [21], lichesterinic acid (**15**) [22], protolichesterinic acid (**16**) [22], methyl hematommate (**17**) [23], iso-rhizonic acid (**18**) [24], atranol (**19**) [25], methyl atratate (**20**) [21] (Figure 3). Salazinic acid was obtained from a precipitate of the crude extract, while other compounds were purified from some chromatographic steps. The spectra and fragmentation patterns of these molecules are shown in the Supplementary Materials (Figures S7–S36).

## 3. Biological Activity

### 3.1. ABTS·+ and DPPH Radicals Scavenging Activity Analysis

**Figure 4:**
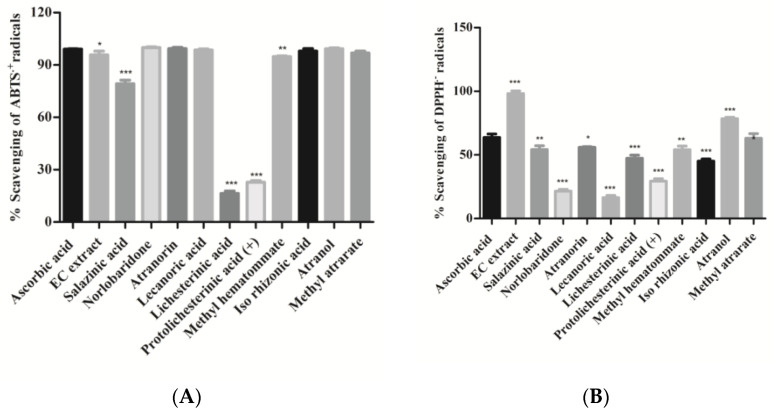
(**A**). ABTS^.^+ radical scavenging assay; (**B**). DPPH^.^-radical scavenging assay: Compounds and *H. cirrhata* extract (2 mg/mL) were incubated with ABTS^.^+ radicals. Percentage (%) scavenging was calculated using the spectroscopic method. Values were expressed as mean ± SD, *n* = 3, and calculated using one-way ANOVA followed by Dunnett’s multiple comparison tests. Degree of statistical significance was set at *p* < 0.05. Ascorbic acid vs. all compounds and extract (* *p* < 0.05, ** *p* < 0.01, and *** *p* < 0.001). Ascorbic acid was used as a standard at the same concentration.

In the present study, free radicals scavenging activity of crude extract and isolates was investigated using the standard protocols of ABTS**·**+ and DPPH assays and ascorbic acid as positive control. The amphiphilic nature of the ABTS**·**+ cation is applied to identify both the hydrophilic as well as lipophilic antioxidants present in the extract [26], whereas organic nitrogen-centered DPPH radical evaluates the reducing power of an antioxidant [27]. The percentage of free-radicals (ABTS•+ and DPPH•+) scavenging activity of isolated compounds and extract are shown in Figure 4 and Table 2. It can be observed that the majority of isolated compounds have significantly neutralized ABTS•+ radicals (more than 90%); in particular, compounds **12**, **13**, **14**, **19**, and **20** exhibited potent free radical scavenging activity (IC_50_, 0.068–0.11 µg/mL), which is comparable to that of a standard drug (ascorbic acid). In other compounds, salazinic acid (**11**) scavenged 80% ABTS radicals, while compounds **15** and **16** scavenged less ABTS radical (approx. 20% and 28%). It appears, therefore, that the presence of aromatic hydroxyl in compounds **12**, **13**, **14**, **18**, **19**, and **20** led to the improvement of their scavenging activity. On the other hand, the absence of hydroxyl groups and aromatic rings drastically reduced the scavenging capacity, as observed in compounds **15** and **16**. In the case of the DPPH-radical scavenging assay, acetone extract (AE) scavenged DPPH radicals more significantly (*p* < 0.001) than the isolates (Figure 4B). It can be observed from the results that atranol (**19**) and methylatratate (**20**) showed the highest DPPH radical scavenging capacity, which is comparable to the standard. The compounds salazinic acid (**11**), atranorin (**13**), methyl hematommate (**17**), and iso-rhizonic acid (**18**) moderately scavenged DPPH radical. Norlobaridone (**12**), lecanoric acid (**14**), and protolichesterinic acid (**16**) performed poorly at scavenging the DPPH radicals. Overall, we observed that all the compounds showed potent ABTS•+ scavenging activities, but DPPH scavenging activity was detected to be moderate in all compounds except compounds **12**, **14**, and **16** (Figure 4). The results were similar to those reported in our previous reports from *Parmotrema tinctorum* [15]. This may be predicted/explained based on the nature of the radical. ABTS•+ is a planar radical, which can be used to identify antioxidants even with low redox potentials. On the other hand, due to the steric barrier of the N• radical, they may react slowly or not when tested on DPPH radicals [28].

### 3.2. Protective Effect on Oxidative DNA Damage

**Figure 5:**
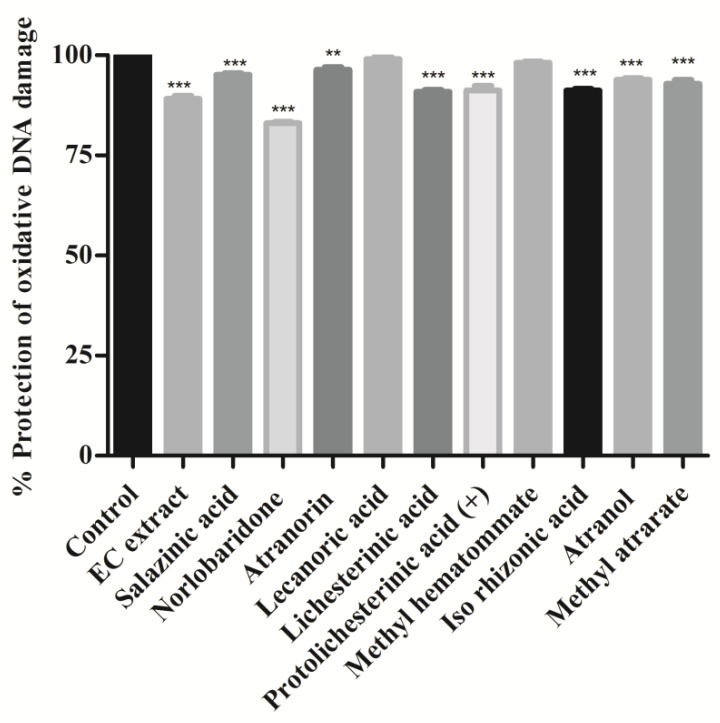
Oxidative DNA damage inhibition assay: Calf thymus DNA was mixed with compound extract (2 mg/mL) and Fenton’s reagent and incubated for 10 min at 37 °C. Values were expressed as % protection of oxidative DNA damage and indicated as mean ± SD, *n* = 3. One-way ANOVA followed by Dunnett’s multiple comparison tests was applied to compare differences within the bars. Values are significant when compared with control (** *p* < 0.01, and *** *p* < 0.001) at the same concentration.

The hydroxyl radicals of Fenton’s reagent (FR) react with nitrogenous DNA bases to produce sugar and nitrogenous base radicals. The base radicals, in turn, disrupt the sugar-phosphate skeleton of DNA, leading to strand breaks [29]. We found that the addition of most of the isolated compounds and the acetone extract with Fenton’s reagent showed a significant degree (*p* < 0.001) of protection and preserved the integrity of genomic DNA. The whole DNA (calf thymus DNA) without FR was used as a positive control for this assay and compared to the DNA damaged using Fenton’s reagent alone. As shown in Figure 5, it was noticed that all compounds showed significant protection against hydroxyl radical-induced DNA damage (*p* < 0.001, methylatratate). Norlobaridone (**12**) showed less protective than the other isolated compounds (Figure 5). The genoprotective activity of these compounds and the EC extract may be attributed to the presence of free radical scavenging potential. The DNA damage potentials of salazinic, norlobaridone, atranorin, lecanoric, and lichesterinic were reported for the first time.

### 3.3. Intestinal α-Glucosidase Activities

**Figure 6:**
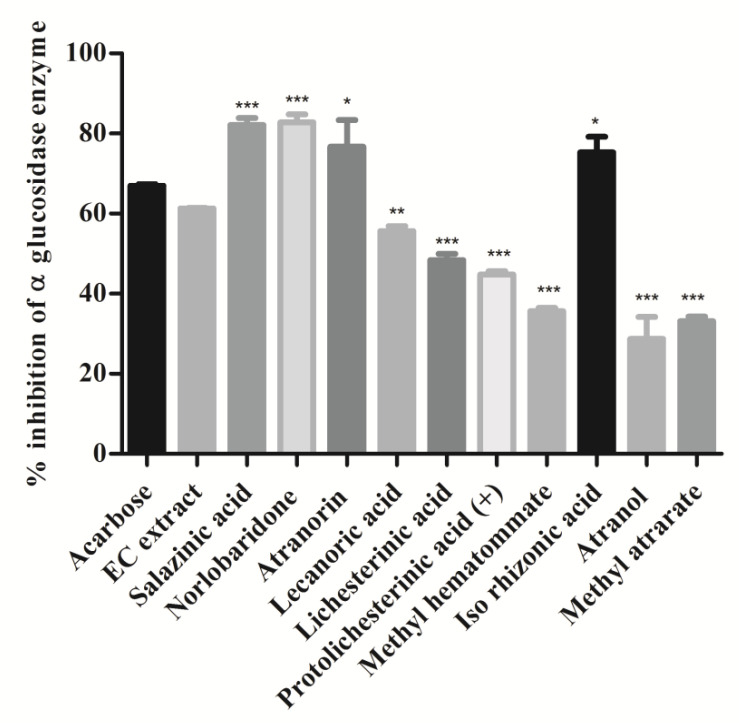
Intestinal α-glucosidase enzyme inhibition assay: Compounds and extract (2 mg/mL) were incubated with intestinal α-glucosidase enzyme and substrate (p-nitrophenyl α-D glucopyranoside). The release of 4-Nitrophenol was measured at OD 405 nm. Values were expressed as % inhibition and indicated as mean ± SD, *n* = 3. One-way ANOVA followed by Dunnett’s multiple comparison tests was applied to compare differences within the bars. Values are significant when compared with acarbose (* *p* < 0.05, ** *p* < 0.01, and *** *p* < 0.001). Acarbose used as a reference drug at the same concentration.

α-Glucosidase enzyme is a key enzyme that catalyzes the digestion of disaccharides. Inhibition of α-glucosidase in the intestine delays the digestion process and overall absorption rate of glucose into the blood [30]. A variety of small molecules with different heterocyclic cores are investigated as α-glucosidase inhibitors, whereas limited naturally occurring compounds or extracts are reported to date [31]. In this study, the α-glucosidase inhibitory activity of extract and isolates was investigated, and results are shown in Figure 6. Acarbose was taken as a positive control. As per the data shown in Figure 6, extract and isolated compounds have significantly inhibited α-glucosidase enzyme. Specifically, depsidones (salazinic acid (**11**) and norlobaridone (**12**)) were found to be more active against α-glucosidase (*p* < 0.001) with IC_50_ values of 1.62 ± 0.07 and 1.41 ± 0.01 and were more potent than the standard acarbose. Similarly, the depside, atranorin (**13**), and mono-phenolic iso-rhizonic acid (**18**) also displayed potent inhibitory activity, but less significant (*p* < 0.05) than depsidones. Lecanoric acid (**14**) (55%), paraconic acids, (lichesterinic acid (**15**) (48%) and (+)- protolichesterinic acid (**16**) (44%)) exhibited moderate α-glucosidase inhibitory activity. The other compounds, methyl hematommate (**17**) (35%), atranol (**19**) (28%), and methylatratate (**20**) (33%), moderately inhibited the α-glucosidase enzyme. Our result confirms the alpha-glucosidase inhibition of salazinic acid reported [32] and recently described from lichen Parmotrema dilatatum [33].

## 4. Molecular Modeling Studies

Many small molecules, such as acarbose, bind to the active pocket of the α-glucosidase enzyme and inhibit it. Later, investigations identified ursolic acid and oleanolic acid, which bind to the allosteric pocket and thus are useful for further understanding of α-glucosidase inhibition [34]. In the present study, the ligands were docked into an acarbose pocket. However, the dock score of both the ligands (salazinic acid = −6.288 kcal/mol; norlobaridone = −9.342 kcal/mol) showed a prominent difference compared to acarbose (−16.074 kcal/mol), especially salazinic acid. Therefore, the allosteric pocket was considered, and the FTMap-based active site prediction was made [35,36] in addition to using Sitemap analysis to show possible druggable sites of α-glucosidase. The combined overlapping of the predicted active sites from FTMap and Sitemap provided a key common binding pocket (allosteric site) formed by flexible lysine residues, histidine, and other hydrophobic amino acids. Then, the binding affinity studies of salazinic acid (**11**) and norlobaridone (**12**) employing the predicted site (Figure S38a) were performed to identify the preferential binding patterns, which displayed better affinity compared to the active pocket (salazinic acid = −7.532 kcal/mol; norlobaridone = −7.204 kcal/mol). Later, the binding free energies were calculated, and best poses were also determined.

### 4.1. Molecular Interaction Analysis of Active Pocket

The co-crystal ligand bound in the α-glucosidase pocket showed acarbose with essential polar interactions with His600, Asp327, Asp542, and Arg526, which form a stable polar cavity; while maintaining additional polar interactions with Asp203, Thr205, and Asn207 residues. The current acarbose-active site is also well networked by hydrophobic contacts with Trp441, Trp539, Ile328, Trp406, Phe450, and Ile364. Molecular interaction analysis of salazinic acid analogs (hydrazones, carbamates, and fused pyrazine) in the α-glucosidase pocket was recently studied, and preliminary findings showed the significance of polar contacts, though an in-depth in silico analysis is yet to be made [33]. Initially, the ligands were iterated using a macromodel by retaining their chirality to provide 3D poses. The protein complex with acarbose (PDB ID: 2QMJ) was chosen for the binding mode studies of the natural products. The Glide-based molecular interactions afforded low binding affinity poses and later, on subjecting to induced-fit docking (IFD) methods, delivered maximum reliable protein–ligand complexes. The α-glucosidase consists of several hydrophobic and hydrophilic residues, which enables the acarbose to make many hydrophobic interactions (Trp441, Trp539, Ile328, Trp406, Phe450 and Ile364) and H-bonds (His600, Asp327, Asp542, Arg526, Asp203, Thr205, and Asn207) (Figure S38b). The best IFD pose of salazinic acid also showed acarbose-like network with Trp406 via π-π bond and a π-cation bond with Arg526. Furthermore, the hydroxyl groups of salazinic acid depicted key H-bonds with Asp327, Asp542, Thr205 and additional H-bonds with Asp443 and Tyr605 (Figure S38c). On another aspect, the norlobaridone, unlike acarbose, comprises alkyl chains on the aromatic rings and is assumed to participate in Van der Waals interactions. In spite of alkyl chains, norlobaridone has successively established polar contacts with Gln603, Tyr605, and Asp443 (Figure S38d); additionally, both aromatic rings showed π-π network with Phe575. Later, overlapping both ligands with acarbose in the active pocket identified them as good α-glucosidase binders, which is shown in Figure S38e to know the differential binding modes. The aromatic rings of the two ligands established π-network, unlike the acarbose.

### 4.2. Allosteric Binding Studies

Both FT-Map analysis (Online-based) and Sitemap (commercial tool) derived a common α-glucosidase allosteric pocket, which was investigated and proposed as an alternative pocket for exploration [33]. Initially, the norlobaridone (**12**) in the allosteric pocket showed polar interactions with Arg283 and π-π stacking with His645; also, the allosteric pocket constituted a deeper grove, which is occupied by the alkyl chains (Figure S39a). Salazinic acid (**11**) displayed side chain H-bonds with Asp777 and Lys776; backbone H-bonds with Leu286, Ala509, and Asp777 (Figure S39b). In order to identify the preferable binding region of salazinic acid and norlobaridone in α-glucosidase enzyme, the outputs of the ligand–receptor complexes were subjected to predict the MM-GBSA binding free energies. The binding free energies indicated the preferential possession of ligands (salazinic acid = −55.41/−47.93 and norlobaridone = −60.78/−49.77 kcal/mole) in the cavity than acarbose (−25.97/−30.59 kcal/mole). Thus, in silico inhibition profile of α-glucosidase enzyme exhibited by both test compounds was greater than acarbose.

### 4.3. Molecular Dynamics Simulations

**Figure 7:**
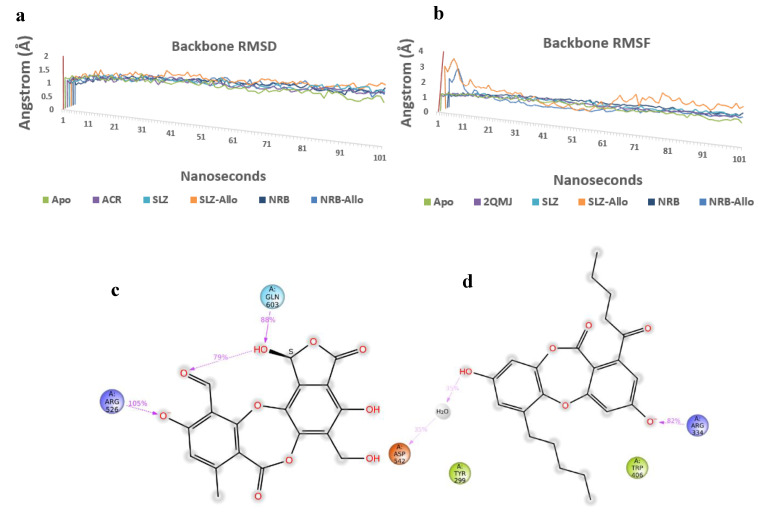

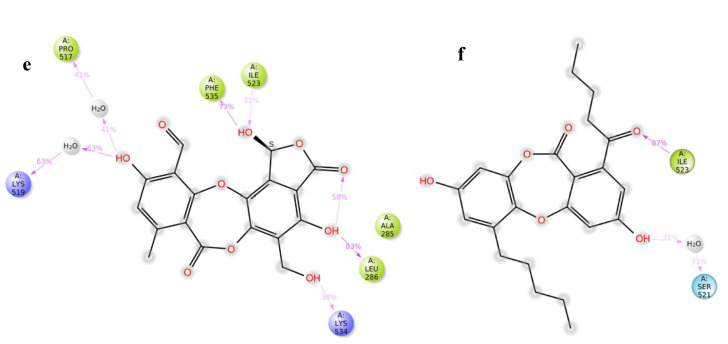
(**a**,**b**) Overlap of protein backbone RMSD and RMSF plots from 100 ns frames; (**c**,**d**) percentage interaction ofsalazinic acid and norlobaridone with catalytic residues and (**e**,**f**) percentage interaction of salazinic acid norlobaridone with allosteric residues.

The Root Mean Square Deviation (RMSD) and Root Mean Square Fluctuation (RMSF) plot analysis show good stabilization of Apo-protein, acarbose complex, salazinic acid, and norlobaridone (Figure 7a,b). The Molecular Dynamics studies investigated the deep involvement of π-π contacts between aromatic rings of natural product isolates and hydrophobic residues around acarbose binding pocket to identify the stable interactions. The selective IFD poses that show π-π contacts were initially subjected to 100 ns MD simulations, and resulting outputs were analyzed. The involvement of acarbose with key residues and resulting interactions during the simulation period displayed fundamental information about polar contacts. The residues include Asp443, Asp327, His600, and Arg526 and are found to be deeply networked through H-bonds with hydroxyl groups of acarbose during the entire 100 ns simulations, whereas Asp571 and Asp203 connect through bridged H-bond hydroxyl moieties (Figure S37). Although acarbose is surrounded by Trp539 and Tyr299, only a single H-bond is observed, and the lack of π interactions because of the lack of an aromatic system is clearly identified. Moreover, it has 22 rotatable bonds, and alicyclic nature makes them sustain within the binding pocket, which is enforced by polar H-bonds. The deeply buried Valienamine core of acarbose in the binding pocket extended maximum polar contacts, which is another factor behind its stability in the cavity. Throughout the simulations, salazinic acid furnished only two H-bonds with Arg526 (105%) and Gln603 (88%). Although it has π-π aromaticity, which played a key role in the establishment of a hydrophobic network with Trp406 and Arg526, they are identified with minimal involvement (<30%) (Figure 7c). Similarly, the Phe575, Phe450, and Tyr299 are shown as weak hydrophobic enforcers of salazinic acid (**11**). The distance between the aldehyde of the maleic anhydride core and the chiral hydroxyl group (one of the rotatable bonds) is conformationally oriented to establish intra-molecular H-bond (79%), and thus, its binding pose is constrained in the pocket (Figure 7d). Thus, the binding profile is not comparable to that of acarbose. Norlobaridone (**12**) consists of two alkyl chains on both aromatic rings. Post-trajectory analysis depicted one H-bond with Arg334 (82%) and a bridged H-bond with Asp542 (35%). As IFD studies show, significant π-π aromaticity establishes hydrophobic contacts with key residues, but the resulting π-π network was destabilized in the simulation. However, additional hydrophobic contacts with Try299, Trp406, Phe450, Phe575, Ala576, Ile364, and Ile328 are observed. The presence of alkyl chains resulted in furnishing Vander Waals forces with hydrophobic residues in the α-glucosidase binding pocket, and interestingly, the profile is comparable to that of acarbose. To know the stability of the alkyl chain, whether it may be stably accommodated in the allosteric pocket, and the behavior of salazinic acid in the allosteric pocket, we performed MD simulations for about 100 ns. The salazinic acid (**11**) and norlobaridone (**12**) in initial binding studies show the maximum number of essential interactions with both active and allosteric residues. However, the subjection of long-term simulation clearly showed favorable residues that are involved in holding the salazinic acid and norlobaridone at the allosteric pocket.

The simulation clearly shows that the alkyl chain of norlobaridone is essential and occupies the prerogative allosteric grove by coordinating with Ile523 (87%) through the H-bond in the simulations (Figure 7e). However, the interaction profile is lacking compared to the profile on the active site, indicating norlobaridone’s preference for the active site. Salazinic acid, on the other hand, established several hydrophilic contacts with listed residues in the induced-fit mechanisms along with a water bridge with Lys519 (63%) and a H-bond with Lys534 (38%) (Figure 7f). We also found that the furanone ring shows an intra-molecular H-bond with an adjacent hydroxyl group in both simulations that might restrain its entry into both active and allosteric pockets (Figure 7c,e). Moreover, the allosteric site provided a wider interacting network for salazinic acid, suggesting the mechanism of action of salazinic acid might be allosteric binding. On the other hand, norlobaridone has a very similar profile for both the sites; however, based on the dock score, the active site may be preferred. The interaction network of active and allosteric pockets involved with salazinic acid (**11**) and norlobaridone (**12**) are shown in Figure S40.

## 5. Materials and Methods

### 5.1. General

The ^1^H and ^13^C NMR spectra of the pure compounds were recorded on a Bruker FT-300/400 MHz spectrometer using TMS as an internal standard. The chemical shifts were expressed as δ values in parts per million (ppm), and the coupling constants (J) were given in Hertz (Hz). HRESIMS data were acquired on XevoTM G2 XS-ESI-QTof mass spectrometer (Waters Corp., Manchester, UK). Column chromatography was carried out using silica gel 60–120, 100–200 mesh (Qingdao Marine Chemical, Qingdao, China), and precoated silica gel plates (Merck, 60 F_254,_ Billerica, MA, USA) were used for thin layer chromatography analysis. Purification of the fractions was achieved using a Gilson preparative-HPLC (Middleton, WI, USA) instrument, which is equipped with 321 binary pump, GX-281 liquid handler, and UV-155 detector with × Select HSS T3 column (250 × 100 mm, 5 µm) (Waters Corp., Wexford, Ireland) as stationary phase using Trilution LC v2.1 platform. The required HPLC grade solvents (acetonitrile) were purchased from M/S Merck (Darmstadt, Germany), formic acid (Optima™Mass spec grade) from Thermo Fisher Scientific (Geel, Belgium), and water for the mobile phase was purified by using a Milli-Q system (Millipore system, MA, USA).

### 5.2. Instrumental UPLC Conditions

The instrumental conditions were set up based on our recent method with suitable modifications [15]. The liquid chromatographic analysis was performed on the Xevo TQ-S micro-MS system coupled with Acquity H-class UPLC (Waters, Milford, MA, USA), equipped with auto sampler, degasser, binary pump, and PDA detector. The separation was achieved by using ACQUITY UPLC CSH Phenyl-Hexyl column (100 × 2.1 mm id., 1.7 μm particle size) (Waters, Milford, MA, USA) with a gradient mode elution based on a mixture of water of (A) and acetonitrile (B) containing 0.1% formic acid at a flow rate of 0.4 mL/min. The column temperature was set at 40 °C throughout the experiment. The following gradient: 0 min, 5% B; 3.00 min, 20% B; 5.00 min, 35% B; 7.50 min, 50% B; 10.00 min, 70% B; 12.50 min, 95% B; B; 17.00 min 95% B; 21.00 min 5% B with a total run time of 22 min. The mass spectrometric analysis was performed with Xevo G2-XS Q-Tof mass spectrometer (Waters, Manchester, UK) equipped with an Electrospray ionization (ESI) source with the following parameters: capillary voltage, 2.0 KV; sample cone, 40 V; source temperature, 120 °C; desolvation temperature 350 °C; cone gas flow rate 50 L/h; desolvation gas (N2) flow rate 850 L/h, Argon as CID gas for MS/MS experiments. The sample collision energy was set at 15–45 eV. All the operations, acquisition, and data analysis were controlled by Waters Mass lynx Software v. 4.1. Acquiring data in this manner provided information on intact precursor ions as well as fragment ions. All analyses were performed using lock spray, which ensured accuracy and reproducibility. Leucine-Enkephalin (5 ng/mL) was used as lock mass, generating a reference ion in negative mode at *m*/*z* 554.2615 introduced by a lock spray at 10 μL/min for accurate mass acquisition.

### 5.3. Lichen Collection and Identification

The lichen, *H. cirrhata*, was collected from Bichpuri Range, Bijrani Zone of Corbett National Park, alt. N 29_26040” E79_04006 (1283 m) in May 2019. Botanical details of the thallus and apothecia of lichen were checked under light lens Magnüs MS 24/13 and ZEISS Axiostar. Spot tests for color response were carried out with the usual reagents used in lichenology C (Calcium hypochlorite), PD (p-phenylene diamine), and K (potassium hydroxide). The thin layer chromatography (TLC) chemical profile was realized with the solvent system ‘A’, according to White and James’s methods (1985) and referred to recent literature [37]. The collected voucher specimens of lichen species were placed in the Lichen Herbarium of Yogi Vemana University, Kadapa, Andhra Pradesh. The details are provided in the Supplementary Materials, Figures S1–S3.

### 5.4. Extraction and Isolation

Dried lichen *H. cirrhata* (380 g) was powdered and extracted with acetone at room temperature for 48 h. The solvent was evaporated under reduced pressure using a rotary evaporator and 10 g of crude extract was obtained. The crude extract was washed with acetone to obtain the solid, which was further washed repeatedly with acetone and compound **11** (10.25 mg) was obtained in pure. The remaining crude extract was subjected to silica gel column chromatography (60–120 mesh) and eluted with n-hexane/acetone by increasing the polarities to obtain 14 fractions. All these fractions were analyzed by TLC (Silica gel 60F254, toluene: 1,4-dioxane: acetic acid (84:8:8), and the fractions with similar TLC patterns were combined together into three major fractions (F1, F2, and F3). Fraction F1 was subjected to column chromatography on silica gel (100–200 mesh), eluted using n-hexane-acetone (95:05) to yield compound **12** (70 mg), and compounds **13** (50.23 mg) and **14** (12.58 mg) were eluted with n-hexane-acetone (9:91). Fraction F2 was subjected to column chromatography on silica gel (100–200 mesh), eluted using n-hexane-acetone (85:15) to obtain compound **15** (50.0 mg) and further increasing the polarity to 82:18 (n-hexane-acetone) to yield compounds **16** (350.54 mg) and **17** (7.89 mg). Fraction F3 was subjected to column chromatography on silica gel (100–200 mesh) eluted using n-hexane-acetone (75:25) to yield compound **18** (5.98 mg), and compounds **19** (3.56 mg) and **20** (170.43 mg) were obtained at n-hexane-acetone (70:30).

### 5.5. In Vitro Antioxidant and Antihyperglycemic Assay

#### 5.5.1. DPPH Radical Scavenging Activity

Assay for the scavenging of stable free radical 1,1-Diphenyl-2-picrylhydrazyl (DPPH) was carried out as described earlier [38]. In brief, 100 µg of acetone extract (AE) and test compounds (**11**–**20**) made in DMSO were added to a reaction mixture containing 125 μL of 0.1 M tris-HCl buffer (pH 7.4) and 125 μL of 0.5 mM DPPH solution. The reaction mixture was stirred in 96-well plates on a dancing shaker and read at 517 nm spectrophotometrically (SpectraMax^®^ plus384, Molecular Devices Corporation, Sunnyvale, CA, USA). Ascorbic acid (50 µL of 2mg/mL) served as standard. Results were expressed as % scavenging and calculated by using the formula (Ac − At)/100*Ac, where Ac was the absorbance of the control and At was the absorbance of the test sample.

#### 5.5.2. ABTS Radical Scavenging Activity

For determining ABTS (2,2′-azino-bis(3-ethylbenzothiazoline-6-sulphonic acid) radical scavenging activity, we mixed 20 µg of acetone extract (AE) and 20 µg of test compounds (**11**–**20**) with ABTS+·solution made in phosphate buffer in a 96-well microplate [39]. The loss of color of the ABTS solution was measured in a spectrophotometer at 734 nm (SpectraMax^®^ plus384, Molecular Devices Corporation, Sunnyvale, CA, USA). The percentage of oxidation inhibition by the test materials was determined as described in the text.

#### 5.5.3. Free Radical Induced DNA Damage

The protective effect of acetone extract (EC) and compounds (**11**–**20**) on oxidative DNA damage was evaluated as per the previous method [40]. Namely, 2 µL calf-thymus DNA mixed with 5 µL 39 mM Tris buffer (pH 7.4) and 5 µL (10 µg) acetone extract and compounds (**11**–**20**) (10 µg of 2 mg/mL solution dissolved in DMSO) mixture was incubated at room temperature for 20 min. Calf thymus DNA was used as a positive control. The reaction was initiated by adding 5 µL FeCl_3_ (500 µM) and 10 µL H_2_O_2_ (0.8 M) and incubating for 10 min at 37 °C. After the incubation period, 3 µL DNA loading dye was added to stop the reaction. Then, the mixture was exposed to 0.8% agarose gel electrophoresis in TAE (40 mM Tris, 20 mM acetic acid, and 0.5 M EDTA) buffer (pH 7.2) and 3 µL of ethidium bromide was added to stain DNA bands. Images were observed and captured under trans-illuminating UV (Bio-Rad Chemi DocTM XRS with Image LabTM Software, ver. 6.0.1, build34, standard edition, 2017), and ImageJ software (ver. 1.4.3.67, Broken Symmetry Software, Scottsdale, AZ, USA)was used to measure the band intensity of DNA.

#### 5.5.4. Intestinal α-Glucosidase Inhibition

The activity of rat intestinal α-glucosidase enzyme inhibition assay of acetone extract and compounds (**11**–**20**) was performed as per the previous method [38]. Namely, 20 µL of test samples (Compound 11 to 20) (2 mg/mL dissolved in DMSO) were incubated with 50 µL of crude intestinal α-glucosidase for 5 min and then again for 5 min with 50 µL of substrate 5 mM p-nitrophenyl-α-D-glucopyranoside. Acarbose was taken as a standard. Percent enzyme inhibition was calculated as (Ac − At/Ac) * 100. ‘Ac’ and ‘At’ represent the absorbance of the control and test samples, respectively. Crude rat intestinal α-glucosidase enzyme was pre-incubated with extract in phosphate buffer saline for 5 min and then reacted with substrate p-nitrophenyl-α-D-glucopyranoside for 5 min. Release of p-nitrophenyl due to action of α-glucosidase enzyme was recorded spectrophotometrically at 405 nm. The effect of isolates (**11**–**20**) on enzyme activity was compared with the acarbose control (2 mg/mL), where the enzyme was reacted with substrate only. The activity was expressed and calculated as follows: (Ac − At)/100*Ac, where Ac was the absorbance of the control and At was the absorbance of the test sample.

### 5.6. Molecular Modeling Studies

The three-dimensional X-ray diffraction structure of α-glucosidase complexed with acarbose with resolution 1.90 Å retrieved from a protein data bank with accession number 2QMJ (https://www.rcsb.org/structure/2qmj, accessed on 20 September 2023) was used for the current study [41]. It consists of chains A, B, and C (acarbose) prepared by removing water, ions, and salts, optimizing bond orders and bond lengths, and energy minimization using a protein preparation wizard. The receptor grid by selecting acarbose (pocket1) was generated using Glide to check the binding pattern of the natural product isolates. In order to predict the allosteric pocket of α-glucosidase, the FT-Map online server was utilized, and outputs were reconfirmed by using Sitemap-based active site prediction analysis. From the comparative maps from both predictive studies, the allosteric site with a good site score (>1) was employed as pocket2 to generate a grid. The ligands were sketched using the ChemDraw tool, imported into the panel, and subjected to ligand preparation. The protein–ligand docking was carried out using the Glide tool by employing different docking methods, initially, standard precision followed by extra precision methods. Further, the MM-GBSA study was also carried out to calculate the binding free energies using Prime with default parameters. Later, induced-fit standard protocol by defining the centroid of length 20 Å was carried out. The active pocket was defined by acarbose, while the allosteric pocket was defined by specifying a few residues (Leu286, Arg283, and Asp777). Then XP Glide redocking was enabled, and the IFD was run, retaining the other default parameters to generate possible poses. The poses with good binding patterns, dock scores, from IFD outputs were handpicked for further studies.

### 5.7. Molecular Dynamics Protocol

The MD simulation module of Desmond was employed to investigate the steadiness of salazinic acid and norlobaridone in both active (pocket1) and allosteric pockets of α-glucosidase. The selected protein–ligand complexes were prepared for dynamics by setting up using a single point charge (SPC) model, which was charged with 0.15 M of sodium chloride and subjected to energy minimization with default parameters (OPLS_3e force field). The obtained solvate model was further utilized as input for preset Desmond MD simulations protocol fixed with the isothermal–isobaric ensemble (NPT ensemble), constant 1.01325 bar pressure, and 300 K temperature for about 100 ns. During the simulation, a checkpoint file at each 1 ns frame was enabled to afford the 100 trajectory files. The simulation interaction diagrams and event analysis were further analyzed from output files of the trajectories to analyze the essential parameters, including RMSD and RMSF. Three dimensional poses derived from trajectories were exported to the Maestro panel to evaluate the binding modes [42].

## 6. Conclusions

Overall, the present study summarizes the isolation of bioactive constituents from the *H. cirrhata* and further applicability of ESI-Q-TOF-MS/MS for the rapid identification of metabolites from crude extracts based on MS/MS fragmentation patterns. This approach was useful for concluding the identification of two dozen lichen substances, adding metabolites to the list of described compounds from this macrolichen, common in India. The isolation and identification of a dozen of these compounds allowed us to check their antioxidant activity. Most phenols supported the DPPH and ABTS scavenging activities of the acetone extract. In addition to these antioxidant assays, anti-diabetic potential was assessed through α-glucosidase inhibition, which confirmed the recognized activity of salazinic acid [16,21,33] and revealed a comparable activity for norlobaridone, a depsidone with alkyl chains. An intensive molecular modeling study of these two depsidones as α-glucosidase binders was undertaken by employing both active and allosteric pockets. A good fit in the active pocket could be described for both of them having a partial overlap with acarbose. Further simulation studies showed the necessity of norlobaridone alkyl chain occupied in the deeper grove of the allosteric pocket. With the superior binding free energies contributed by both compounds, acarbose might have backed the α-glucosidase inhibitory action. As for salazinic acid, significant inhibition of α-glucosidase enzyme led very recently to medicinal chemistry approaches to identify a few more promising α-glucosidase inhibitors [30]. A similar work should be undertaken with norlobaridone.

## Figures and Tables

**Table 1:** LC-QToF-MS^E^ data of compounds from acetone extract of *Hypotrachyna cirrhata*.

S.No	Compound Name	RT (min)	Formula	Mass (*m*/*z*) Calculated	Mass (*m*/*z*) Observed	Error (Δ ppm)	Fragmentation Pattern
01	Orsellinic acid ^#^ (**8**)	5.75	C_8_H_7_O_4_	167.0344	167.0341	−1.8	123.0440 (C_7_H_7_O_2_)
02	Consalazinic acid (**11**) ^#^	5.92	C_18_H_13_O_10_	389.0509	389.0506	−0.8	371.0398(C_18_H_11_O_9_), 345.0593(C_17_H_13_O_8_) 327.0503(C_17_H_11_O_7_), 309.0400(C_17_H_9_O_6_) 297.0398(C_16_H_9_O_6_), 281.0449(C_16_H_9_O_5)_ 265.0501(C_16_H_9_O_4_), 253.0500(C_15_H_9_O_4_) 227.0344(C_15_H_9_O_4_), 225.0554(C_14_H_9_O_3_) 209.0603(C_14_H_9_O_2_), 197.0594 (C_13_H_9_O_2_) 171.0409 (C_11_H_7_O_2_).
03	ND	6.63	C_10_H_9_O_7_	241.0348	241.0342	−2.5	223.0234(C_10_H_7_O_6_), 209.0082 (C_9_H_5_O_6_) 190.9971 (C_9_H_3_O_5_), 165.0185 (C_8_H_5_O_4_).
04	Thamnolic acid ^#^	6.94	C_19_H_15_O_11_	419.0614	419.0607	−1.7	401.0466 (C_19_H_13_O_10_), 389.0490 (C_18_H_13_O_10_) 375.0553 (C_18_H_15_O_9_), 327.0514 (C_17_H_11_O_7_) 313.0349 (C_16_H_9_O_7_), 297.0410 (C_16_H_9_O_6_) 269.0433 (C_15_H_9_O_5_), 253.0497 (C_15_H_9_O_4_) 241.0588 (C_14_H_9_O_4_), 225.0558 (C_14_H_9_O_3_) 209.0472 (C_10_H_9_O_5_), 195.0453 (C_13_H_7_O_2)_, 167.0440 (C_12_H_7_O_1_).
05	Connorstictic acid ^#^	7.25	C_18_H_13_O_9_	373.0560	373.0553	−1.9	343.0449 (C_17_H_11_O_8_) 329.0657 (C_17_H_13_O_7_), 255.0655 (C_15_H_11_O_4_), 227.0655 (C_14_H_11_O_3_), 212.0486 (C_13_H_8_O_3_), 203.0729 (C_12_H_11_O_3_), 199.0709 (C_13_H_11_O_2_), 161.0638(C_10_H_9_O_2_), 137.0609 (C_8_H_9_O_2_).
06	Atranol * (**19**)	8.00	C_8_H_7_O_3_	151.0395	151.0392	−2.0	123.0441 (C_7_H_7_O_2_), 105.0339 (C_7_H_5_O).
07	Unidentified	8.47	C_20_H_17_O_11_	433.0771	433.0771	0.0	401.0508 (C_19_H_13_O_10_), 357.0617 (C_18_H_13_O_8_), 339.0524 (C_18_H_11_O_7_), 297.0406 (C_16_H_9_O_6_), 281.0456 (C_16_H_9_O_5_), 253.0512 (C_15_H_9_O_4_), 209.0424 (C_10_H_9_O_5_), 179.0328 (C_9_H_7_O_4_), 151.0050 (C_7_H_3_O_4_).
08	Salazinic acid * (**11**)	8.64	C_18_H_11_O_10_	387.0352	387.0352	0.0	343.0459 (C_17_H_11_O_8_), 325.0355 (C_13_H_9_O_10_) 313.0353 (C_16_H_9_O_7_), 243.0295 (C_13_H_7_O_5_) 227.0344 (C_13_H_7_O_4_), 225.0552 (C_14_H_9_O_3_) 213.0551 (C_13_H_9_O_3_), 151.0394 (C_8_H_7_O_3_) 121.0287 (C_7_H_5_O_2_).
09	Haematommic acid ^#^ (**9**)	9.06	C_9_H_7_O_5_	195.0293	195.0290	−1.5	151.0391 (C_8_H_7_O_3_), 123.0442 (C_7_H_7_O_2_).
10	Constictic acid ^#^ (**2**)	10.49	C_19_H_13_O_10_	401.0509	401.0524	3.7	357.0619 (C_18_H_13_O_8_), 313.0722 (C_17_H_13_O_6_), 253.0509 (C_15_H_9_O_4_), 243.0304 (C_13_H_7_O_5_), 227.0350 (C_13_H_7_O_4_), 225.0556 (C_14_H_9_O_3_).
11	Methyl atratate * (**20**)	10.57	C_10_H_11_O_4_	195.0657	195.0658	0.5	181.0500 (C_9_H_9_O_4_), 163.0394 (C_9_H_7_O_3_), 137.0602 (C_8_H_9_O_2_), 119.0496 (C_8_H_7_O).
12	Lecanoric acid * (**14**)	10.76	C_16_H_13_O_7_	317.0661	317.0663	0.6	167.0338 (C_8_H_7_O_4_), 149.0233 (C_8_H_5_O_3_) 123.0440 (C_7_H_7_O_2_), 105.0334 (C_7_H_5_O).
13	Gyrophoric acid ^#^ (**4**)	13.45	C_24_H_19_O_10_	467.0978	467.0978	0.0	317.0663 (C_16_H_13_O_7_), 167.0340 (C_8_H_7_O_4_) 149.0237 (C_8_H_5_O_3_), 123.0443 (C_7_H_7_O_2_).
14	Norlobaridone * (**12**)	14.40	C_23_H_25_O_6_	397.1651	397.1655	1.0	353.1755 (C_22_H_25_O_4_), 329.1755 (C_20_H_25_O_4_) 313.1076 (C_18_H_17_O_5_), 296.1050 (C_18_H_16_O_4_) 272.1048 (C_16_H_16_O_4_), 201.0546 (C_12_H_9_O_3_) 188.0468 (C_11_H_8_O_3_), 159.0441 (C_10_H_7_O_2_).
15	Dehydroalectoronic acid ^#^ (**3**)	14.71	C_28_H_29_O_9_	509.1812	509.1811	−0.2	491.1705 (C_28_H_27_O_8_), 465.1921 (C_27_H_29_O_7_) 447.1801 (C_27_H_27_O_6_), 423.1574 (C_28_H_23_O_4_) 405.1404 (C_24_H_21_O_6_), 381.1403 (C_22_H_21_O_6_) 367.1207 (C_21_H_19_O_6_), 247.0965 (C_14_H_15_O_4_), 218.0951 (C_13_H_14_O_3_).
16	Usnic acid ^#^ (**10**)	15.73	C_18_H_15_O_7_	343.0818	343.0806	−3.5	328.0593 (C_17_H_12_O_7_), 313.0359 (C_16_H_9_O_7_) 259.0595 (C_14_H_11_O_5_), 231.0652 (C_13_H_11_O_4_) 215.0350 (C_12_H_7_O_4_), 177.0180 (C_9_H_5_O_4_).
17	Atranorin * (**13**)	15.80	C_19_H_17_O_8_	373.0923	373.0935	3.2	195.0651 (C_10_H_11_O_4_), 177.0182 (C_9_H_5_O_4_), 163.0390 (C_9_H_7_O_3_), 133.0285 (C_8_H_5_O_2_).
18	Chloroatranorin ^#^ (**5**)	16.21	C_19_H_16_O_8_Cl	407.0556	407.0540	−3.9	223.0970 (C_12_H_15_O_4_), 210.9796 (C_9_H_4_O_4_Cl), 205.0863 (C_12_H_13_O_3_), 195.0652 (C_10_H_11_O_4_) 163.0392 (C_9_H_7_O_3_), 138.9950 (C_7_H_4_O_1_Cl).
19	Stenosporic acid ^#^ (**6**)	16.23	C_23_H_27_O_7_	415.1757	415.1758	0.2	223.0969 (C_12_H_15_O_4_), 205.0864 (C_12_H_13_O_3_) 179.1072 (C_11_H_15_O_2_), 163.0393 (C_9_H_7_O_3_).
20	Roccellaric acid ^#^ (**7**)	16.65	C_19_H_33_O_4_	325.2379	325.2382	0.9	281.2481 (C_18_H_33_O_2_).
21	Protolichesterinic acid * (**16**)	16.75	C_19_H_31_O_4_	323.2222	323.2224	−1.5	279.2323 (C_18_H_31_O_2_).
22	Lichesterinic acid * (**15**)	16.90	C_19_H_31_O_4_	323.2222	323.2217	0.6	279.2322(C_18_H_31_O_2_).

ND = Not determined, * = Isolated compounds, # = tentatively assigned new compound concluded from MS/MS and comparison with in-house standards.

**Table 2:** Antioxidant and antihyperglycemic activities of extract and isolates from *H. cirrhata*.

Compound Name (2 mg/mL)	ABTS % Inhibition (IC_50_) µg/mL	DPPH % Inhibition (IC_50_) µg/mL	Intestinal α-Glucosidase % Inhibition (IC_50_) µg/mL	DNA Damage Assay (% Protection)
HC extract	95.79 ± 2.13	98.23 ± 2.02	61.23 ± 0.46	89.63 ± 0.89
Salazinic acid (**11**)	79.28 ± 2.07 (2.11 ± 0.04)	54.20 ± 3.03	82.10 ± 1.76 (1.62 ± 0.07)	95.44 ± 0.31
Norlobaridone (**12**)	99.95 ± 0.39 (0.59 ± 0.02)	21.57 ± 1.32	82.80 ± 1.96 (1.41 ± 0.01)	82.80 ± 9.93
Atranorin (**13**)	99.99 ± 0.51 (0.068 ± 0.02)	55.91 ± 0.46	76.69 ± 3.6 (2.07 ± 0.01)	96.87 ± 4.30
Lecanoric acid (**14**)	98.55 ± 0.06 (0.32 ± 0.02)	16.45 ± 1.71	55.61 ± 1.27	99.49 ± 0.56
Lichesterinic acid (**15**)	16.37 ± 1.35	47.31 ± 2.49	48.40 ± 1.56	90.55 ± 2.47
Protolichesterinic acid (**16**)	22.88 ± 0.90	29.45 ± 1.87	44.79 ± 0.78	90.45 ± 0.02
Methyl hematommate (**17**)	94.70 ± 0.48 (0.53 ± 0.03	54.03 ± 2.96	35.64 ± 0.78	98.39 ± 0.11
Iso- rhizonic acid (**18**)	97.96 ± 1.41 (16.37 ± 0.06)	45.05 ± 1.79	75.31 ± 3.92 (1.99 ± 0.03)	91.48 ± 0.30
Atranol (**19**)	99.23 ± 0.51 (0.11 ± 0.07)	78.46 ± 1.12 (1.73 ± 0.02)	28.71 ± 0.54	94.16 ± 2.45
Methylatratate (**20**)	96.87 ± 1.02 (1.44 ± 0.05)	63.02 ± 3.66 (3.02 ± 0.07)	33.14 ± 1.17	92.15 ± 0.35
Ascorbic acid (2 mg/mL)	99.05 ± 0.62 (0.079 ± 0.01)	83.68 ± 2.72 (1.59 ± 0.05)	-	-
Acarbose (2 mg/mL)	-	-	67.40 ± 3.08 (2.58 ± 0.85)	-

## Data Availability

The data presented in this study are available on request from the corresponding authors.

## Supplementary Materials

The following supporting information can be downloaded at: https://www.mdpi.com/article/10.3390/molecules28237840/s1. Physicochemical data of isolated compounds. Figure S1: Authenticate *H*. *cirrhata*. Figure S2: The spot tests for identification of *H. cirrhata*. Figure S3: The HPTLC profiles for *H. cirrhata* extract. Figure S4: UPLC-PDA chromatogram of acetone extract of *H. cirrhata*. Figure S5: Total Ion Chromatogram (TIC) of Isolated and identified ms/ms compounds Figures S6–S36: Spectral data (HRESIMS, MS/MS spectra and fragmentation pattern, ^1^H and ^13^C NMR spectra) of compounds **11**–**20**, Figure S37–S40: molecular docking studies. Table S1: Antihyperglycemic and antioxidant activities of isolates from *H. cirrhata*.
