# Supplementary material for: Salazinic Acid and Norlobaridone from the Lichen Hypotrachyna cirrhata: Antioxidant Activity, α-Glucosidase Inhibitory and Molecular Docking Studies
Source: Molecules. 2023 Nov 29;28(23):7840. doi: 10.3390/molecules28237840 (PMC10708527; doi:10.3390/molecules28237840)
Supplement: Supplementary file 1 [file molecules-28-07840-s001.zip › molecules-2636794-supplementary.pdf]

Physicochemical data of isolated compounds:

Salazinicacid (11): Light brown colour solid; ^{1}H NMR (500MHz, DMSO-d$_6$): δ 10.25 (1H, s), 6.87 (2H, s), 4.59 (2H, s), 2.44 (3H, s); ^{13}C NMR (100MHz, DMSO-d$_6$): δ 192.72, 165.93, 164.00, 163.56, 160.30, 152.85, 152.29, 148.11, 138.03, 137.29, 123.41, 117.41, 111.93, 110.64, 109.68, 95.07, 52.68, 21.43 ppm. HRESI-MS: *m/z* 387.0357 [M-H]$^{-}$(calcd. for $C_{18}H_{11}O_{10}$, 387.0352).

Norlobaridone (12): White amorphous powder; ^{1}H NMR (500MHz, DMSO-d$_6$): δ 9.83 (1H, brs), 6.78 (1H, d, J = 2.28 Hz), 6.75 (1H, d, J = 2.13 Hz), 6.54 (1H, d, J = 2.89 Hz),6.51 (1H, d, J = 2.74 Hz), 2.75 (2H, t, J = 7.32 Hz), 2.62 (2H,t, J = 7.62 Hz), 1.56-1.49 (4H,m), 1.35-1.28 (6H,m), 0.88-0.85 (6H, m).^{13}C NMR (100 MHz,DMSO-d$_6$): δ 203.39, 163.14, 162.84, 162.53, 154.90, 148.88, 144.01, 140.72, 136.10, 113.19, 112.05, 109.56, 107.32, 105.23, 41.11, 31.07, 29.96, 29.08, 25.54, 21.94, 21.47, 13.89, 13.79 ppm. HRESI-MS: *m/z* 397.1647 [M-H]$^{-}$(calcd for $C_{23}H_{25}O_6$, 397.1651).

Atranorin (13): Light yellow powder; ^{1}H NMR (500 MHz, CDCl$_3$): δ 12.55 (1H, s), 12.49 (1H, s), 11.94 (1H, s) 10.35 (1H, s), 6.52 (1H, s), 6.40 (1H, s), 3.98 (3H, s), 2.70 (3H, s), 2.54 (3H, s), 2.09 (3H, s); ^{13}C NMR (100 MHz, CDCl$_3$): δ 193.79, 172.16, 169.65, 169.03, 167.44, 162.83, 152.39, 151.95, 139.83, 116.74, 115.99, 112.81, 110.23, 108.51, 102.81, 52.30, 25.54, 23.98, 9.33 ppm. HRESI-MS: *m/z* 373.0927 [M-H]$^{-}$(calcd for $C_{19}H_{17}O_8$, 373.0923).

Lecanoric acid (14): Isolated as a white amorphous powder; ^{1}H NMR (500 MHz DMSO-d$_6$):δ10.35 (1H, s), 10.08 (1H, s), 6.59 (2H, d, J = 3.2 Hz), 6.23 (2H, s), 2.36 (3H, s), 2.35 (3H, s); ^{13}C NMR (100 MHz, DMSO-d$_6$): δ 170.61, 167.16, 161.10, 160.10, 158.74, 152.20, 140.35, 139.45, 116.79, 114.72, 109.88, 108.32, 107.36, 100.49, 21.35, 20.95 ppm. HRESI-MS: *m/z* 317.0667 [M-H]$^{-}$(calcd for $C_{16}H_{13}O_7$, 317.0661).

(+) -Lichesterinic acid (15): White amorphous powder; ^{1}H NMR (400 MHz, Acetone-d$_6$):δ 5.16 -5.13 (1H, m), 2.13 (3H, d, J = 2.06 Hz),1.65 – 1.59 (1H, m), 1.39-1.28 (22H, m), 0.88 (3H, t, J = 6.63, 13.73 Hz); ^{13}C NMR (100 MHz, Acetone-d$_6$): δ 174.21, 164.61, 150.03, 138.45, 82.83, 34.27, 33.62, 26.40, 24.31, 15.33, 11.81 ppm. HRESI-MS: *m/z* 323.2221[M-H]$^{-}$(calcd for $C_{19}H_{31}O_4$, 323.2222).

Protolichesterinic acid (16): White amorphous powder; ^{1}H NMR (400 MHz, Acetone-d$_6$): δ 6.27 (1H, d, J = 3.11 Hz), 5.98 (1H, d, J = 2.56 Hz), 4.78 (1H, q), 3.80 (1H, q), 1.80– 1.74 (2H, m), 1.46-1.29 (22H, m), 0.88 (3H, t, J = 6.78, 13.75 Hz); ^{13}C NMR (100 MHz, Acetone-d$_6$): δ171.95,169.64,136.25,125.26, 80.87, 51.23, 37.19, 33.62, 26.61, 24.31, 15.33 ppm. HRESI-MS: *m/z* 323.2223 [M-H]$^{-}$(calcd for $C_{19}H_{31}O_4$, 323.2222).

Methyl hematommate (17): White amorphous powder; ^{1}H NMR (400 MHz, CD_3COCD_3):δ11.98 (1H, s), 6.33 (1H, s),3.91(3H, s), 2.41 (3H, s),2.30(3H, s);^{13}C NMR (100 MHz, CD_3COCD_3):δ174.46, 165.05, 161.85, 141.53, 112.44, 110.45, 105.87, 53.10, 25.18, 9.05ppm.HRESI-MS:*m/z*195.0658[M-H]$^{-}$(calcd for $C_{10}H_{11}O_4$, 195.0657).

Iso-rhizonic acid, 2-Methoxy-3, 6-dimethyl-4-hydroxybenzoic acid (18): White amorphous Powder :^{1}H NMR (400 MHz, CD_3COCD_3):δ10.30 (1H, s), 6.37 (1H, s),3.99(3H, s), 2.55 (3H,s);^{13}C NMR (100 MHz, CD_3COCD_3):δ195.54, 173.54, 169.36, 167.95, 154.33, 113.38, 110.01, 106.29, 53.89, 25.882ppm.HRESI-MS:*m/z* 195.0658 [M-H]$^{-}$(calcd for $C_{10}H_{11}O_4$, 195.0657).

Atranol (19): White amorphous powder; ^{1}H NMR (300 MHz, CDCl$_3$): δ 10.27 (1H, s), 6.27 (2H, s), 2.25 (3H, s); ^{13}C NMR (75 MHz, CDCl$_3$):δ193.67, 161.85, 150.69, 107.33, 22.08 ppm. HRESI-MS: *m/z* 151.0396 [M-H]$^{-}$(calcd for $C_8H_7O_3$, 151.0395).

Methyl Atratate (20): Yellow solid; ^{1}H NMR (400 MHz, Acetone-d$_6$):δ 11.98 (1H, s), 6.36 (1H, s), 3.92 (3H, s), 2.41 (3H, s), 2.02 (3H, s); ^{13}C NMR (100 MHz, Acetone-d$_6$): δ 174.44, 164.95, 162.30, 141.32, 112.56, 110.33, 105.53, 53.05, 25.17, 9.06; HRESI-MS: *m/z* 196.0654 [M-H]$^{-}$(calcd for $C_{10}H_{11}O_4$, 196.0657).

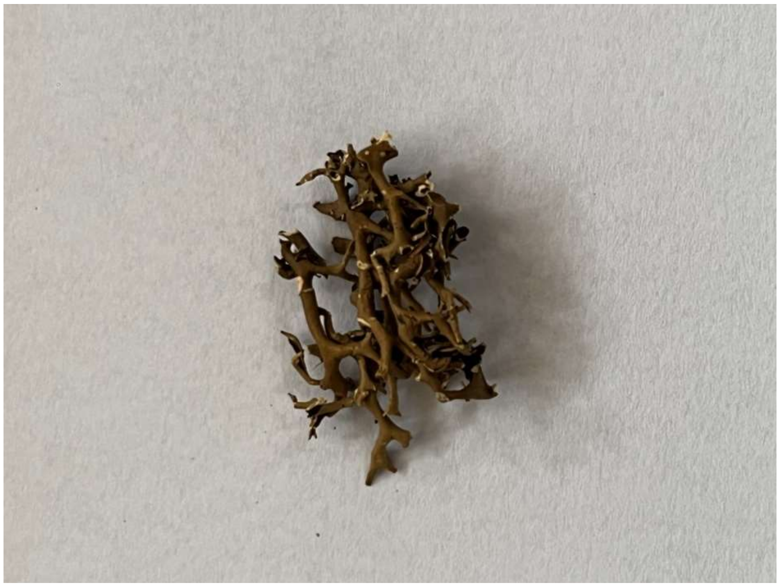

Figure- S1: The authenticate *Hypotrachyna cirrhata*(Fr) Hale

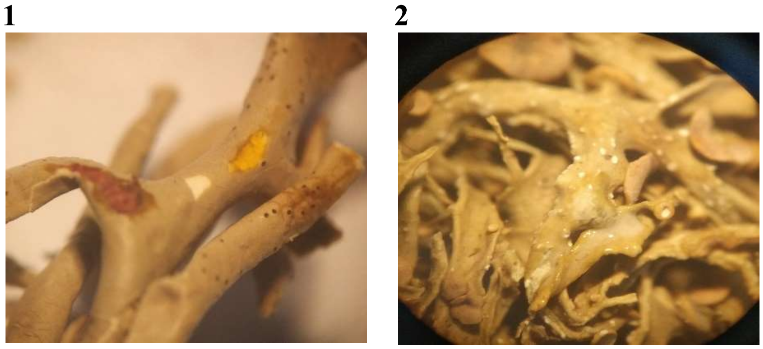

Figure -S2: The spot tests for identification of *Hypotrachyna cirrhata*

1= after K and P positive test (Reddish brown andOrange or Orange red) 2 = after C test (negative).

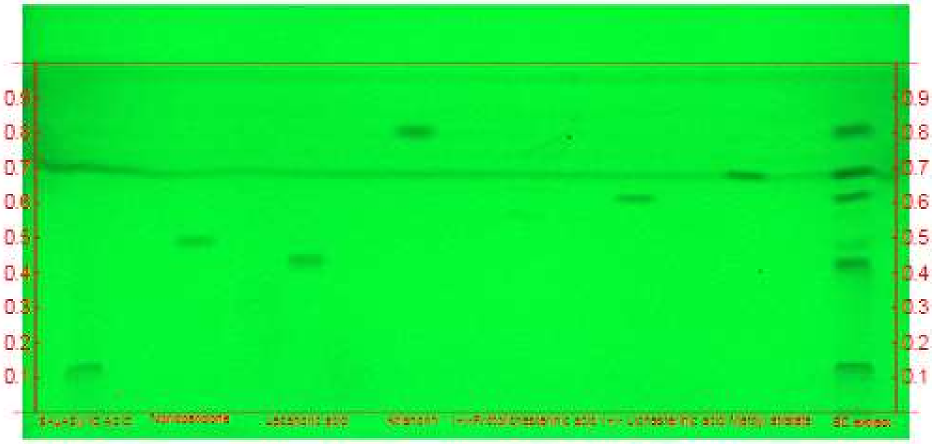

Figure- S3: HPTLC profiles for *Hypotrachynacirrhata*extract along the standard compounds at 254 nm, TLC system: Toluene / 1, 4- Dioxane / Acetic acid – 84.0: 8.0: 8.0.

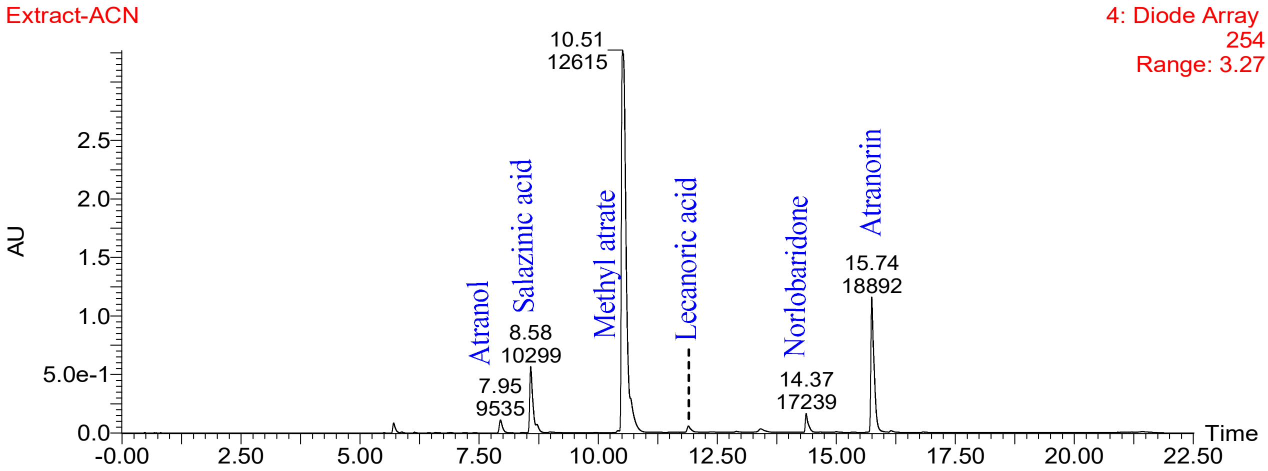

Figure- S4: UPLC-PDA chromatogram of acetone extract of *Hypotrachyna cirrhata*

A)

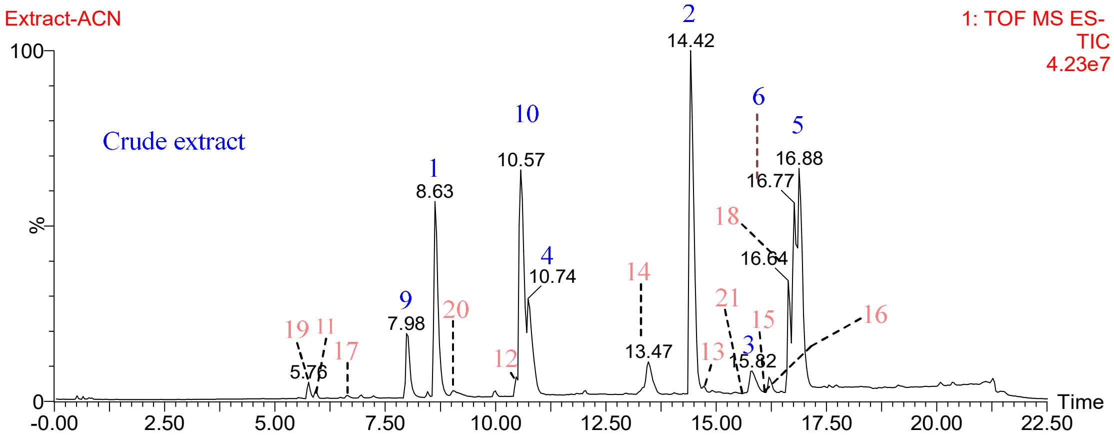

B)

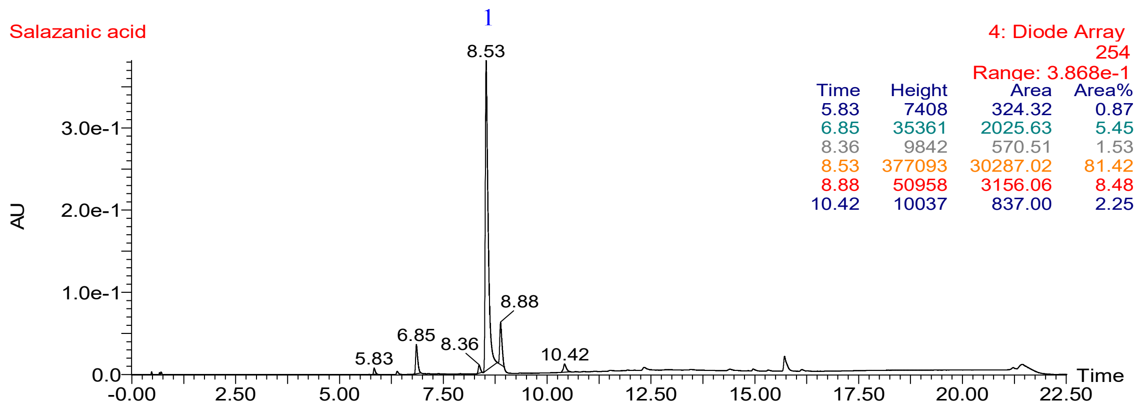
Salazanic acid
1
8.53
4: Diode Array
254
Range: 3.868e-1
Time Height Area Area%
5.83 7408 324.32 0.87
6.85 35361 2025.63 5.45
8.36 9842 570.51 1.53
8.53 377093 30287.02 81.42
8.88 50958 3156.06 8.48
10.42 10037 837.00 2.25
AU
3.0e-1
2.0e-1
1.0e-1
0.0
5.83
6.85
8.36
8.88
10.42
-0.00 2.50 5.00 7.50 10.00 12.50 15.00 17.50 20.00 22.50
Time

C)

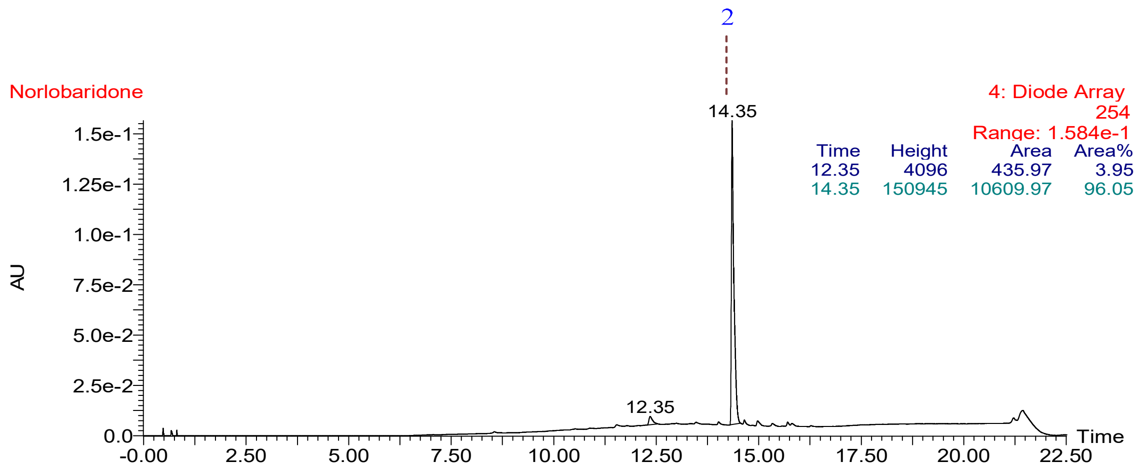
2
Norlobaridone
14.35
4: Diode Array
254
Range: 1.584e-1
Time Height Area Area%
12.35 4096 435.97 3.95
14.35 150945 10609.97 96.05
AU
1.5e-1
1.25e-1
1.0e-1
7.5e-2
5.0e-2
2.5e-2
0.0
12.35
-0.00 2.50 5.00 7.50 10.00 12.50 15.00 17.50 20.00 22.50
Time

D)

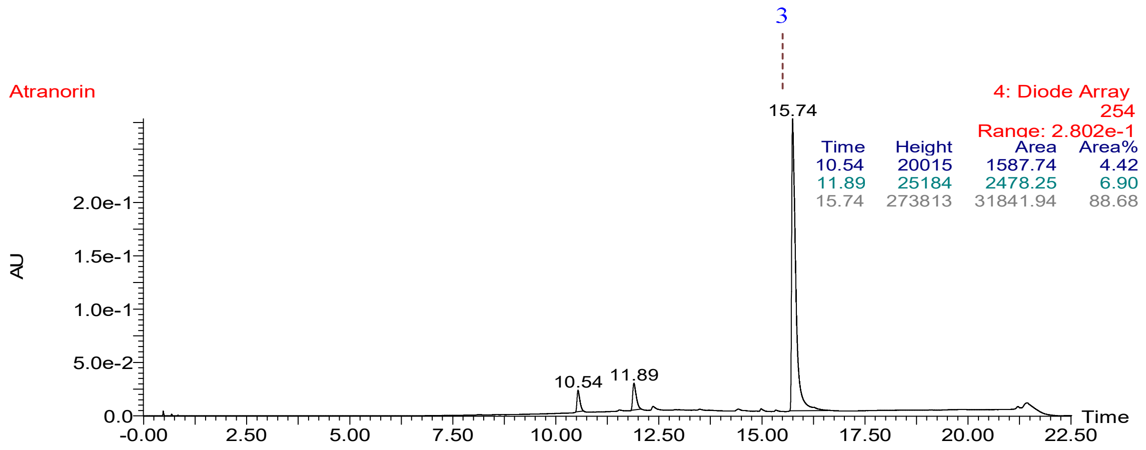
3
Atranorin
15.74
4: Diode Array
254
Range: 2.802e-1
Time Height Area Area%
10.54 20015 1587.74 4.42
11.89 25184 2478.25 6.90
15.74 273813 31841.94 88.68
AU
2.0e-1
1.5e-1
1.0e-1
5.0e-2
0.0
10.54
11.89
-0.00 2.50 5.00 7.50 10.00 12.50 15.00 17.50 20.00 22.50
Time

E)

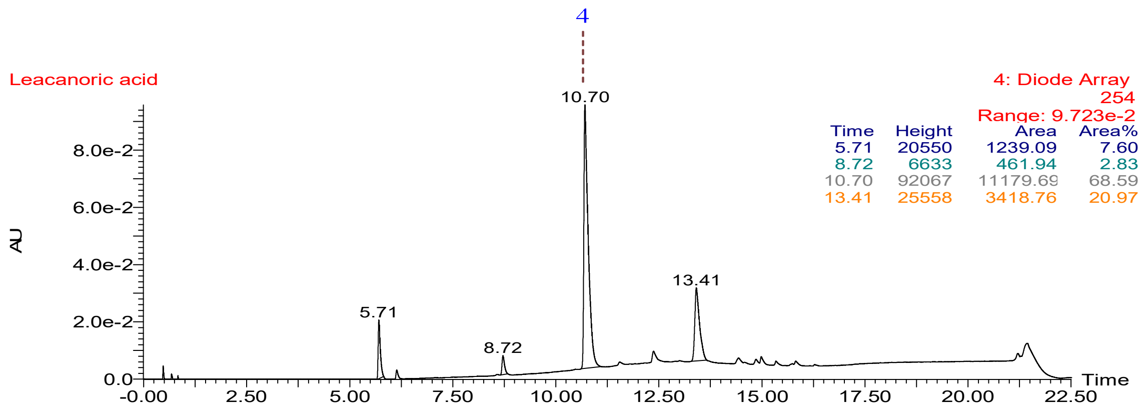
4
Leacanoric acid
4: Diode Array
254
Range: 9.723e-2
Time Height Area Area%
5.71 20550 1239.09 7.60
8.72 6633 461.94 2.83
10.70 92067 11179.69 68.59
13.41 25558 3418.76 20.97
10.70
13.41
5.71
8.72
AU
8.0e-2
6.0e-2
4.0e-2
2.0e-2
0.0
-0.00 2.50 5.00 7.50 10.00 12.50 15.00 17.50 20.00 22.50
Time

F)

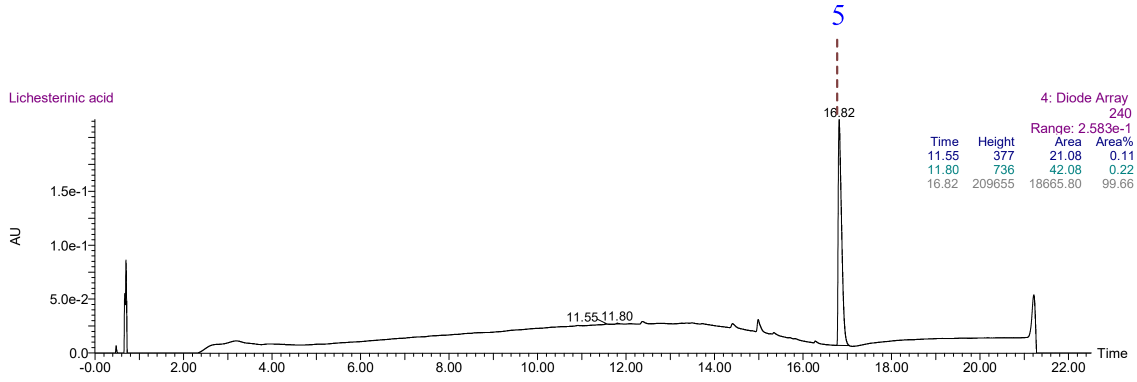
5
Lichesterinic acid
4: Diode Array
240
Range: 2.583e-1
Time Height Area Area%
11.55 377 21.08 0.11
11.80 736 42.08 0.22
16.82 209655 18665.80 99.66
16.82
11.55 11.80
AU
1.5e-1
1.0e-1
5.0e-2
0.0
-0.00 2.00 4.00 6.00 8.00 10.00 12.00 14.00 16.00 18.00 20.00 22.00
Time

G)

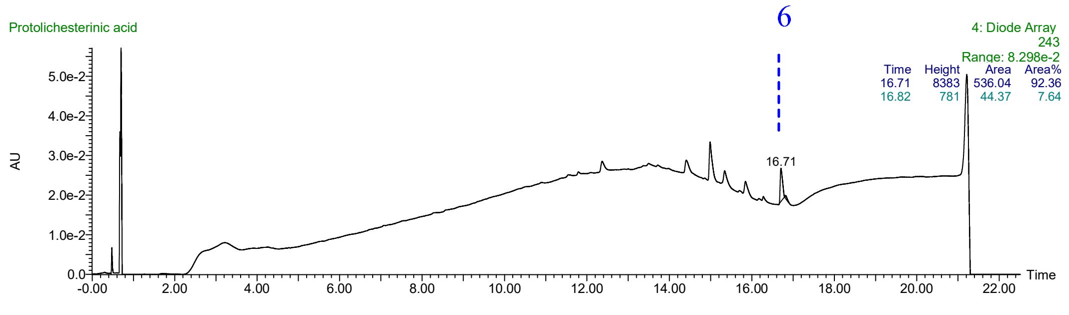
6
Protolichesterinic acid
4: Diode Array
243
Range: 8.298e-2
Time Height Area Area%
16.71 8383 536.04 92.36
16.82 781 44.37 7.64
16.71
AU
5.0e-2
4.0e-2
3.0e-2
2.0e-2
1.0e-2
0.0
-0.00 2.00 4.00 6.00 8.00 10.00 12.00 14.00 16.00 18.00 20.00 22.00
Time

H)

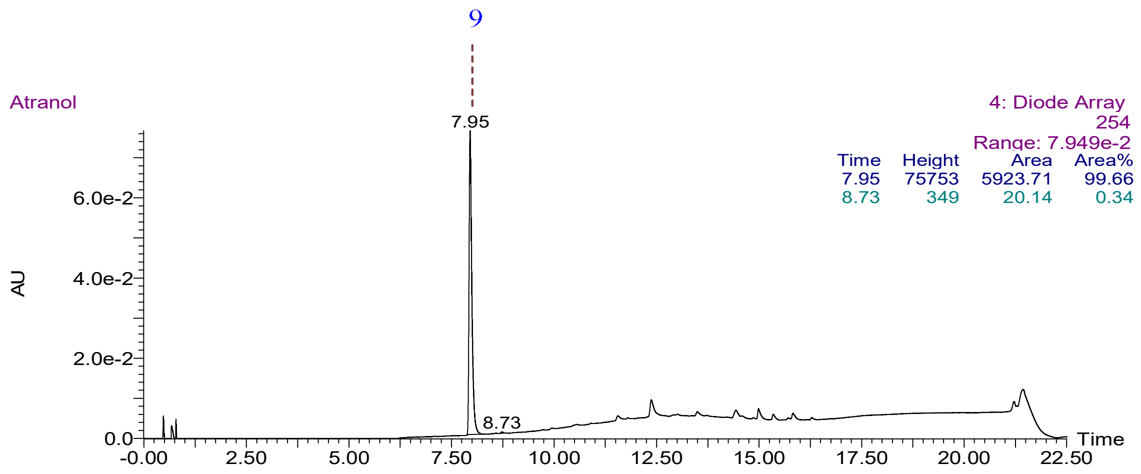

I)

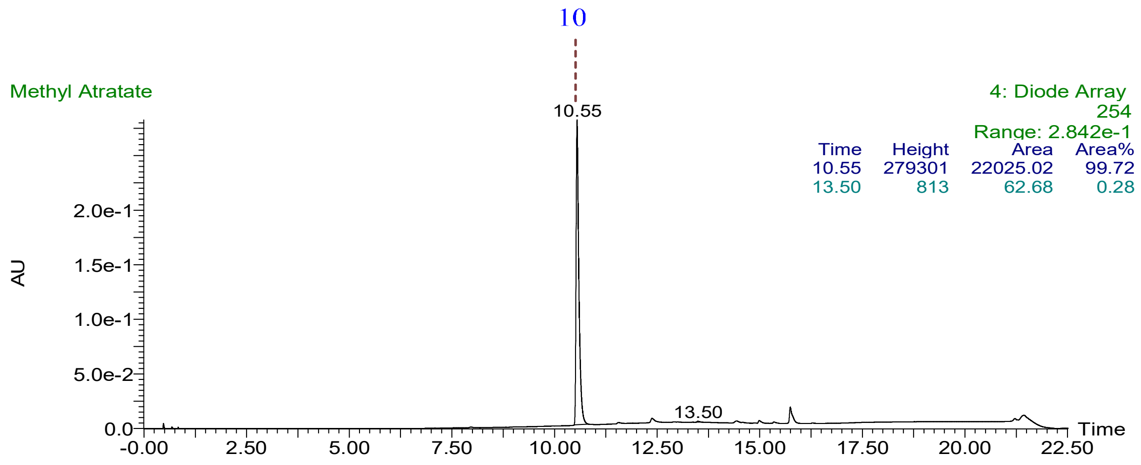

Figure- S5: Shows the Total Ion Chromatogram (TIC) acetone extract of *Hypotrachynacirrhata* and pure compounds. A= Extract* = Isolated compounds, & =identified ms/ms compounds; B=Compound 1; C=Compound 2; D= Compound 3; E= Compound 4; F = Compound 5; G = Compound 6; H = Compound 9; I = Compound 10;

Minimum:				-1.5				
Maximum:		5.0	5.0	50.0				
Mass	Calc. Mass	mDa	PPM	DBE	i-FIT	Norm	Conf(%)	Formula
387.0357	387.0352	0.5	1.3	13.5	863.6	n/a	n/a	C18 H11 O10

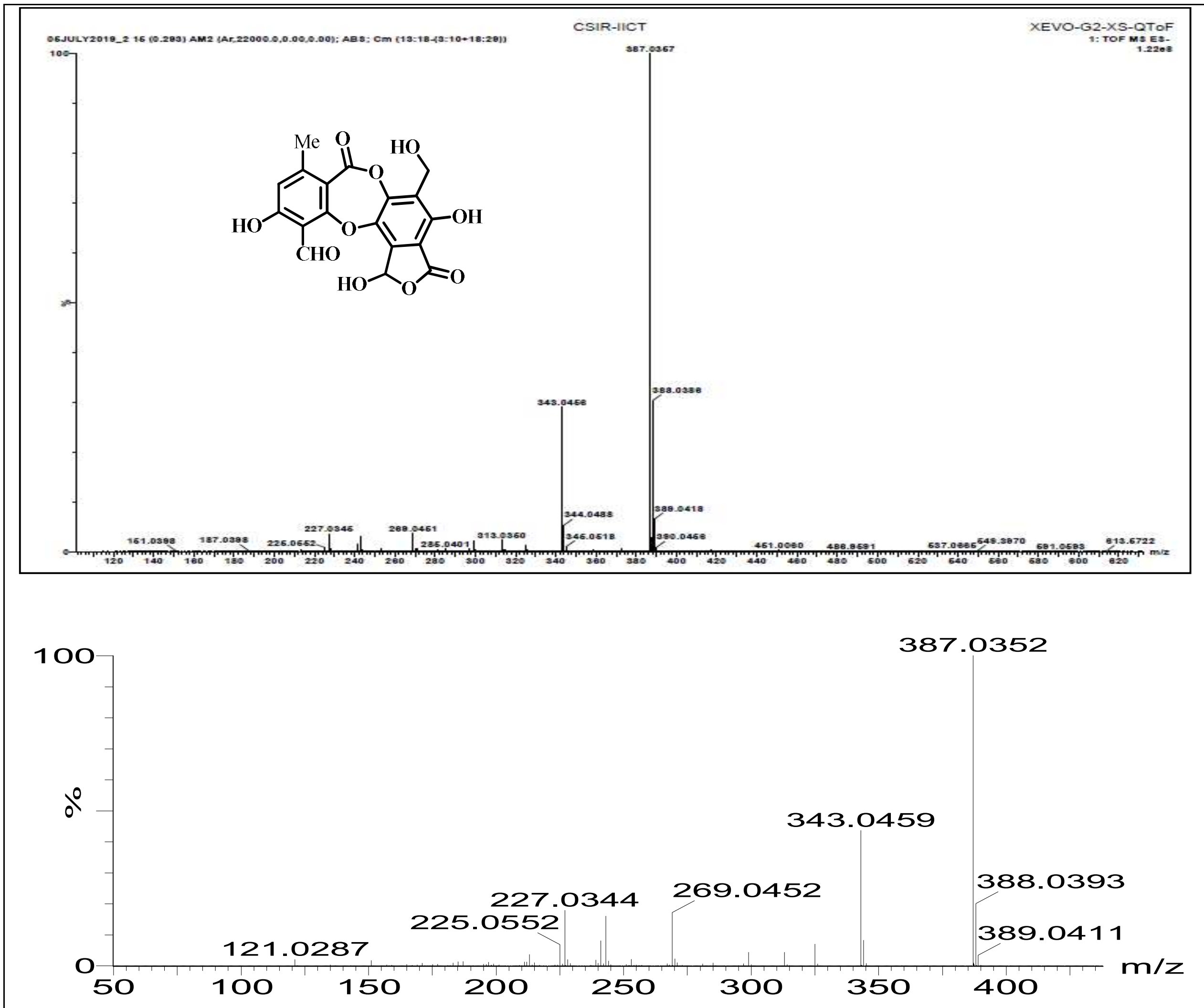

Figure- S6: HRESIMS, MS/MS spectra and fragmentation pattern of Salazinicacid (1)

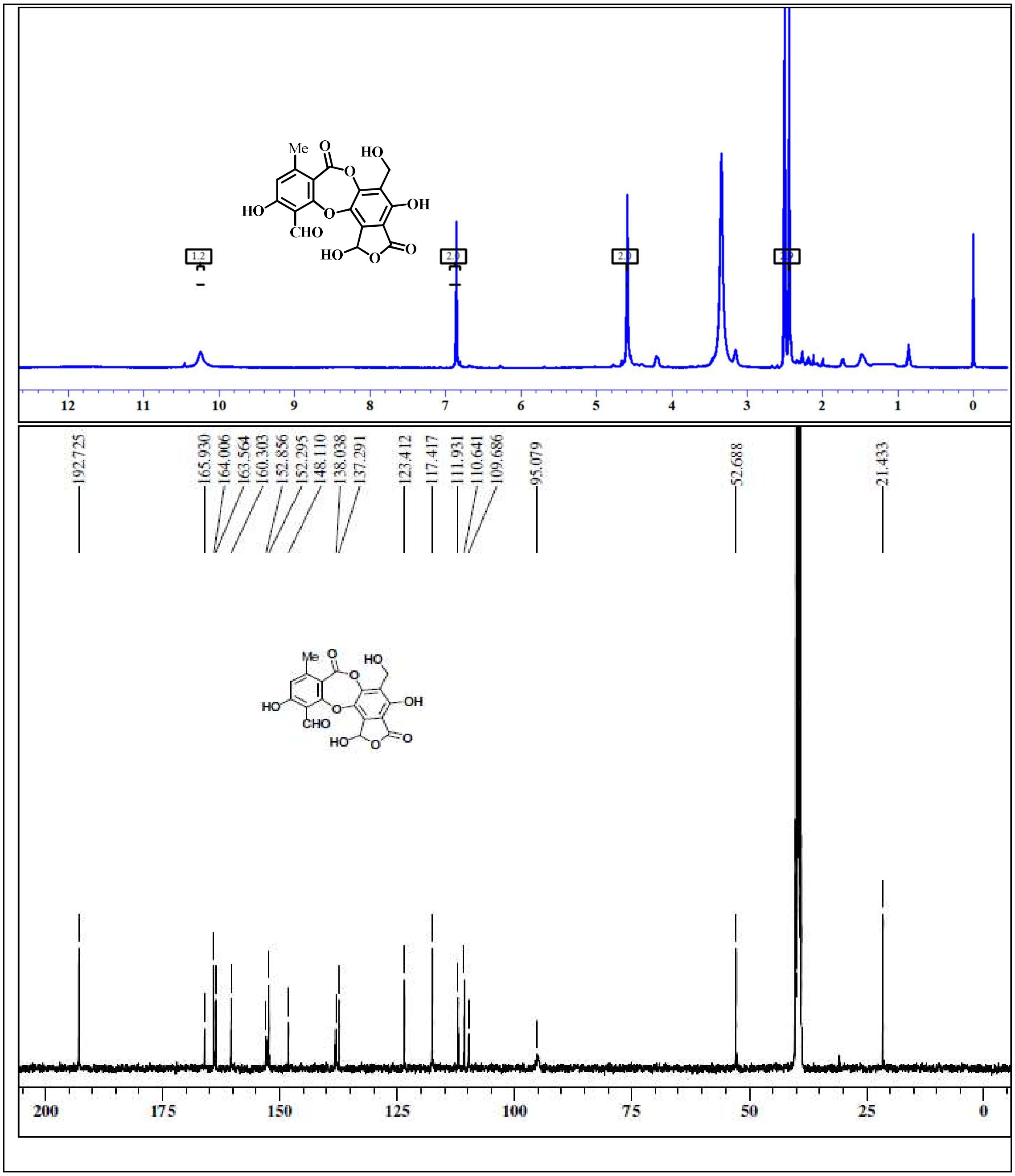

Figure- S7:^{1}H &^{13}C NMR spectrum of Salazinicacid (**11**) (500 & 100 MHz, DMSO-d6)

Minimum:				-1.5				
Maximum:		5.0	5.0	50.0				
Mass	Calc. Mass	mDa	PPM	DBE	i-FIT	Norm	Conf(%)	Formula
397.1647	397.1651	-0.4	-1.0	11.5	865.0	n/a	n/a	C23 H25 O6

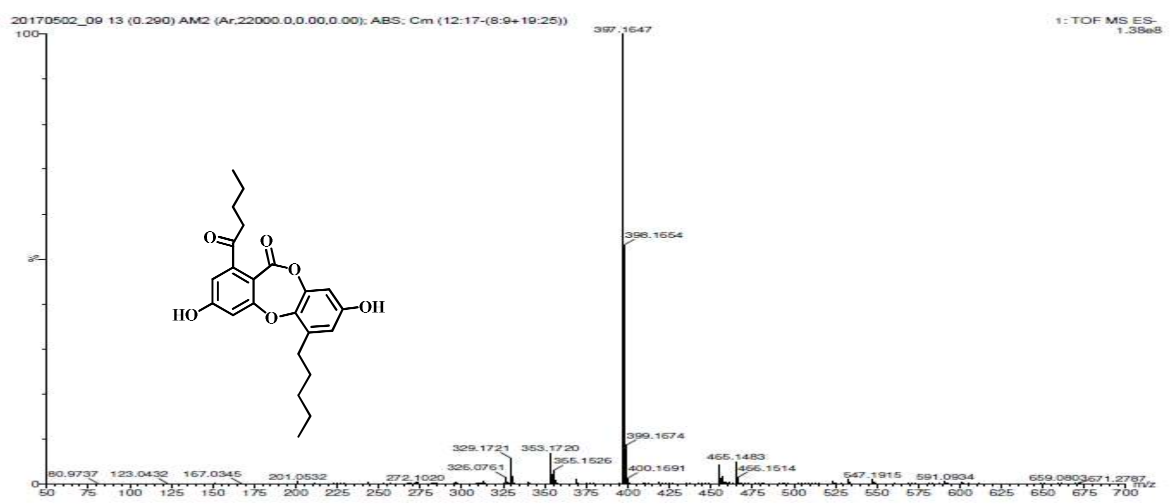

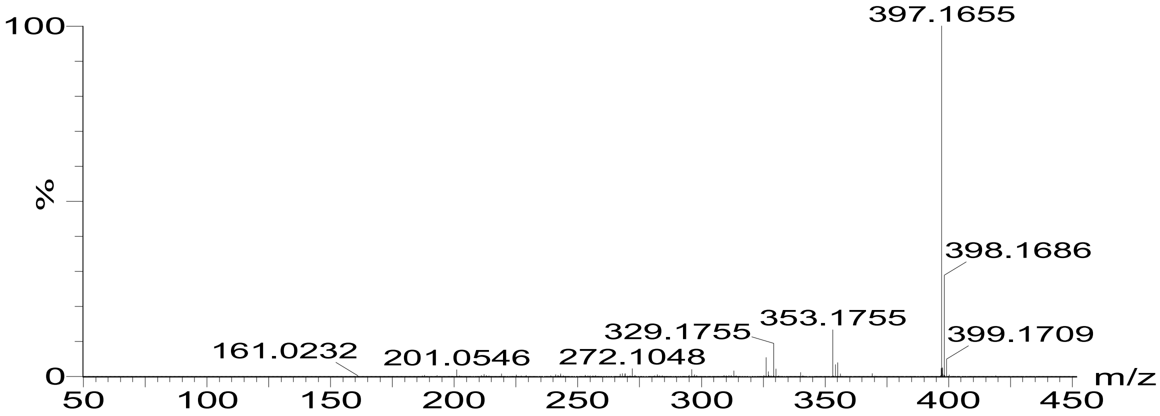

Figure- S8: HRESIMS, MS/MS spectra and fragmentation pattern of Norlobaridone (**12**)

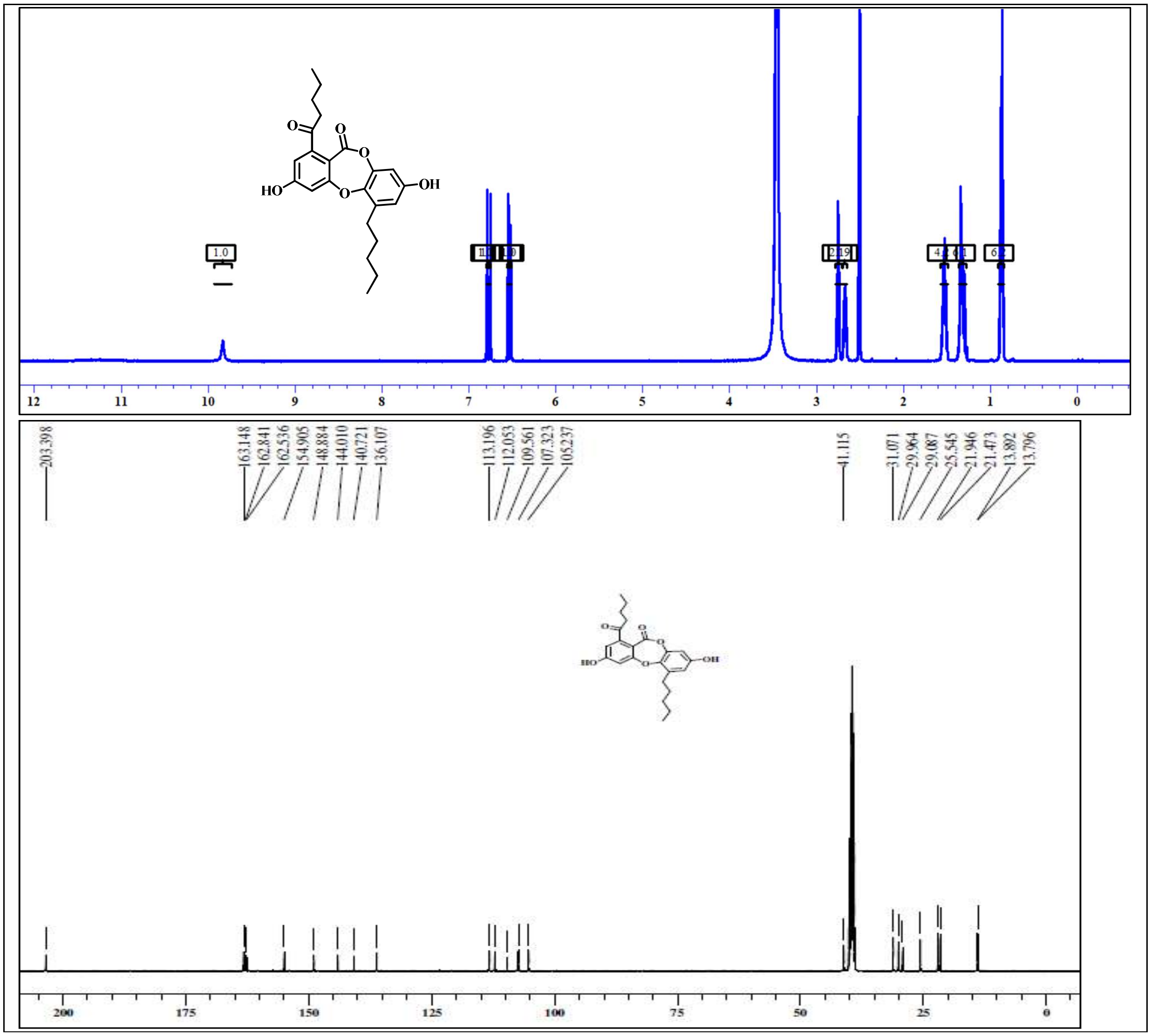

Figure -S9:^{1}H &^{13}C NMR Spectrum of Norlobaridone (**12**) (500 & 100 MHz, DMSO-d6)

Minimum:				-1.5				
Maximum:		5.0	5.0	50.0				
Mass	Calc. Mass	mDa	PPM	DBE	i-FIT	Norm	Conf(%)	Formula
373.0927	373.0923	0.4	1.1	11.5	666.3	n/a	n/a	C19 H17 O8

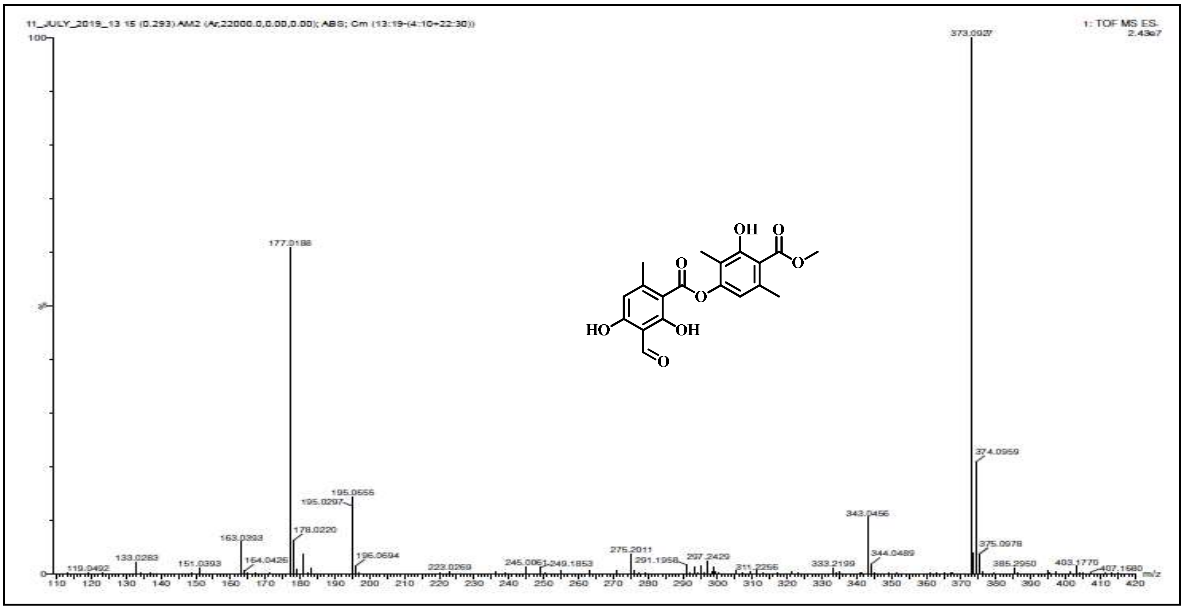

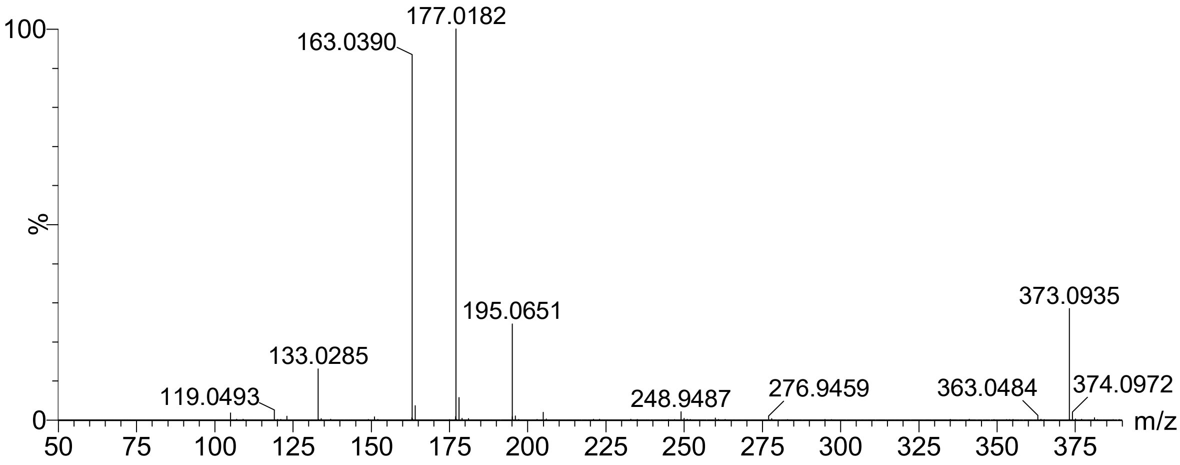

Figure -S10: HRESIMS, MS/MS spectra and fragmentation pattern of Atranorin (13)

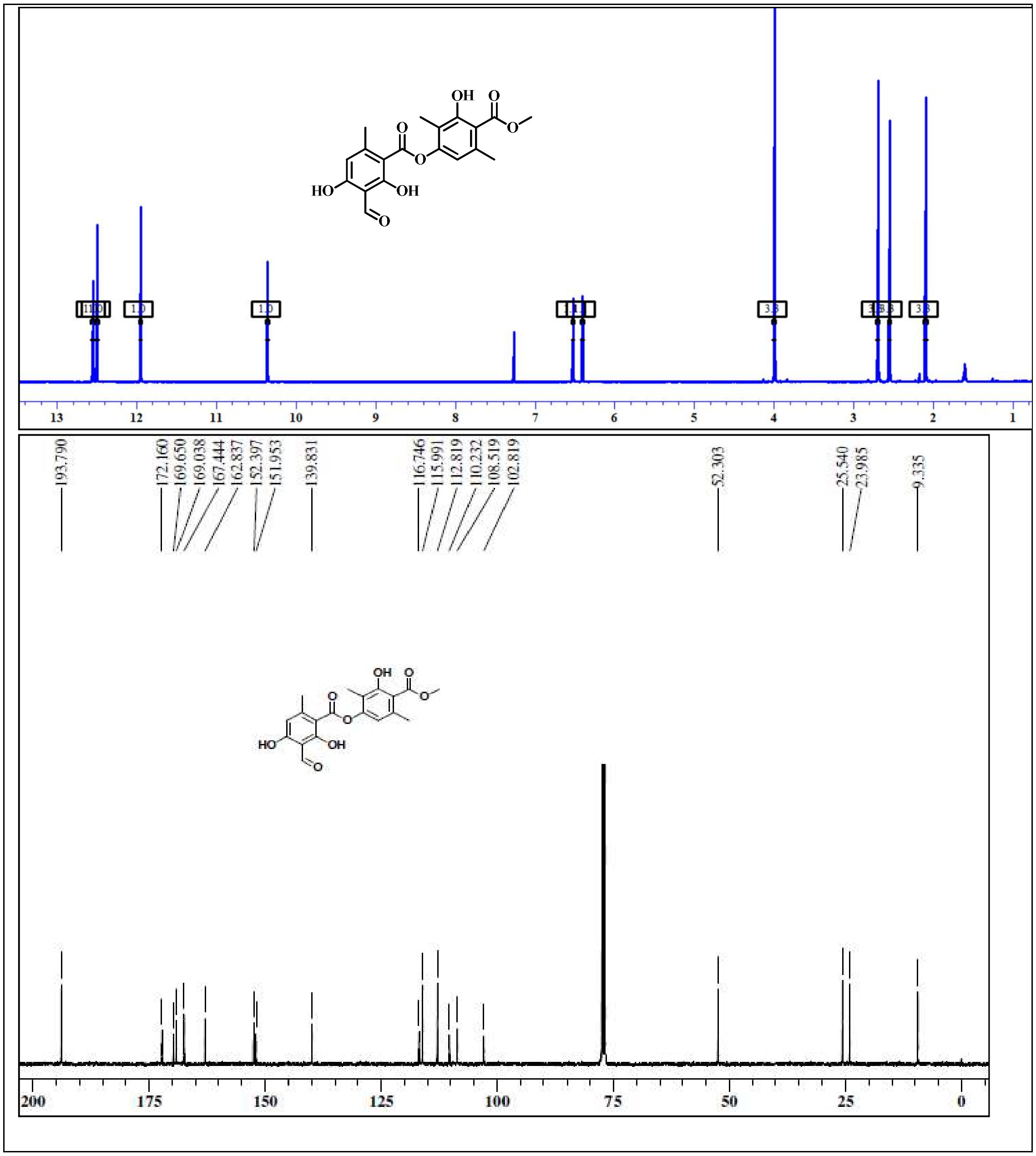

Figure -S11:^{1}H &^{13}C NMR Spectrum of Atranorin (**13**) (500 & 100 MHz, $CDCl_3$)

Minimum:				-1.5				
Maximum:		5.0	5.0	50.0				
Mass	Calc. Mass	mDa	PPM	DBE	i-FIT	Norm	Conf(%)	Formula
317.0667	317.0661	0.6	1.9	10.5	245.3	n/a	n/a	C16 H13 O7

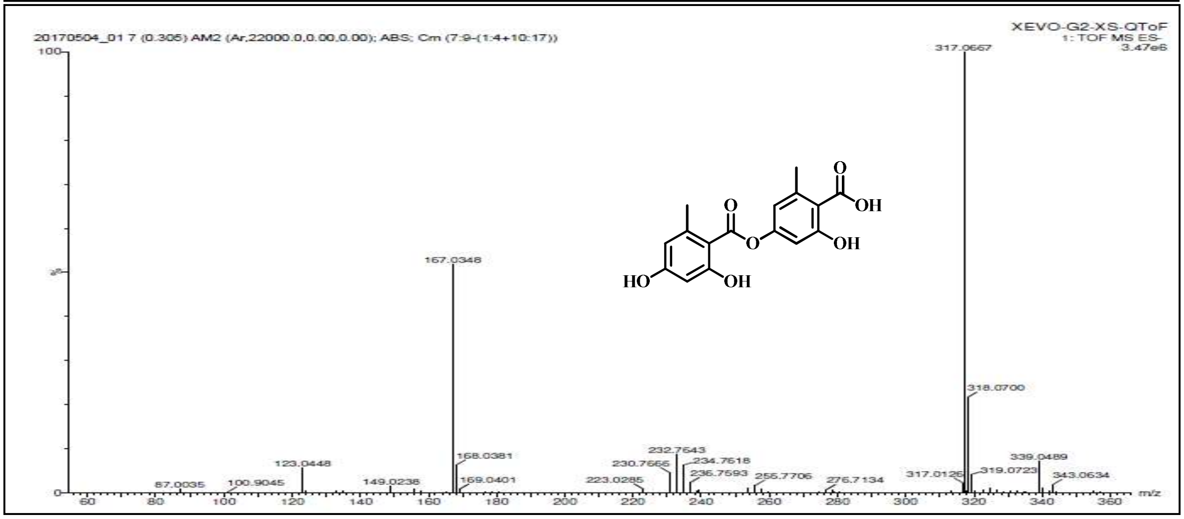

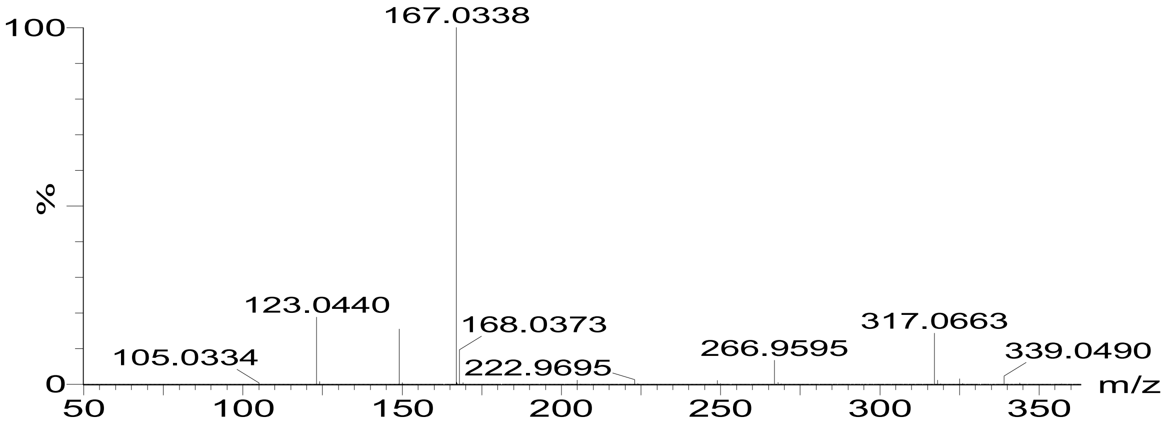

Figure- S12: HRESIMS, MS/MS spectra and fragmentation pattern of Lecanoric acid (**14**)

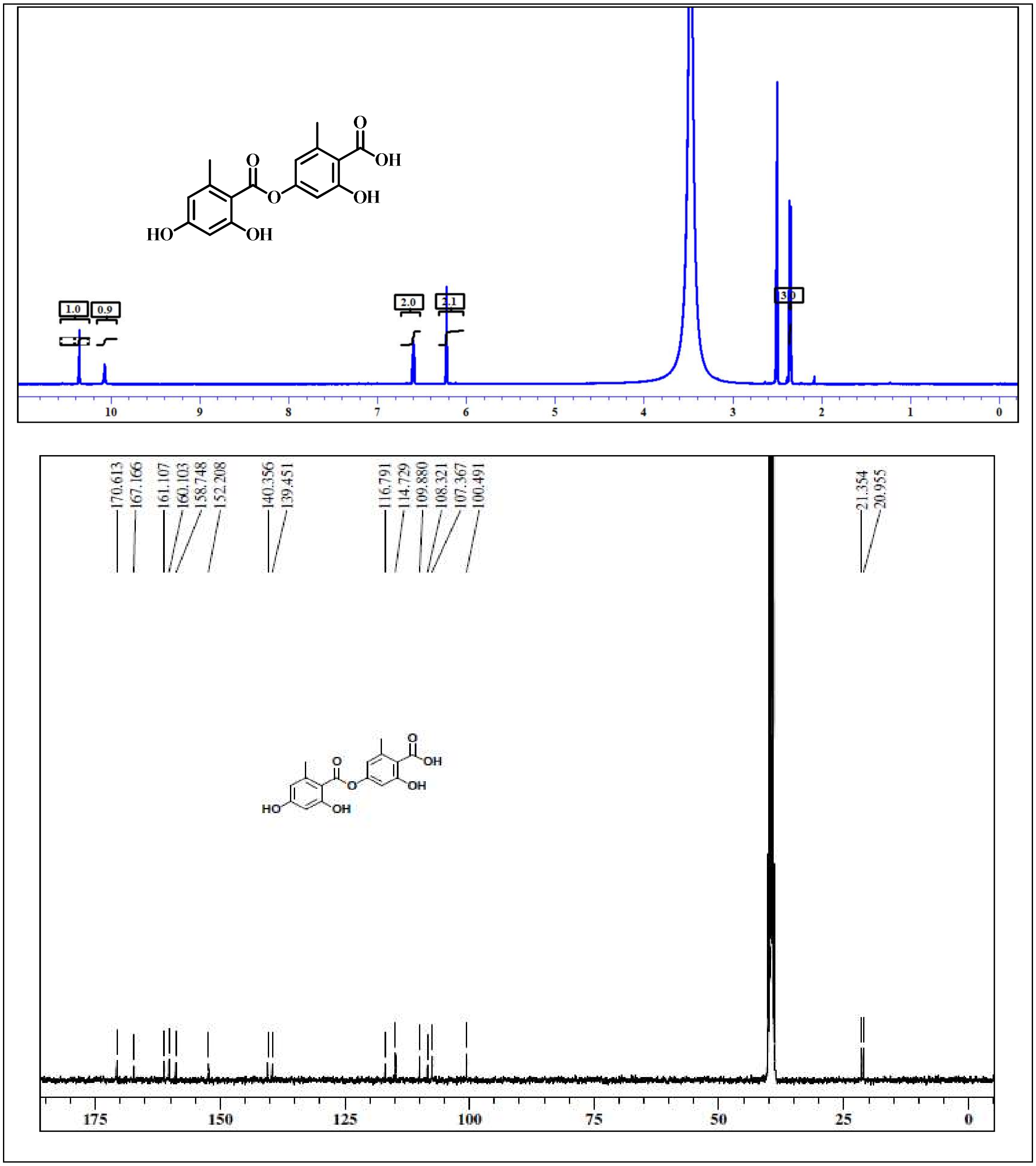

Figure -S13: ^{1}H & ^{13}C NMR Spectrum of Lecanoric acid (**14**) (500 & 125 MHz, DMSO-d6)

	Mass	Calc. Mass	mDa	PPM	DBE	i-FIT	Norm	Conf(%)	Formula
Minimum:					-1.5				
Maximum:			5.0	10.0	50.0				
	323.2221	323.2222	-0.1	-0.3	4.5	968.9	n/a	n/a	C19 H31 O4

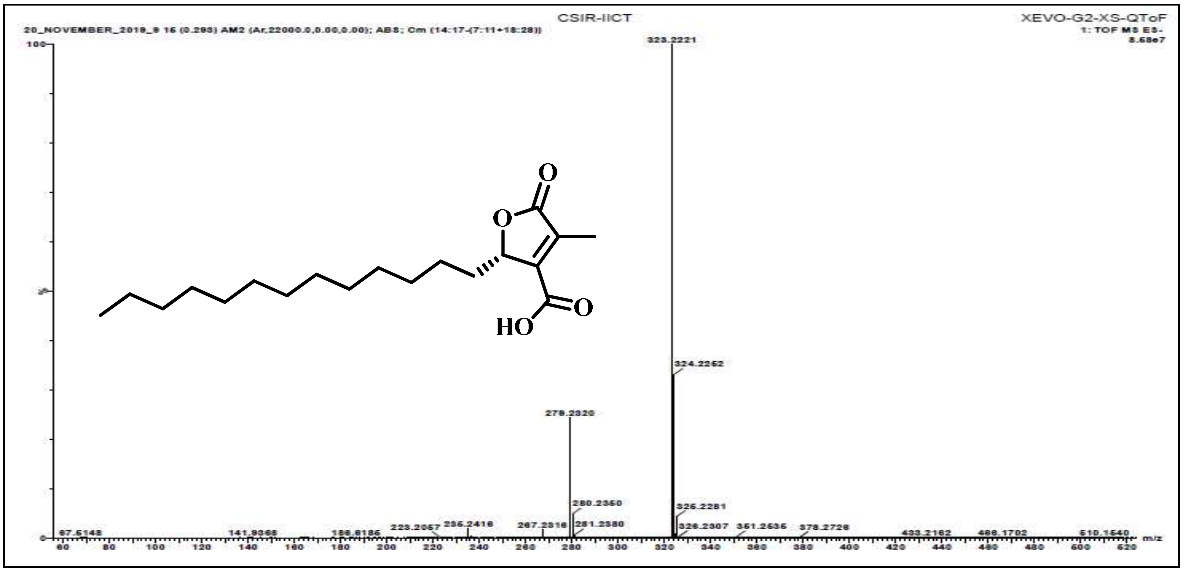

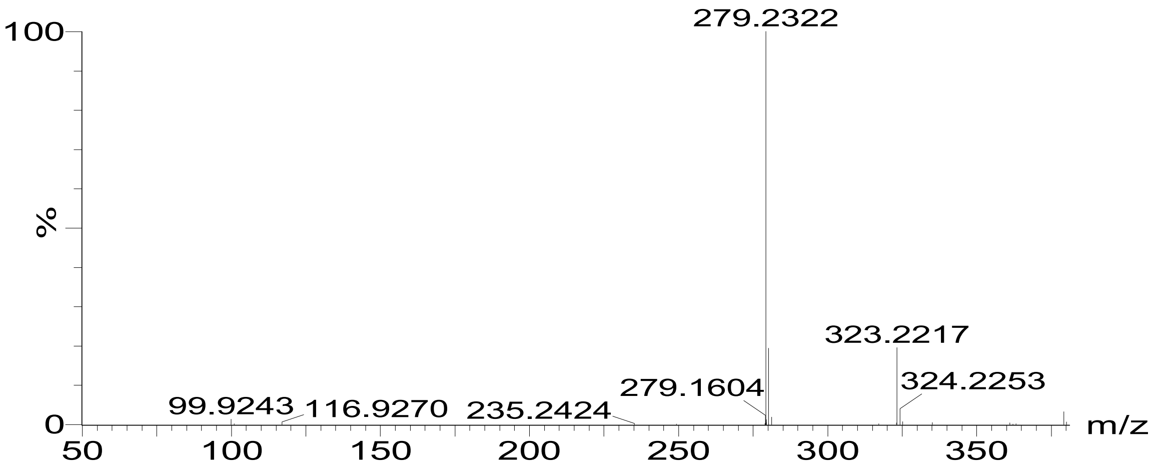

Figure -S14: HRESIMS, MS/MS spectra and fragmentation pattern of Lichesterinic acid (**15**)

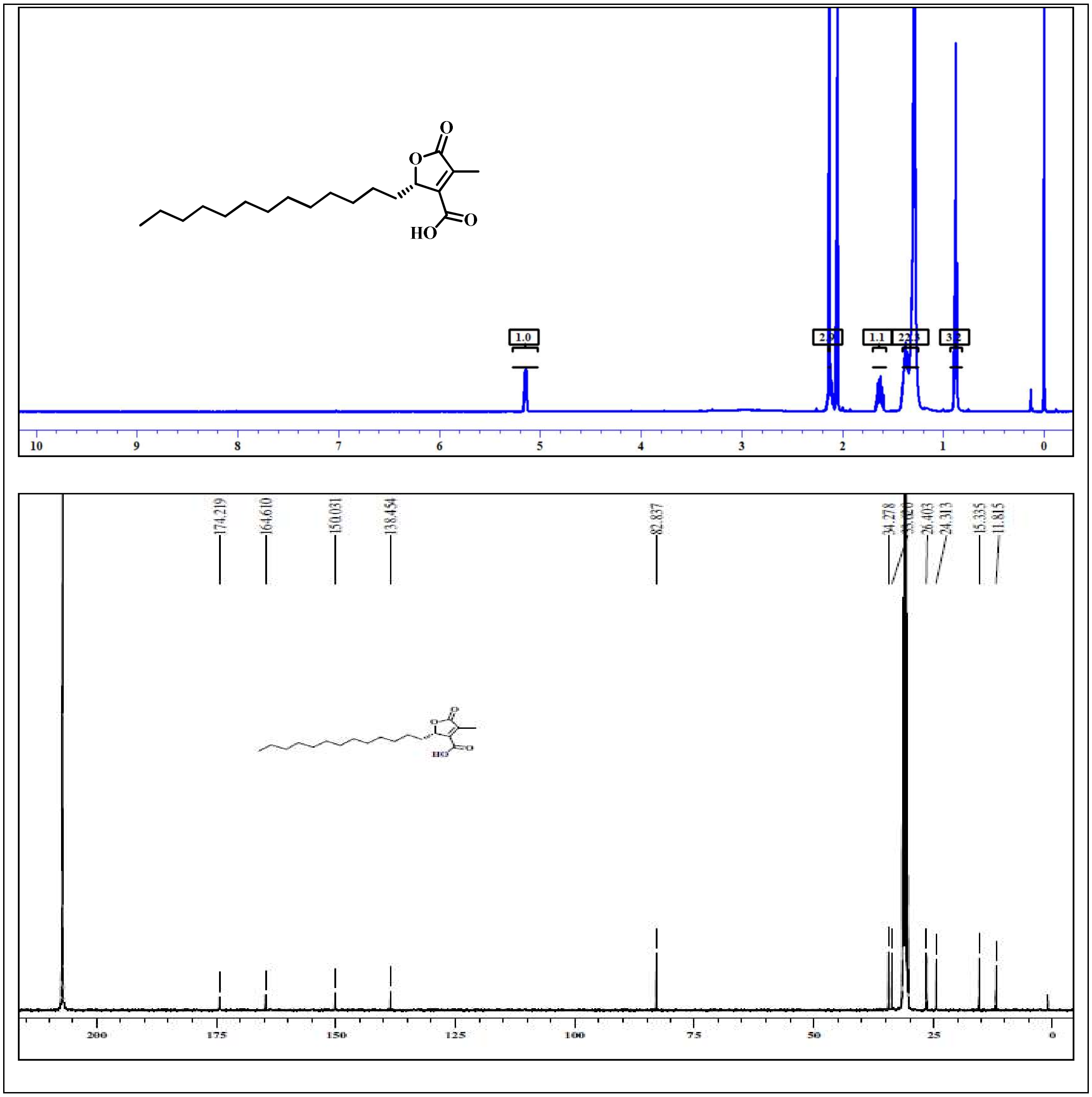

Figure -S15:^{1}H &^{13}C NMR spectrum of Lichesterinic acid (**15**)(400 & 100 MHz, CD_3COCD_3)

Minimum:				-1.5				
Maximum:		5.0	10.0	50.0				
Mass	Calc. Mass	mDa	PPM	DBE	i-FIT	Norm	Conf(%)	Formula
323.2223	323.2222	0.1	0.3	4.5	914.9	n/a	n/a	C19 H31 O4

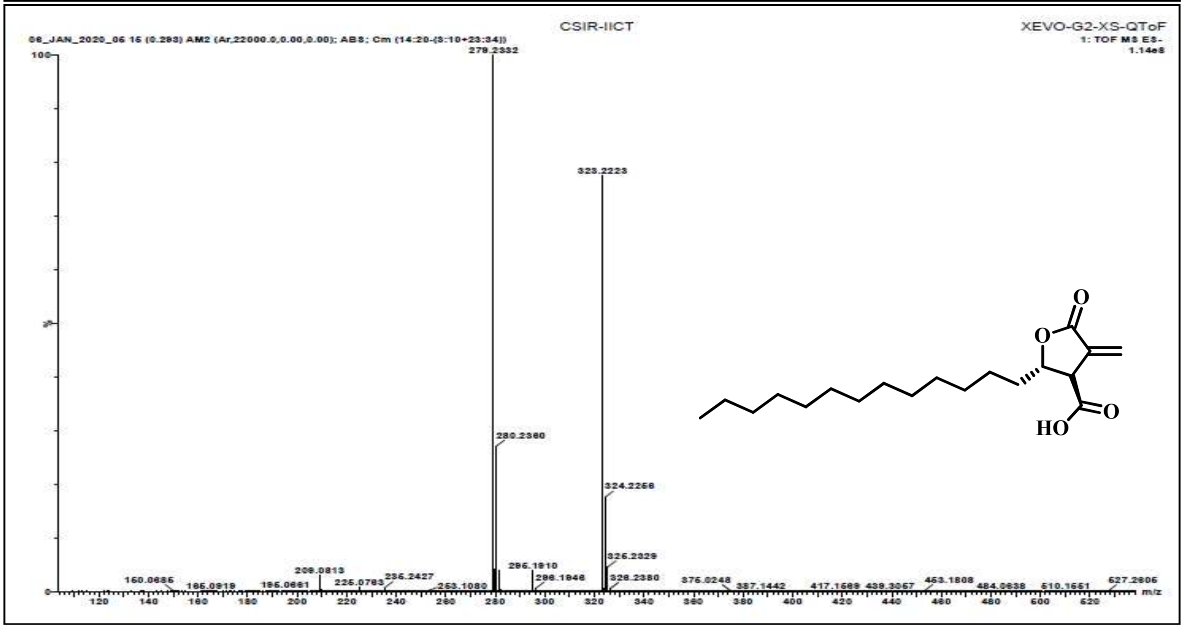

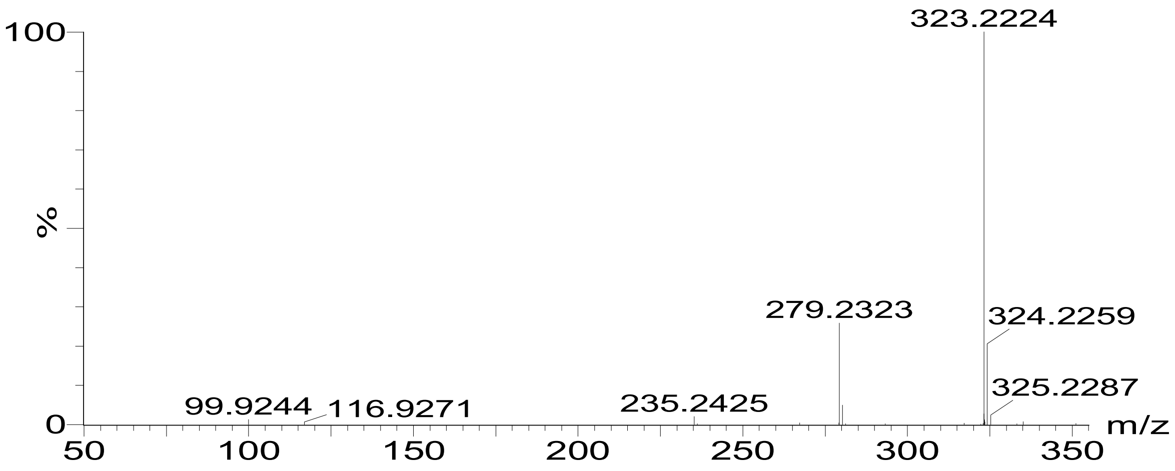

Figure -S16: HRESIMS, MS/MS spectra and fragmentation pattern of Protolichesterinic acid (**16**)

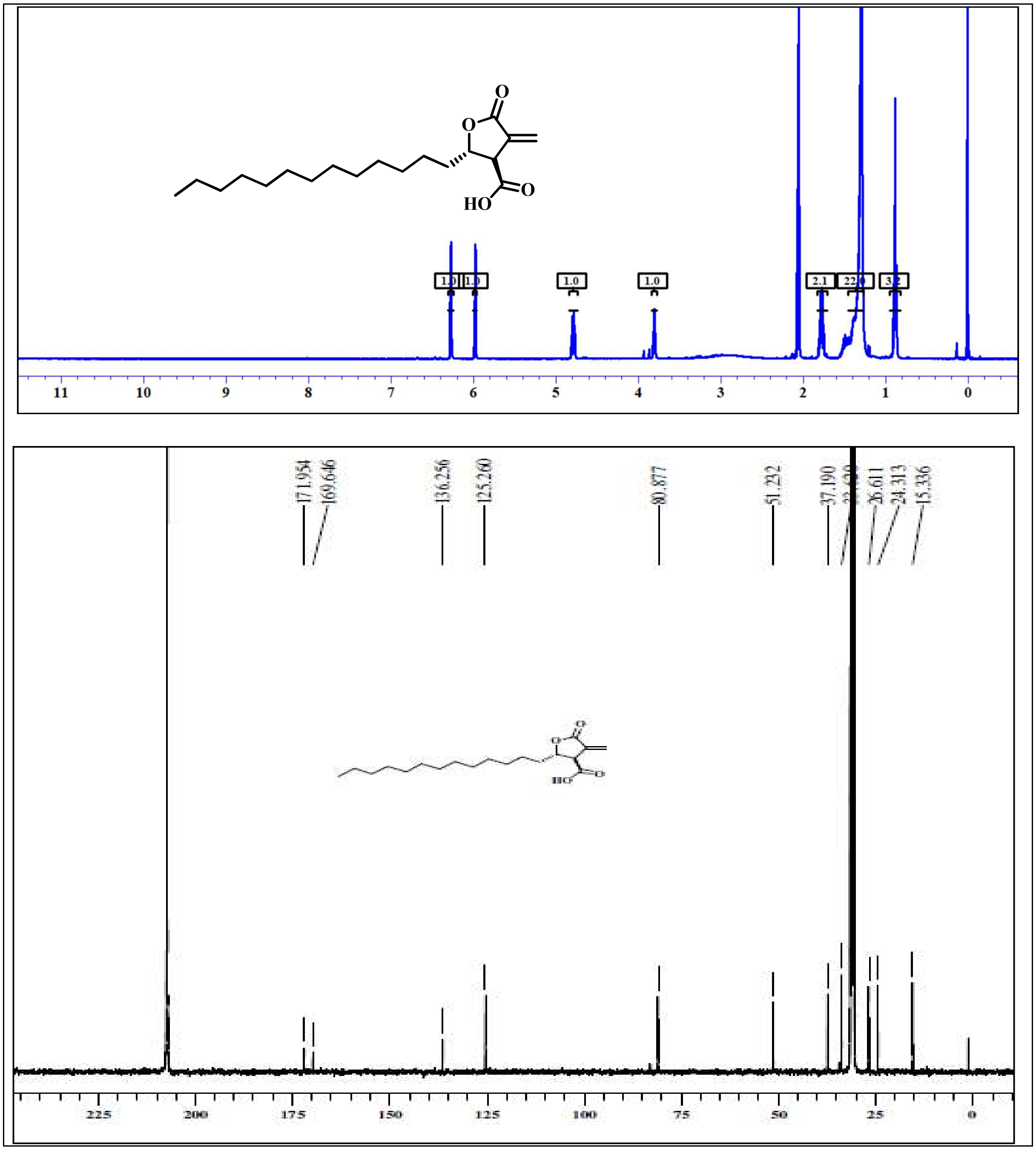

Figure- S17: ^{1}H & ^{13}C NMR spectrum of Protolichesterinic acid (**16**) (400 & 100 MHz, CD_3COCD_3)

Minimum:				-1.5				
Maximum:		5.0	5.0	50.0				
Mass	Calc. Mass	mDa	PPM	DBE	i-FIT	Norm	Conf(%)	Formula
209.0455	209.0450	0.5	2.4	6.5	668.1	n/a	n/a	C10 H9 O5

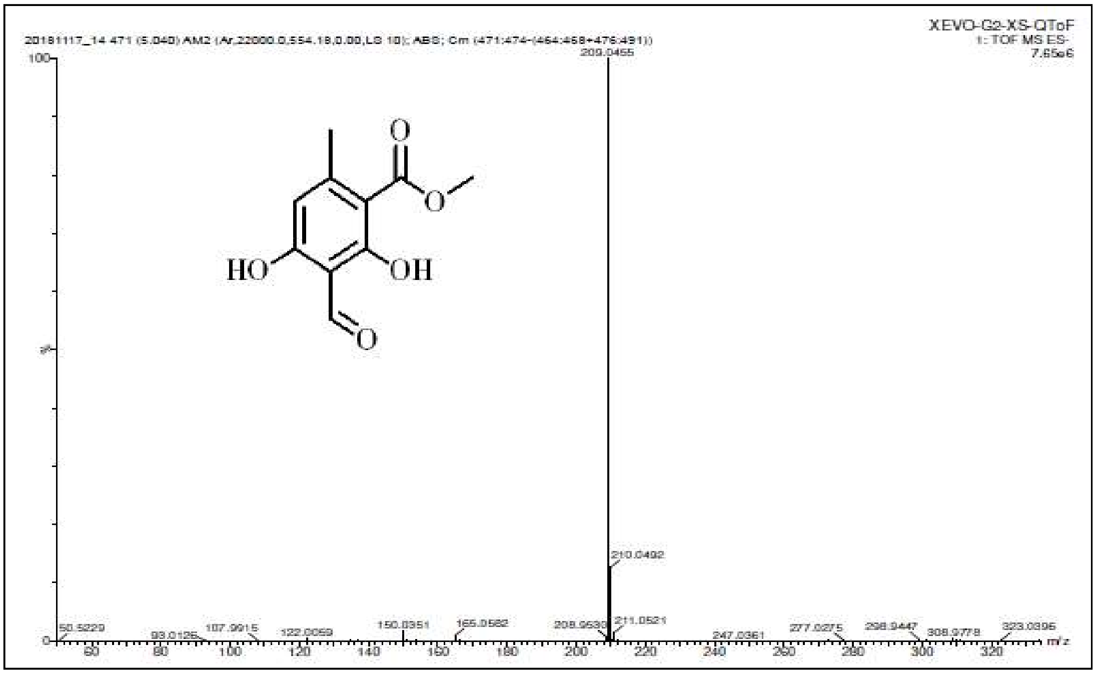

Figure- S18: HRESIMS Spectrum of Methyl hematommate (**17**)

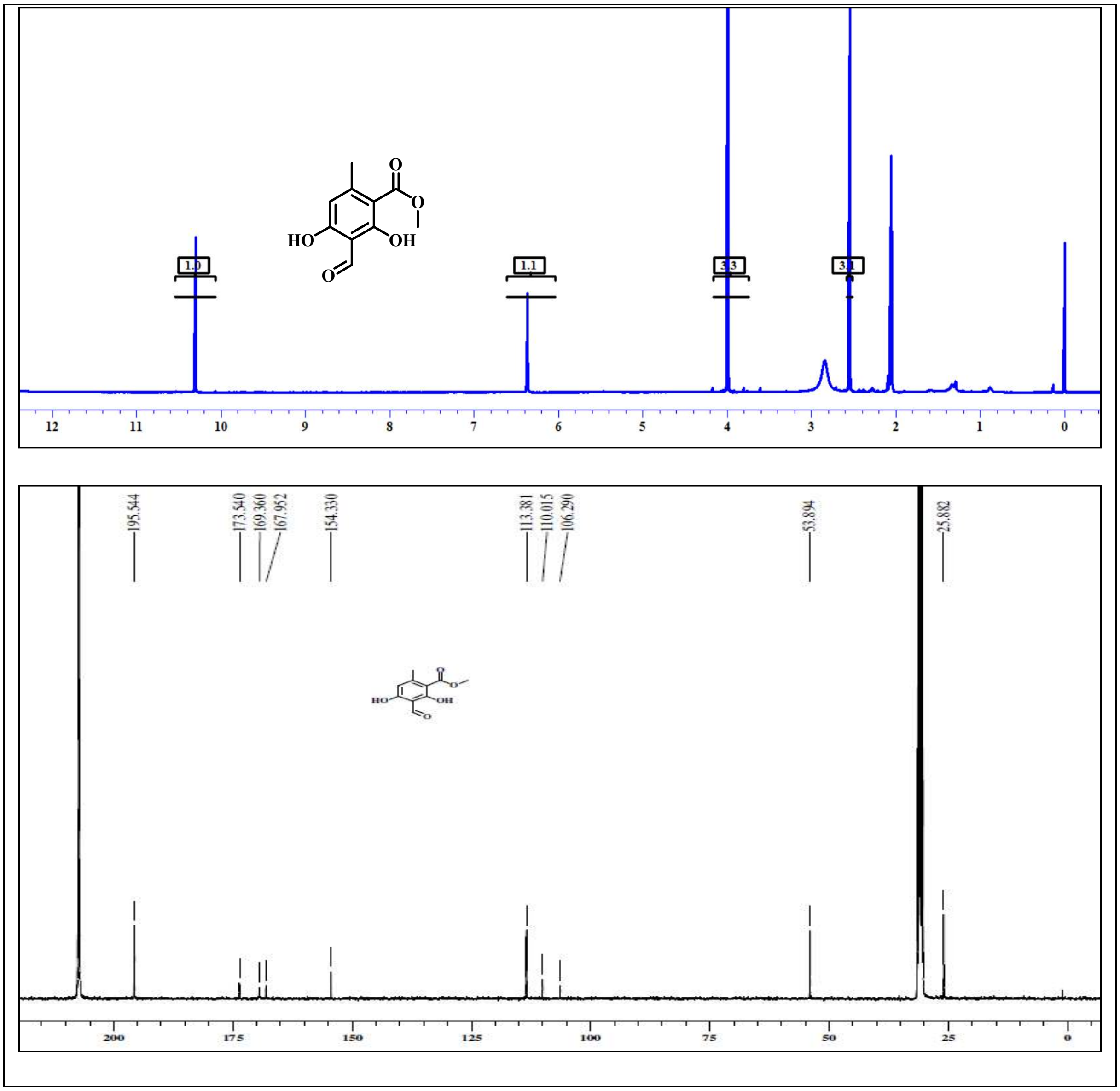

Figure S19:^{1}H &^{13}C NMR Spectrum of Methyl hematommate (**17**) (400 & 100 MHz, Acetone-d_6)

Minimum:				-1.5				
Maximum:		5.0	5.0	50.0				
Mass	Calc. Mass	mDa	PPM	DBE	i-FIT	Norm	Conf(%)	Formula
195.0658	195.0657	0.1	0.5	5.5	428.4	n/a	n/a	C10 H11 O4

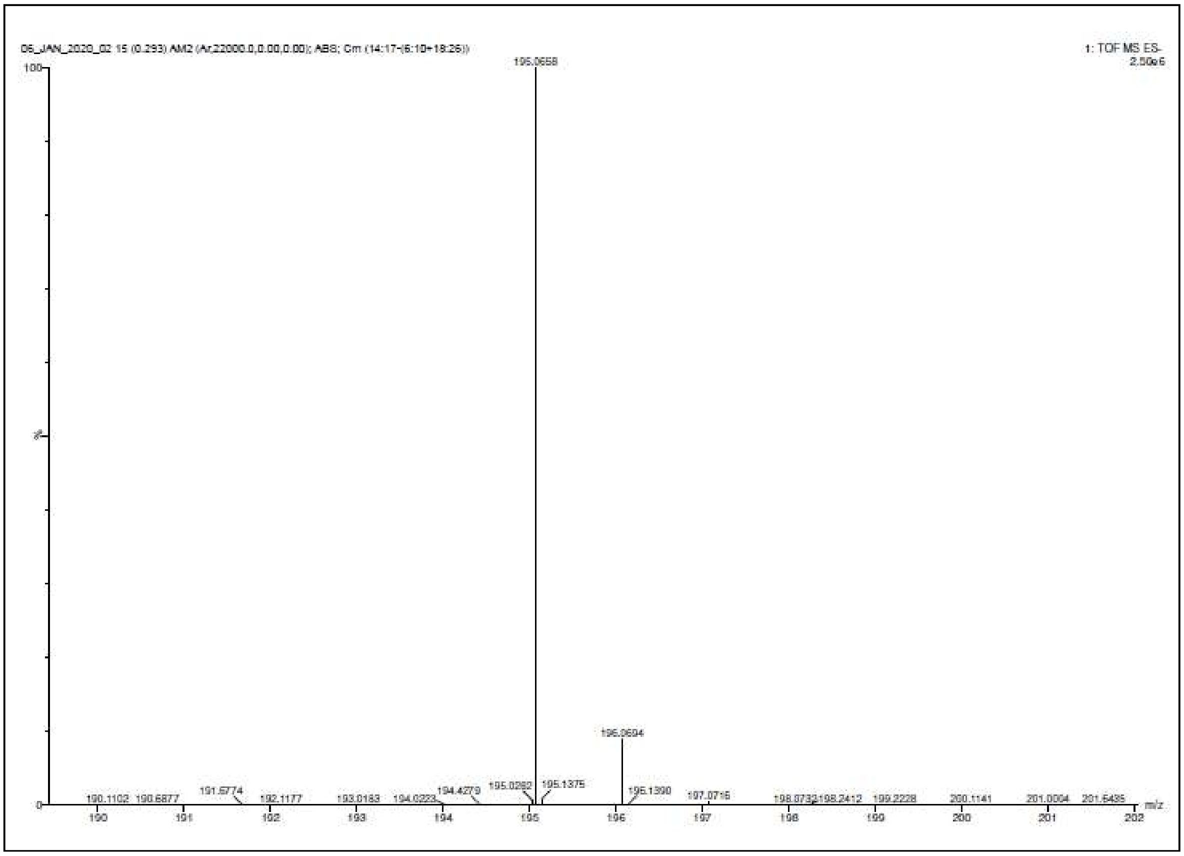

Figure -S20: HRESIMS Spectrum of Iso-rhizonic acid, 2-Methoxy-3, 6-dimethyl-4-Hydroxybenzoic acid (**18**)

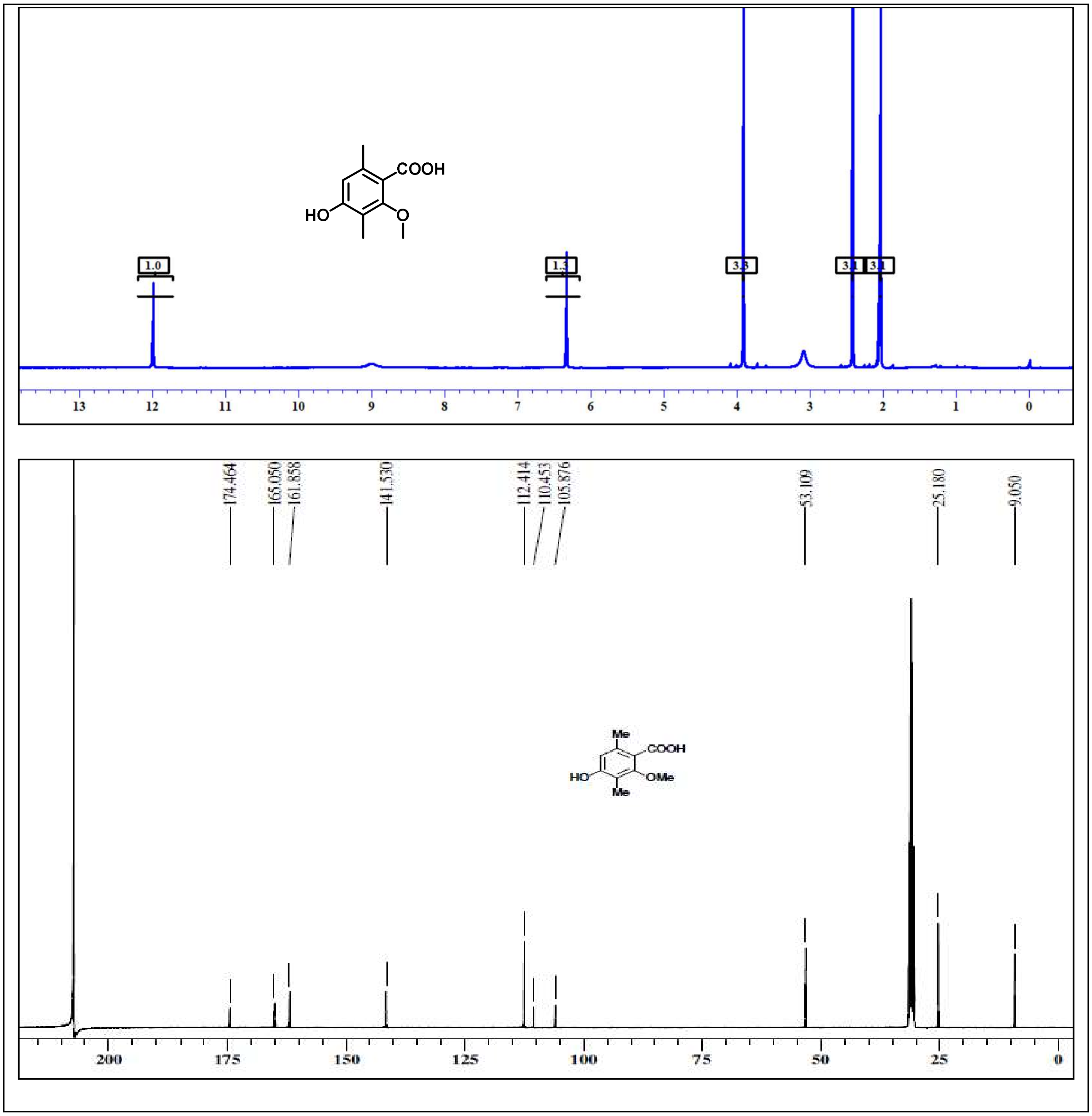

Figure- S21: ^{1}H & ^{13}C NMR Spectrum of Iso-rhizonic acid, 2-Methoxy-3, 6-dimethyl-4-Hydroxybenzoic acid (18) (400 & 100 MHz, Acetone-d_6)

Minimum:				-1.5				
Maximum:		5.0	5.0	50.0				
Mass	Calc. Mass	mDa	PPM	DBE	i-FIT	Norm	Conf(%)	Formula
151.0396	151.0395	0.1	0.7	5.5	181.1	n/a	n/a	C8 H7 O3

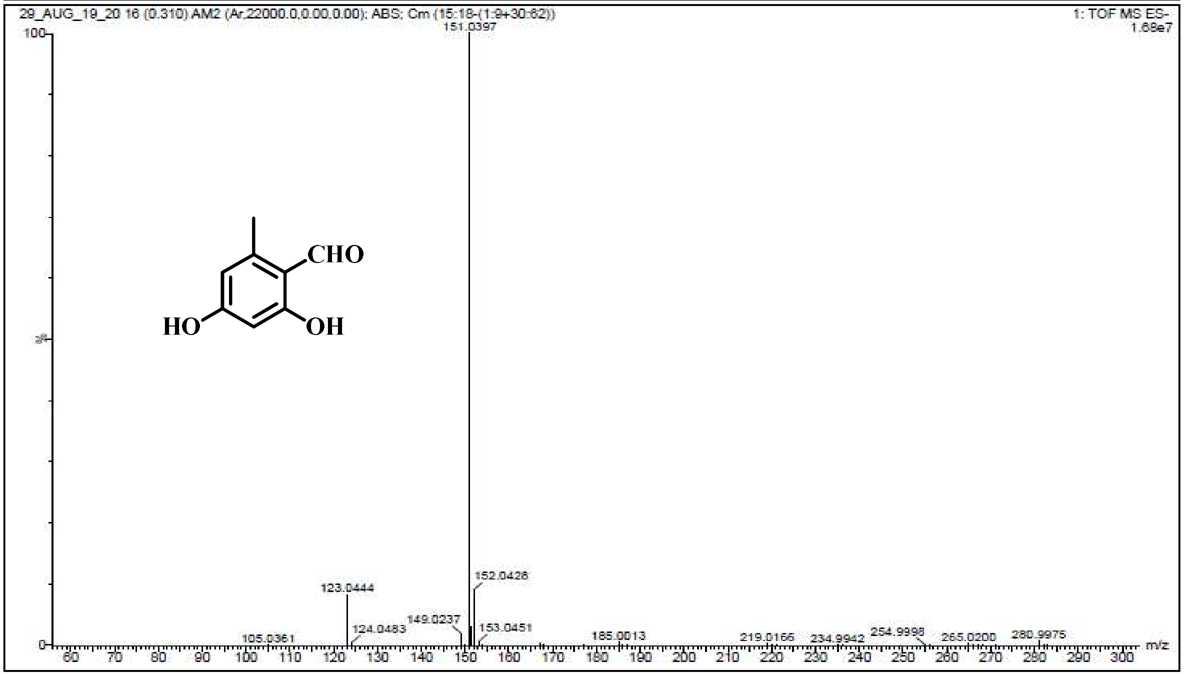

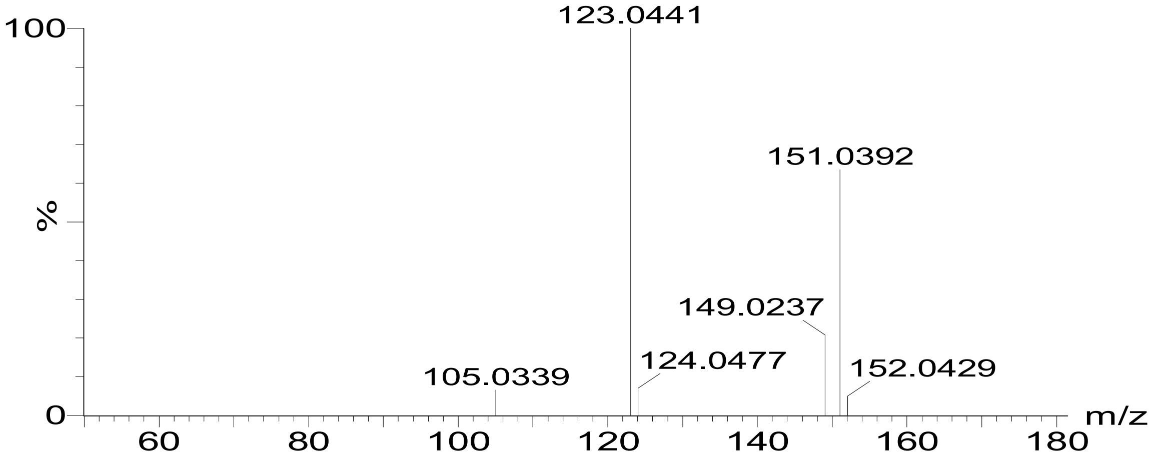

Figure -S22: HRESIMS, MS/MS spectra and fragmentation pattern of Atranol (19)

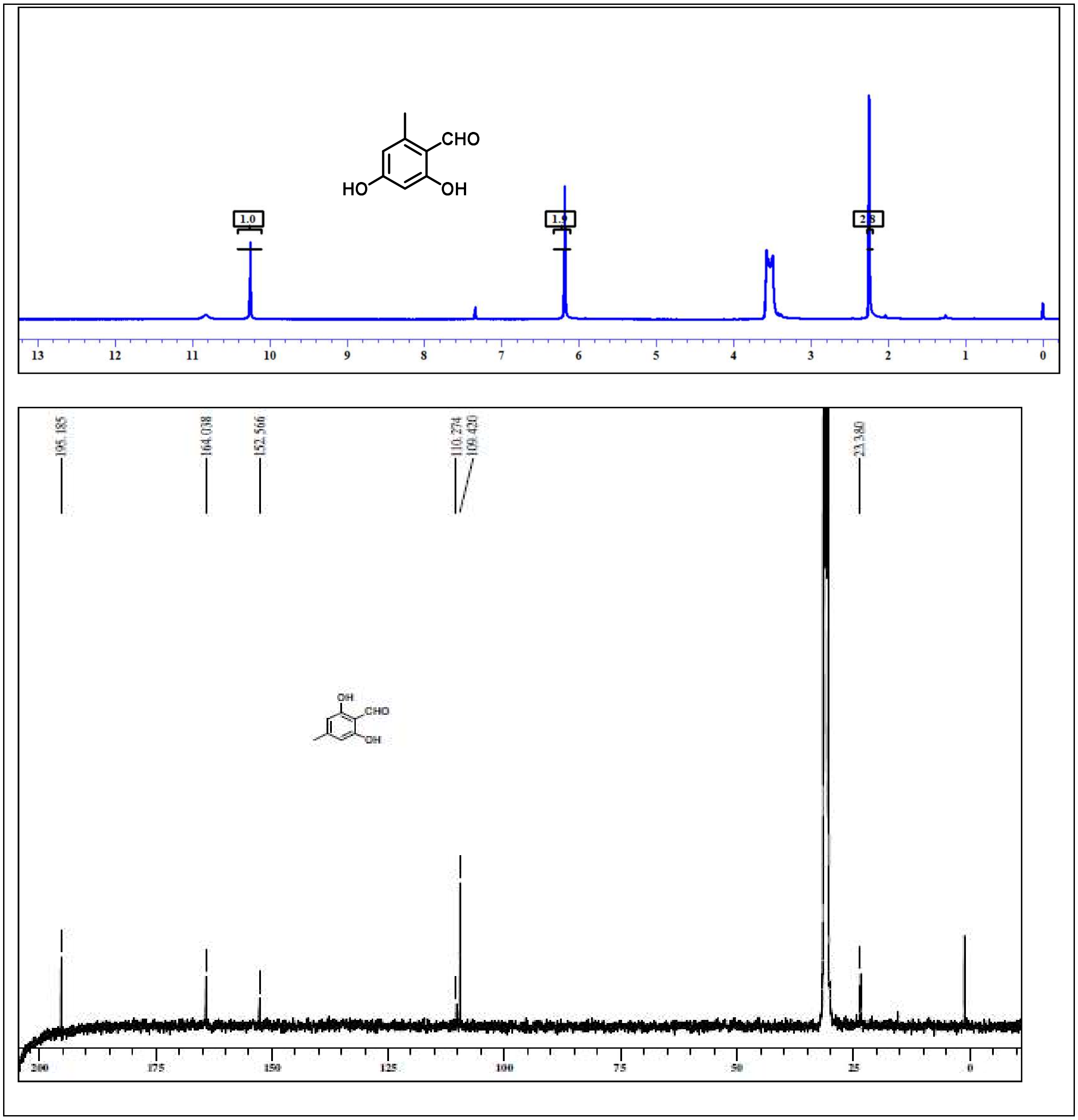

Figure -S23:^{1}H &^{13}C NMR Spectrum of Atranol (**19**) (300 & 75 MHz, Acetone-d_6)

Minimum:				-1.5				
Maximum:		5.0	5.0	50.0				
Mass	Calc. Mass	mDa	PPM	DBE	i-FIT	Norm	Conf(%)	Formula
195.0654	195.0657	-0.3	-1.5	5.5	669.4	n/a	n/a	C10 H11 O4

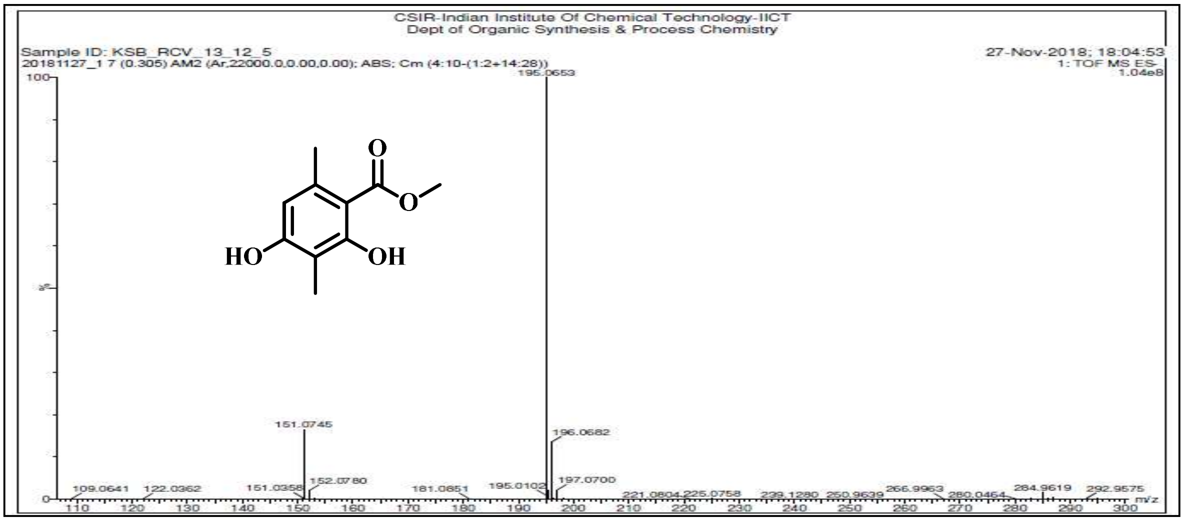

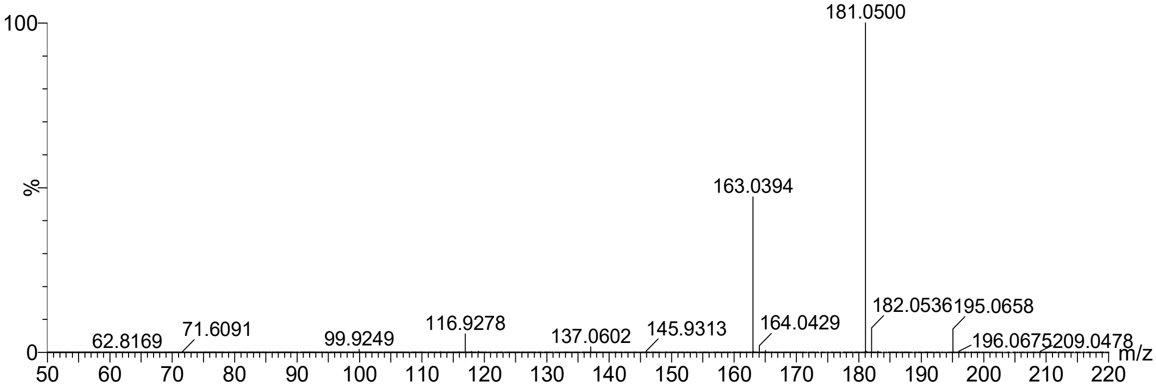

Figure -S24: HRESIMS, MS/MS spectra and fragmentation pattern of Methyl atratate (20)

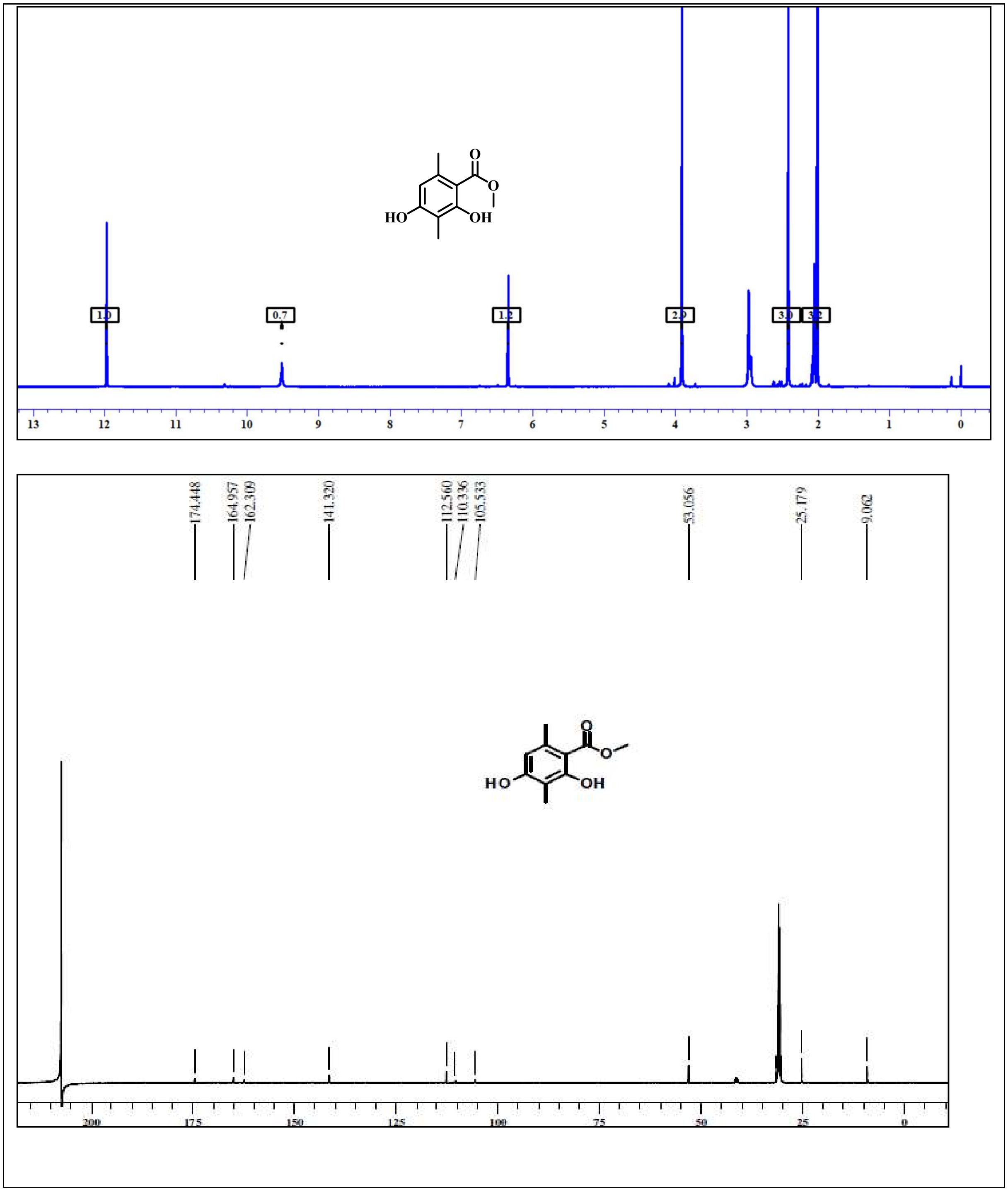

Figure- S25:^{1}H &^{13}C NMR Spectrum of Methyl atratate (20) (400 & 100 MHz, Acetone-d_6)

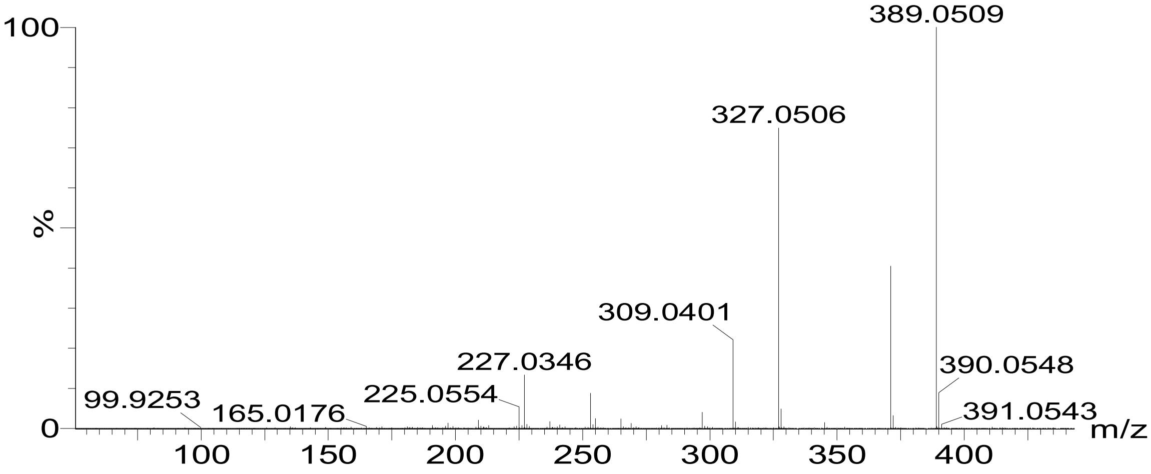

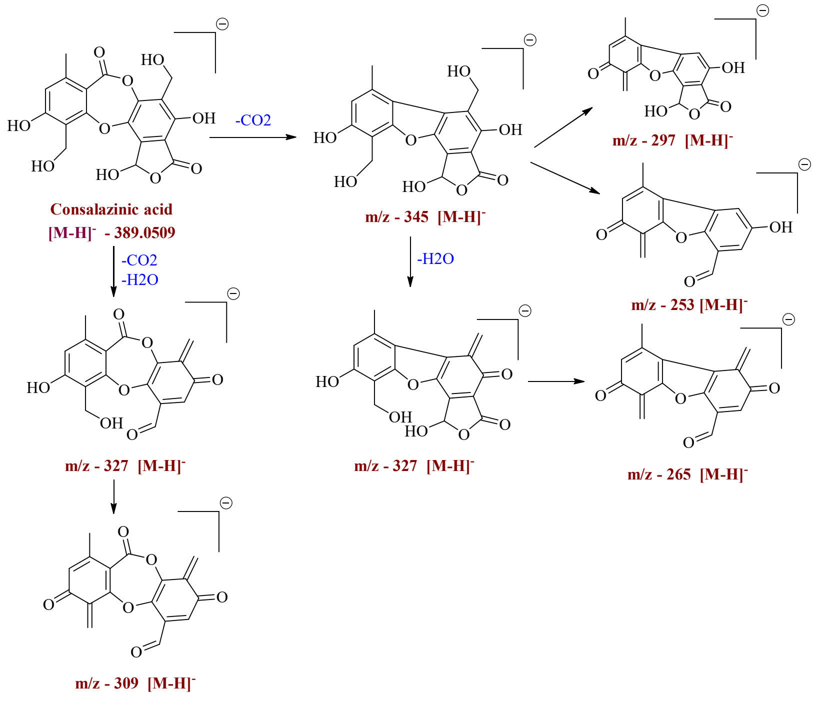

Figure -S26: MS/MS spectra and fragmentation pattern of Consalazinic acid (**1**)

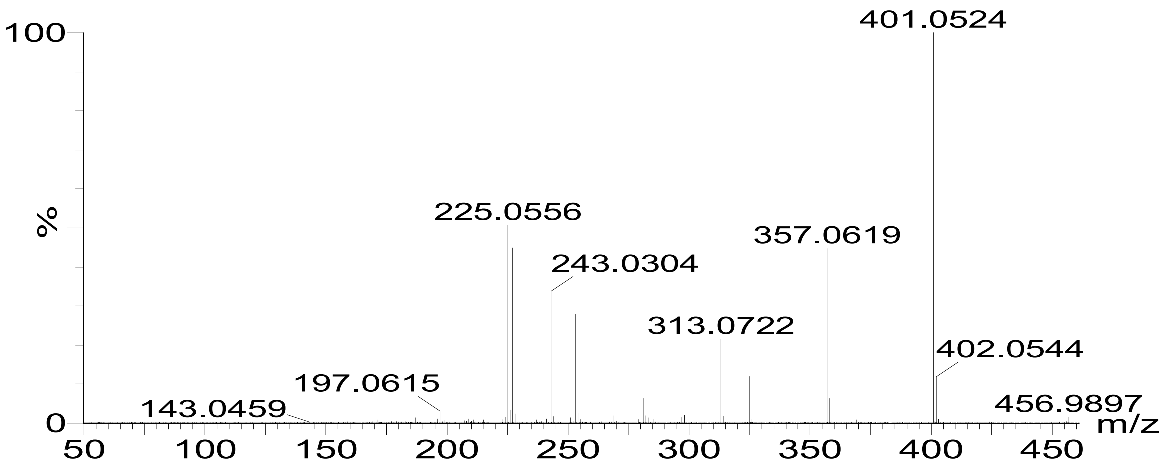

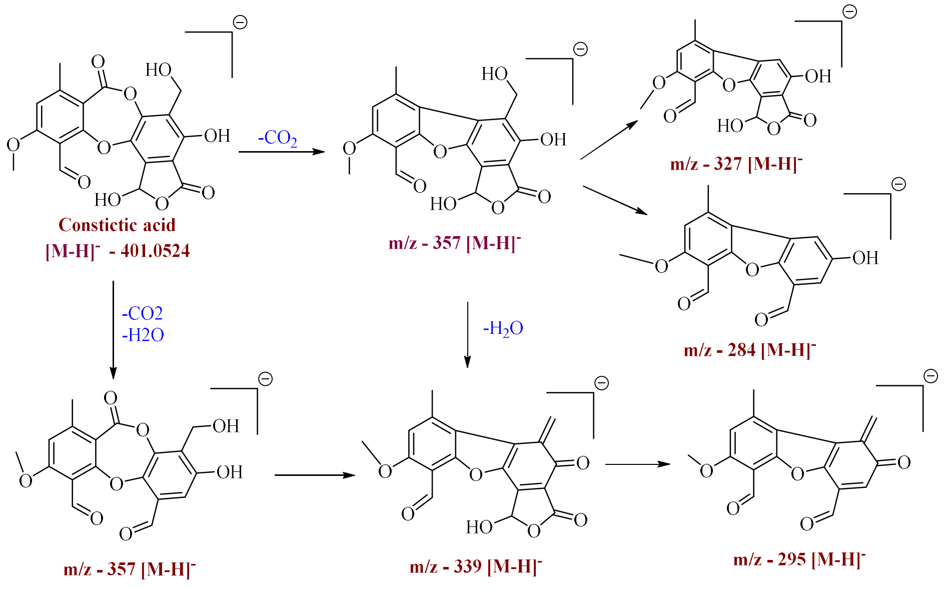

Figure -S27: MS/MS spectra and fragmentation pattern of Constictic acid (**2**)

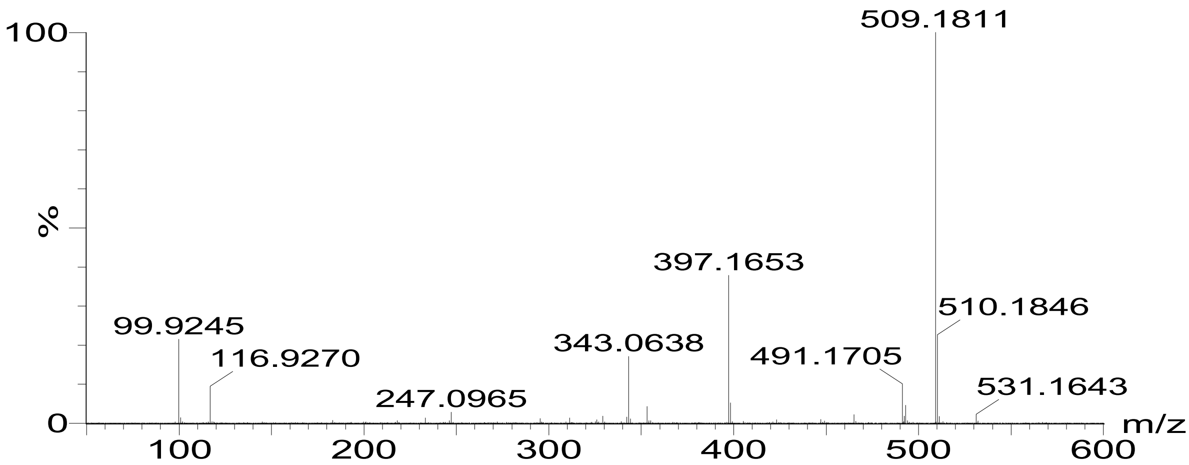

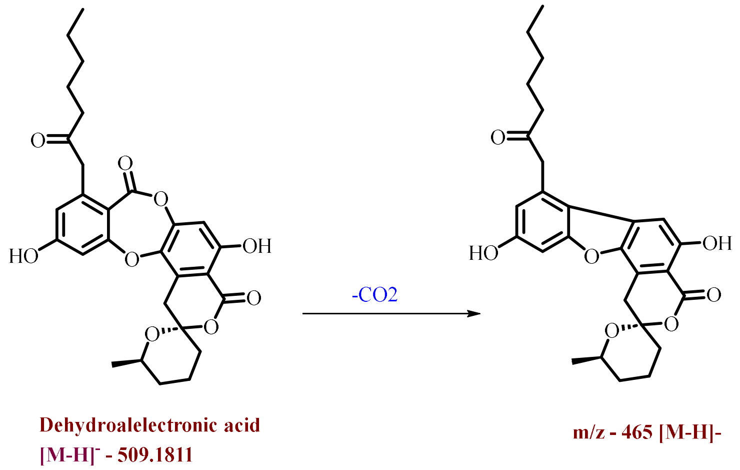

Figure -S28: MS/MS spectra and fragmentation pattern of Dehydroalelectronic acid (3)

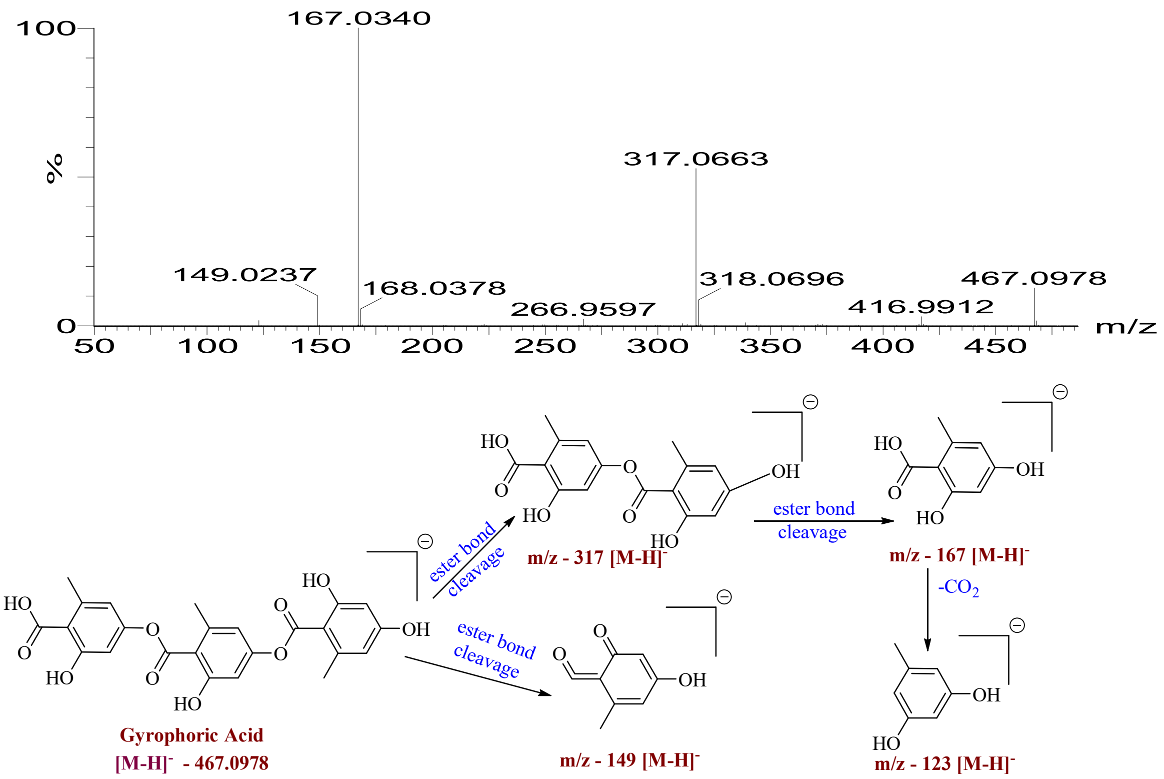

Figure- S29: MS/MS spectra and fragmentation pattern of Gyrophoric acid (4)

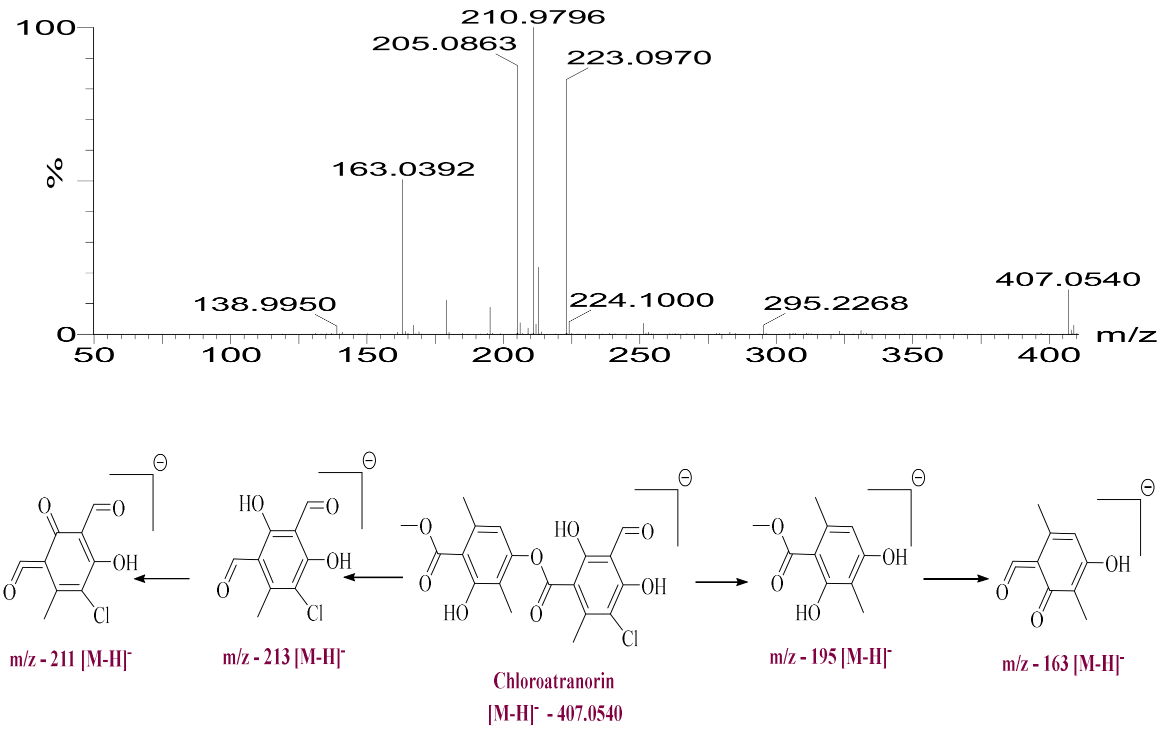

Figure- S30: MS/MS spectra and fragmentation pattern of Chloroatranorin (5)

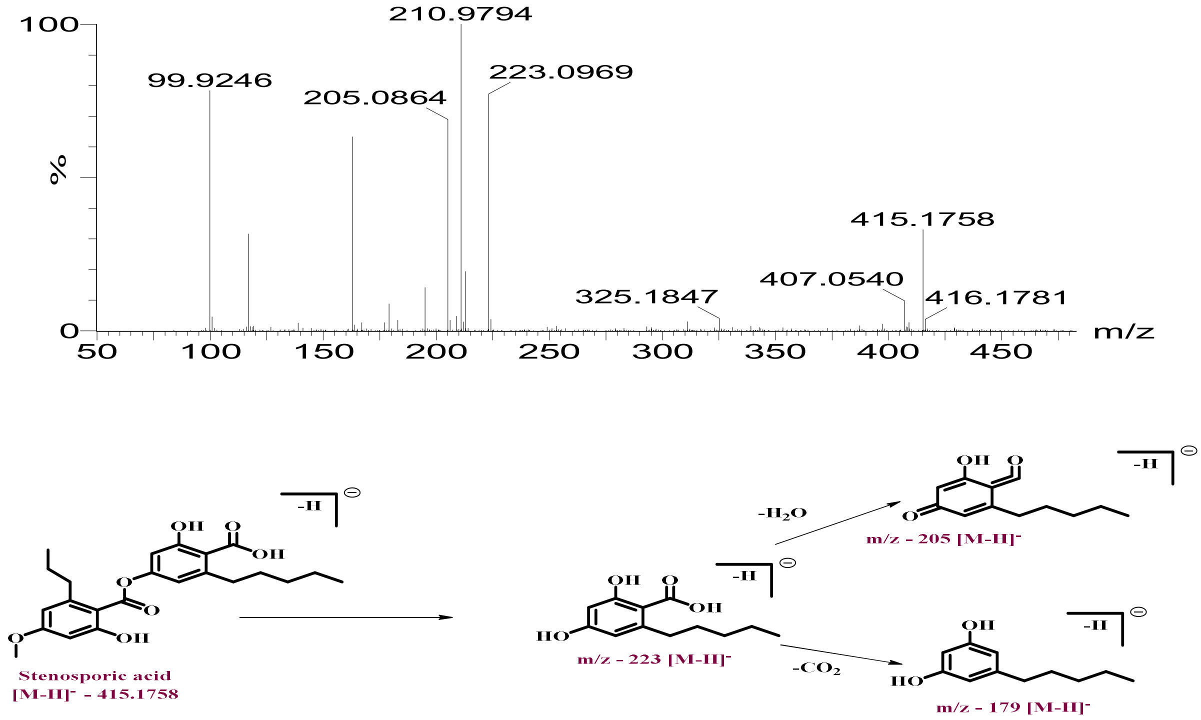

Figure -S31: MS/MS spectra and fragmentation pattern of Stenosporic acid (6)

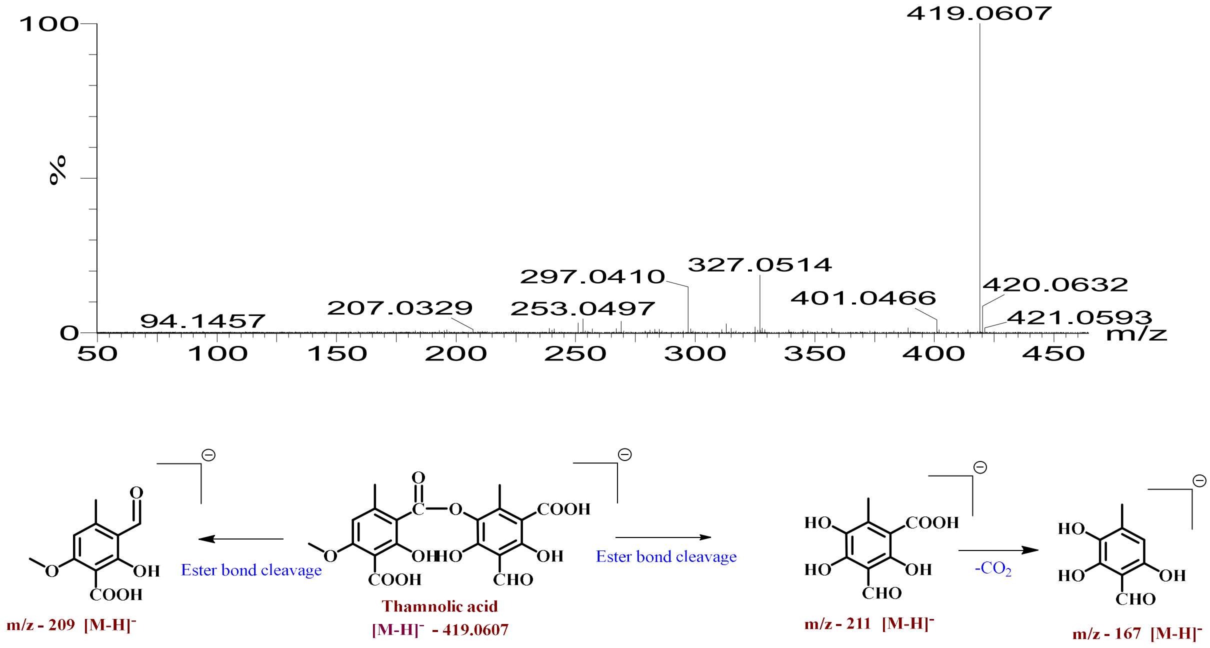

Figure -S32: MS/MS spectra and fragmentation pattern of Thamnolic acid (7)

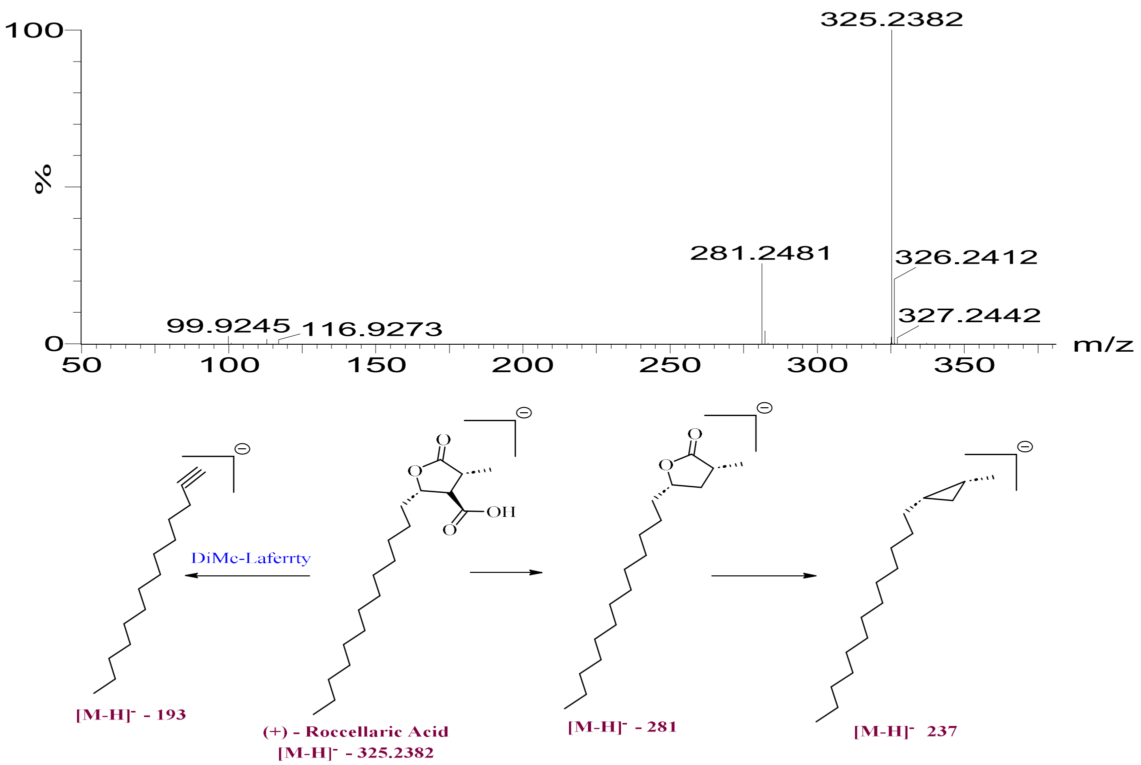

Figure -S33: MS/MS spectra and fragmentation pattern of Roccellaric acid (8)

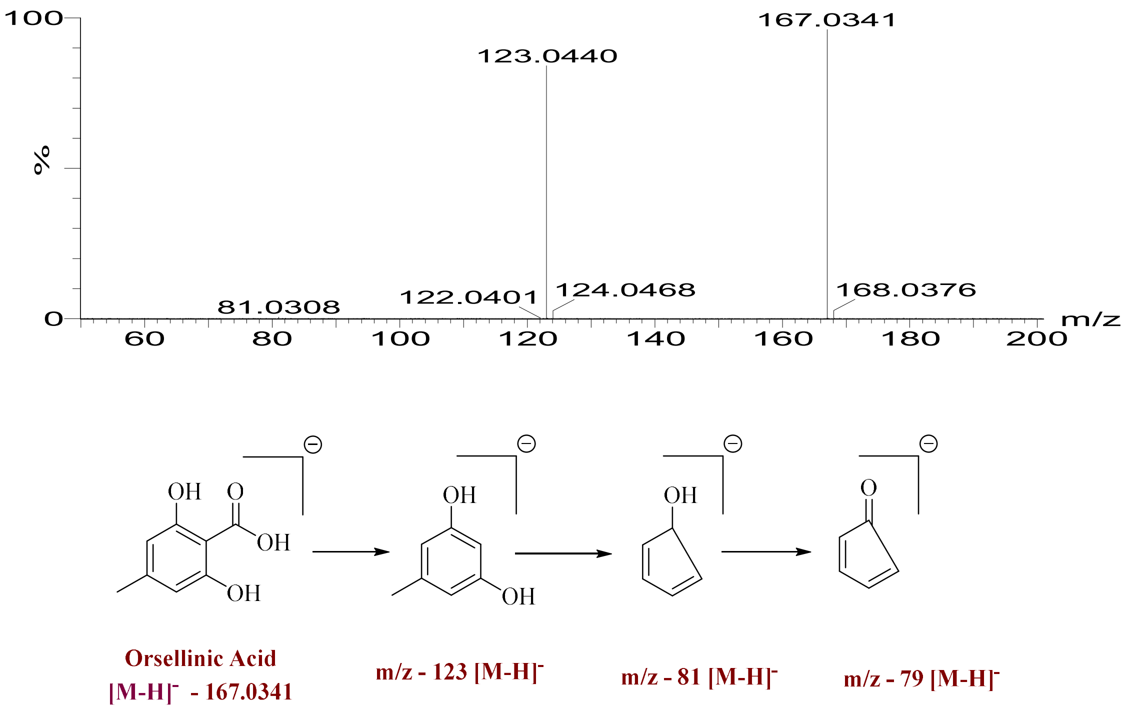

Figure- S34: MS/MS spectra and fragmentation pattern of Orsellinic acid (9)

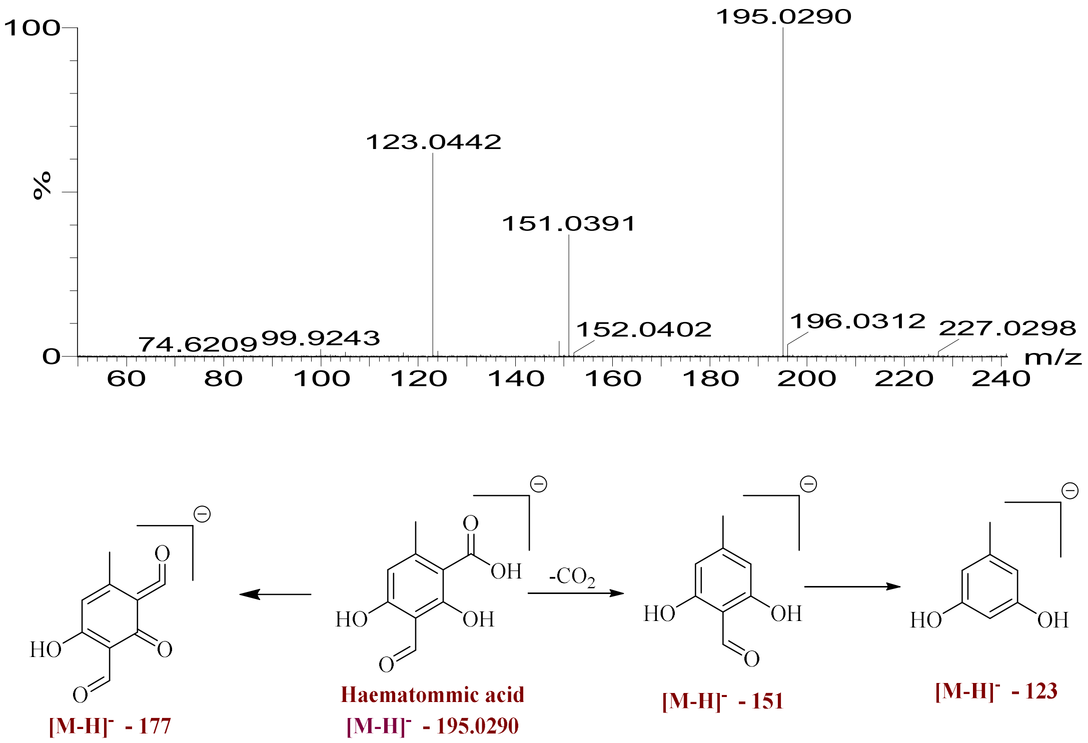

Figure -S35: MS/MS spectra and fragmentation pattern of Haematommic acid (10)

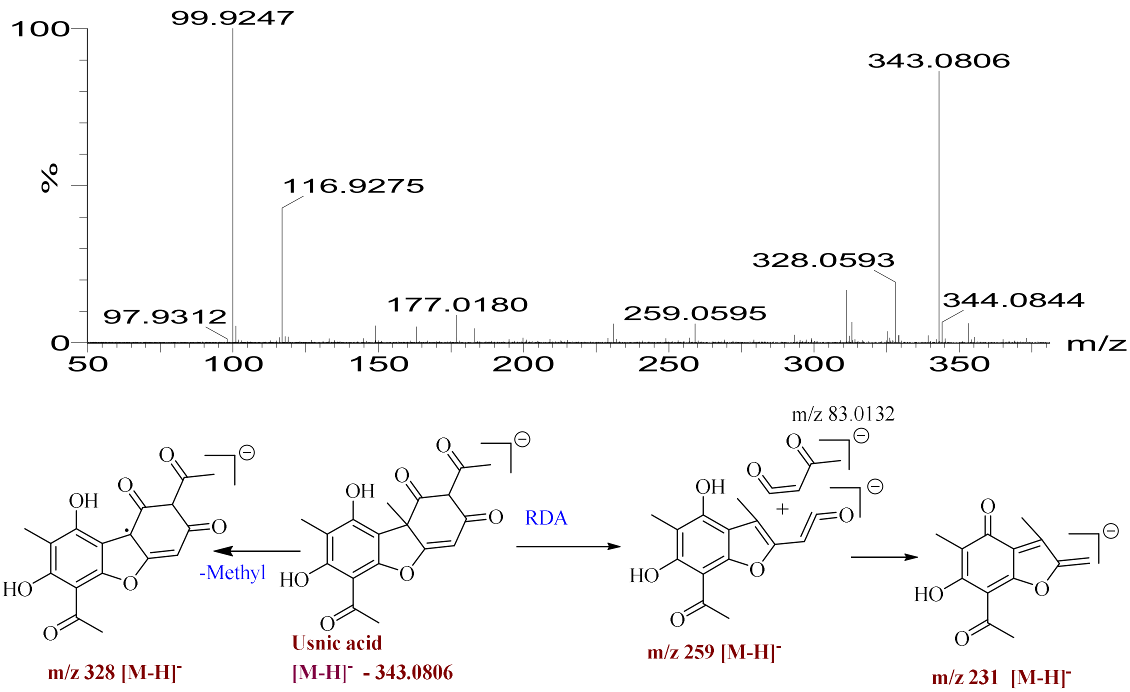

Figure-S36: MS/MS spectra and fragmentation pattern of Usnic acid (**21**)

Table-S1: Antihyperglycemic and antioxidant activities of isolates from H. cirrhata.

**Compound name (2mg/ml)**	**ABTS % Inhibition (IC_{50}) µg/ml**	**DPPH % Inhibition (IC_{50}) µg/ml**	**Intestinal α glucosidase % inhibition (IC_{50}) µg/ml**	**DNA damage assay (% protection)**
EC extract	95.79±2.13	98.23±2.02	61.23±0.46	89.63±0.89
Salazinic acid(1**1**)	79.28±2.07 (2.11±0.04)	54.20±3.03	82.10±1.76 (1.62±0.07)	95.44±0.31
Norlobaridone (1**2**)	99.95±0.39 (0.59±0.02)	21.57±1.32	82.80±1.96 (1.41±0.01)	82.80±9.93
Atranorin (1**3**)	99.99±0.51 (0.068±0.02)	55.91±0.46	76.69±3.6 (2.07±0.01)	96.87±4.30
Lecanoric acid (1**4**)	98.55±.061 (0.32±0.02)	16.45±1.71	55.61±1.27	99.49±0.56
Lichesterinic acid (1**5**)	16.37±1.35	47.31±2.49	48.40±1.56	90.55±2.47
(+)Protolichesterinic acid (1**6**)	22.88±0.90	29.45±1.87	44.79±0.78	90.45±0.02
Methyl hematommate1(7)	94.70±0.48 (0.53±0.03	54.03±2.96	35.64±0.78	98.39±0.11
Iso- rhizonic acid, (1**8**)	97.96±1.41 (16.37±0.06)	45.05±1.79	75.31±3.92 (1.99±0.03)	91.48±0.30
Atranol(1**9**)	99.23±0.51 (0.11±0.07)	78.46±1.12 (1.73±0.02)	28.71±0.54	94.16±2.45
Methyl atrarate(**20**)	96.87±1.02 (1.44±0.05)	63.02±3.66 (3.02±0.07)	33.14±1.17	92.15±0.35
Ascorbic acid (2mg/ml)	99.05±0.62 (0.079±0.01)	83.68±2.72 (1.59±0.05)		-
Acarbose (2mg/ml)	-	±	67.40±3.08 (2.58±0.85)	-

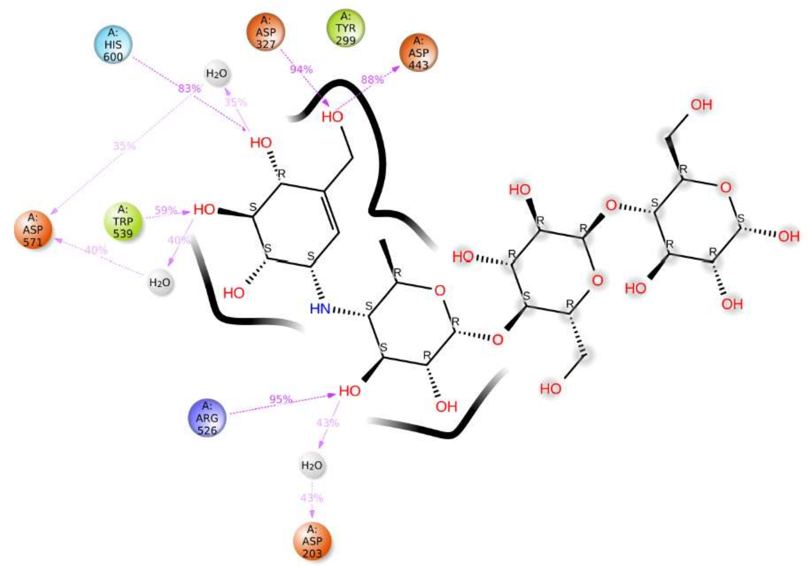

Figure- S37. Percentage interaction plot of Acarbose with catalytic residues

a

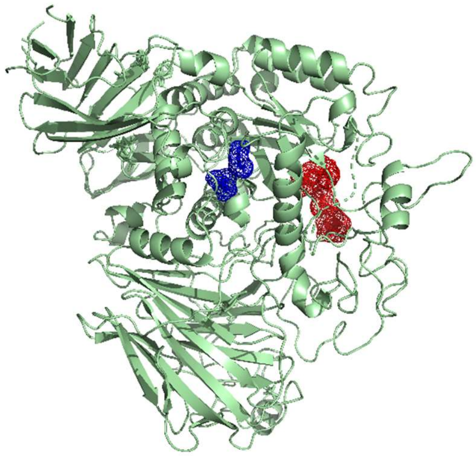

Dock score (kcal/mole)

Cpd	Pocket1	Pocket2
**Acarbose**	-16.074	-9.34
**1**	-6.288	-7.532
**2**	-9.342	-7.204

MM-GBSA binding energy (kcal/mole)

Cpd	Pocket1	Pocket2
**Acarbose**	-25.97	-30.59
**1**	-55.41	-47.93
**2**	-60.78	-49.77

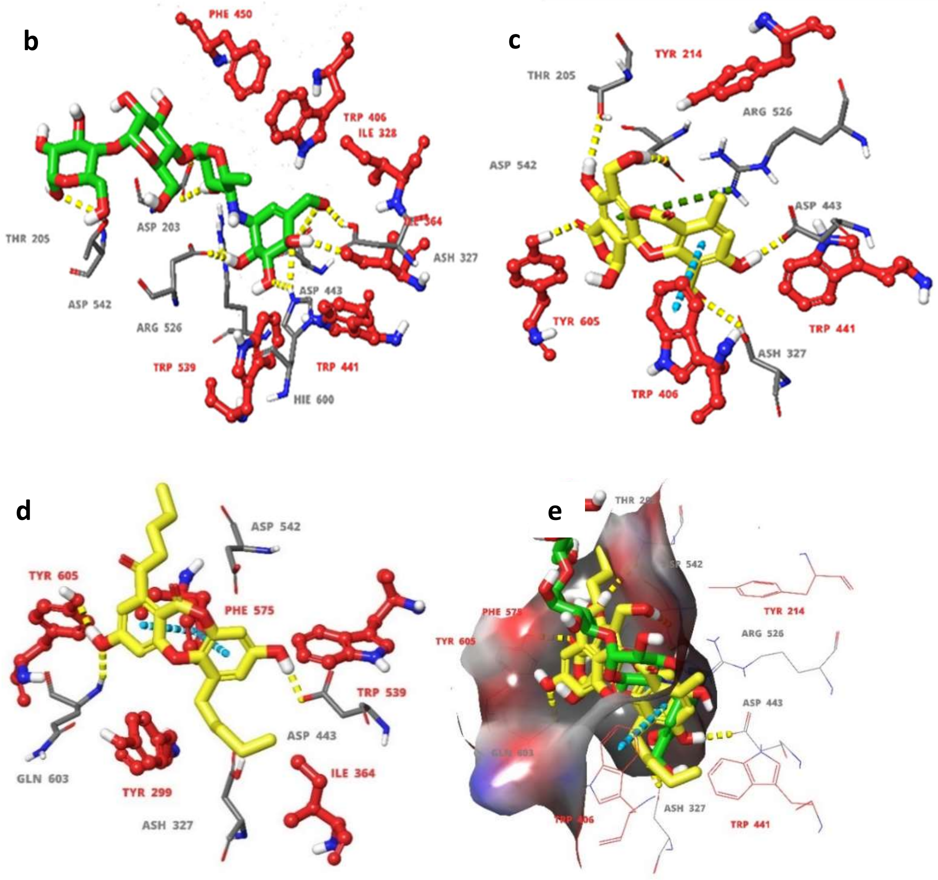

Figure-S38. a) Binding pocket of α-glucosidase catalytic (red) and allosteric pocket (blue); b, c, d) interaction of acarbose (green stick), salazinic acid and norlobaridone (yellow sticks) with catalytic residues (pocket 1) respectively; e) overlap of acarbose, salazinic acid and norlobaridone. Hydrogen bonds (yellow dotted lines); π-π stackings (blue dotted lines), π-cation (green dotted lines) and red ball & sticks/wires/other macromodel sticks represents active residues.

a b

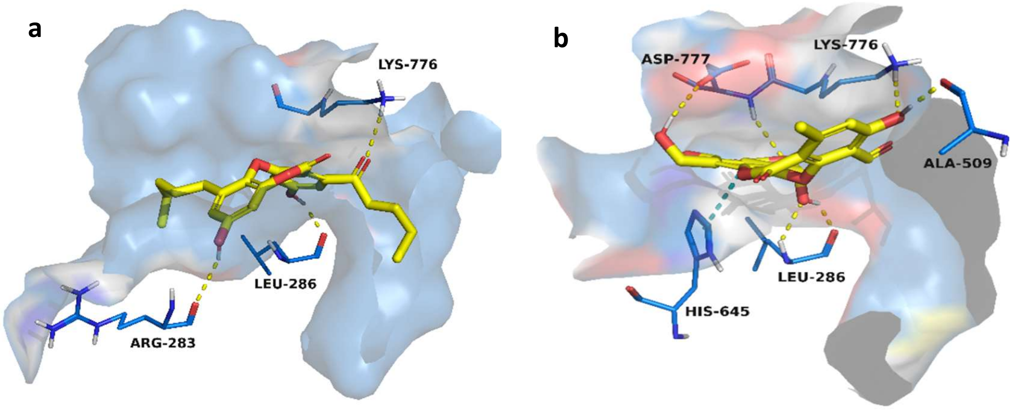

Figure- S39. a) Norlobaridone (**2**) and its alkyl chain occupied in deeper in allosteric pocket grove; b) Salazinic acid (**1**) and its interaction regimen. Hydrogen bonds (yellow dotted lines); π-π stackings (blue dotted lines), π-cation (green dotted lines) and red ball & sticks/wires/other macromodel sticks represents active residues.

a

b

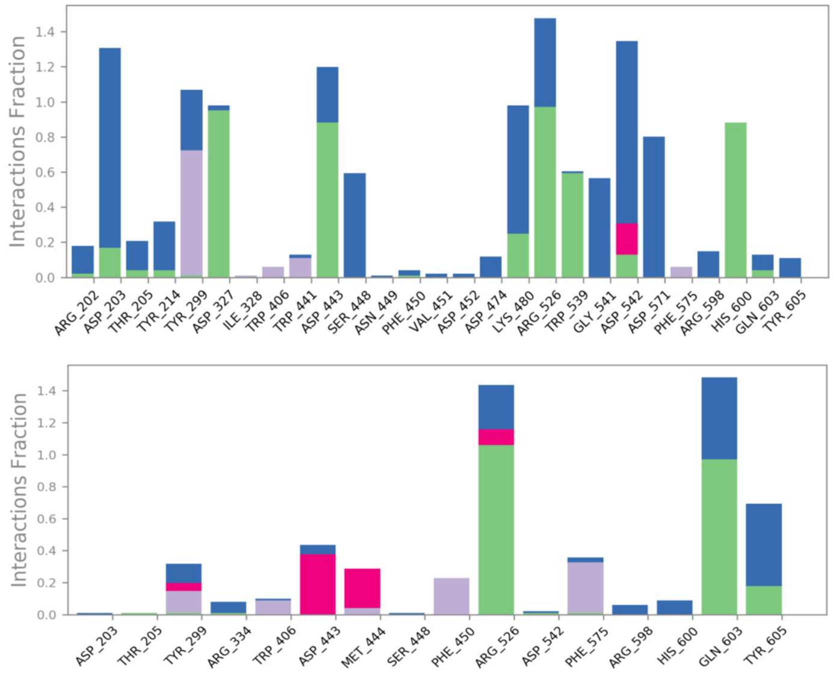

c

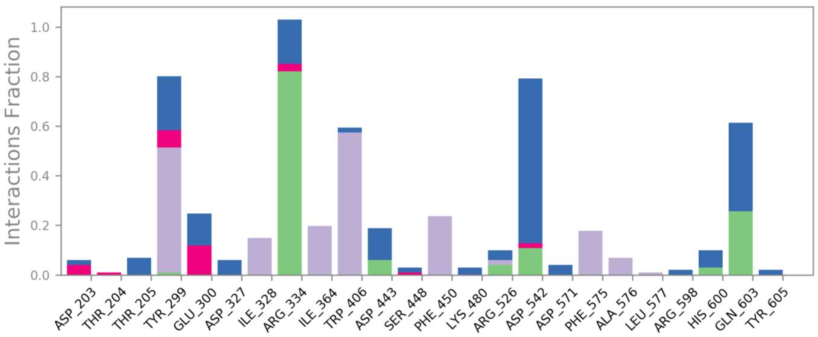

d

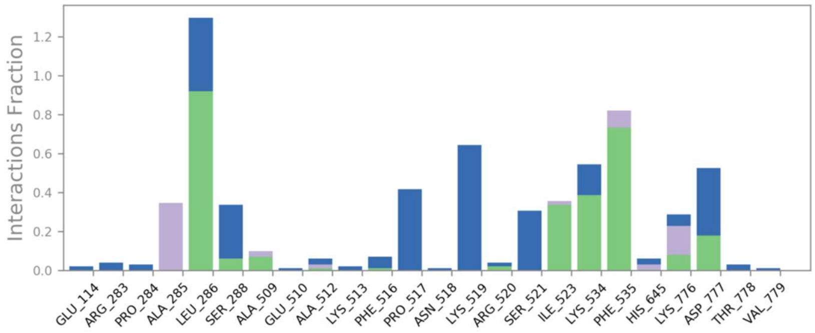

e

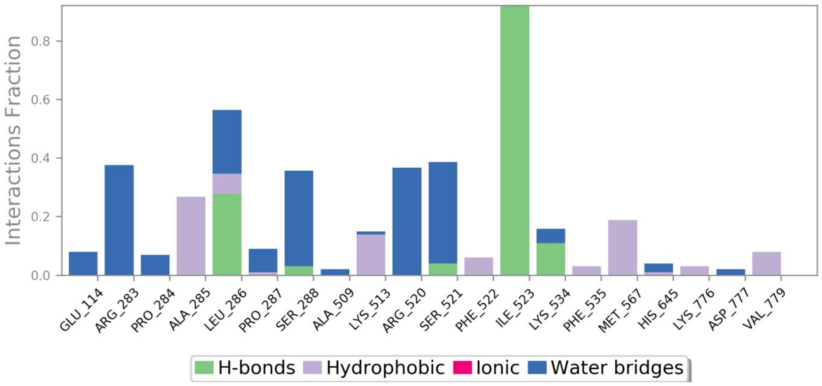

Figure- S40. a, b & c) Interaction network of acarbose (a), salazinic acid (b) and norlobaridone (c) in 100 simulation with active pocket. (d) & (e) Interaction network of salazinic acid and norlobaridone, respectively, with allosteric pocket residues.
